# A Simple Approximation Method for the Fisher–Rao Distance between Multivariate Normal Distributions

**DOI:** 10.3390/e25040654

**Published:** 2023-04-13

**Authors:** Frank Nielsen

**Affiliations:** Sony Computer Science Laboratories, Tokyo 141-0022, Japan; frank.nielsen.x@gmail.com

**Keywords:** Fisher–Rao normal manifold, symmetric positive–definite matrix cone, isometric embedding, information geometry, exponential family, elliptical distribution, maximal invariant

## Abstract

We present a simple method to approximate the Fisher–Rao distance between multivariate normal distributions based on discretizing curves joining normal distributions and approximating the Fisher–Rao distances between successive nearby normal distributions on the curves by the square roots of their Jeffreys divergences. We consider experimentally the linear interpolation curves in the ordinary, natural, and expectation parameterizations of the normal distributions, and compare these curves with a curve derived from the Calvo and Oller’s isometric embedding of the Fisher–Rao *d*-variate normal manifold into the cone of (d+1)×(d+1) symmetric positive–definite matrices. We report on our experiments and assess the quality of our approximation technique by comparing the numerical approximations with both lower and upper bounds. Finally, we present several information–geometric properties of Calvo and Oller’s isometric embedding.

## 1. Introduction

### 1.1. The Fisher–Rao Normal Manifold

Let Sym(d) be the set of d×d symmetric matrices with real entries and P(d)⊂Sym(d) denote the set of symmetric positive–definite d×d matrices that forms a convex regular cone. Let us denote by $N\left(d\right)=\{N\left(\mu ,\Sigma \right)\;:\;\left(\mu ,\Sigma \right)\in \Lambda \left(d\right)=R^{d}\times P\left(d\right)\}$ the set of *d*-variate normal distributions, MultiVariate Normals or MVNs for short, also called Gaussian distributions. A MVN distribution N(μ,Σ) has probability density function (pdf) on the support $R^{d}$:

$$p_{\lambda =(\mu ,\Sigma )}\left(x\right)={\left(2\pi \right)}^{{}^{\frac{d}{2}}}{\left|\Sigma \right|}^{-\frac{1}{2}}\exp\left(-\frac{1}{2}{\left(x-\mu \right)}^{⊤}\Sigma^{-1}\left(x-\mu \right)\right),\;x\in R^{d},$$

where |M|=det(M) denotes the determinant of matrix *M*.

The statistical model N(d) is of dimension $m=\dim\left(\Lambda \left(d\right)\right)=d+\frac{d(d+1)}{2}=\frac{d(d+3)}{2}$ since it is identifiable, i.e., there is a one-to-one correspondence $\lambda ↔p_{\lambda }\left(x\right)$ between λ∈Λ(d) and N(μ,Σ)∈N(d). The statistical model N(d) is said to be regular since the second-order derivatives $\frac{\partial^{2}p_{\lambda }}{\partial \lambda_{i}\partial \lambda_{j}}$ and third-order derivatives $\frac{\partial^{3}p_{\lambda }}{\partial \lambda_{i}\partial \lambda_{j}\partial \lambda_{k}}$ are smooth functions (defining the metric and cubic tensors in information geometry [1]), and the set of first-order partial derivatives $\left\{\frac{\partial p_{\lambda }}{\partial \lambda_{1}},\ldots ,\frac{\partial p_{\lambda }}{\partial \lambda_{1}}\right\}$ are linearly independent.

Let Cov(X) denote the covariance of *X* (variance when *X* is scalar). A matrix *M* is a semi-positive–definite if and only if $\forall x\neq 0,x^{⊤}Mx\geq 0$. The Fisher information matrix [1,2] (FIM) is the following symmetric semi-positive–definite matrix:

$$I\left(\lambda \right)=\mathrm{Cov}[\nabla \log p_{\lambda }\left(x\right)]⪰0.$$

For regular statistical models $\left\{p_{\lambda }\right\}$, the FIM is positive–definite: I(λ)≻0, i.e., $\forall x\neq 0,x^{⊤}I\left(\lambda \right)x>0$. $M_{1}≻M_{2}$ denotes Löwner partial ordering, i.e., the fact that $M_{1}-M_{2}$ is positive–definite.

The FIM is covariant under the reparameterization of the statistical model [2]. That is, let θ(λ) be a new parameterization of the MVNs. Then we have:

$$I_{\theta }\left(\lambda \right)={\left[\frac{\partial \lambda }{\partial \theta }\right]}^{⊤}\;\times \;I_{\lambda }\left(\lambda \left(\theta \right)\right)\;\times \;\left[\frac{\partial \lambda }{\partial \theta }\right].$$

For example, we may parameterize univariate normal distributions by $\lambda =(\mu ,\sigma^{2})$ or θ=(μ,σ). We obtain the following Fisher information matrices for these parameterizations:

$$I_{\lambda }\left(\lambda \left(\mu ,\sigma \right)\right)=\left[\begin{array}{cc} \frac{1}{\sigma^{2}} & 0\\ 0 & \frac{1}{2\sigma^{4}} \end{array}\right]\;\text{and}\;I_{\theta }\left(\theta \left(\mu ,\sigma \right)\right)=\left[\begin{array}{cc} \frac{1}{\sigma^{2}} & 0\\ 0 & \frac{1}{2\sigma^{2}} \end{array}\right].$$

In higher dimensions, parameterization $\lambda =(\mu ,\sigma^{2})$ corresponds to the parameterization (μ,Σ) while parameterization θ=(μ,L) where $\Sigma =LL^{⊤}$ is the unique Cholesky decomposition with L∈GL(d), the group of invertible d×d matrices. Another useful parameterization for optimization is the log–Cholesky parameterization [3] ($\eta =\left(\mu ,\log\sigma^{2}\right)\in R^{2}$ for univariate normal distributions) which ensures that a gradient descent always stays in the domain. The Fisher information matrix with respect to the log–Cholesky parameterization is $I_{\eta }\left(\eta \left(\mu ,\sigma \right)\right)=\left[\begin{array}{cc} \frac{1}{\sigma^{2}} & 0\\ 0 & 2 \end{array}\right]$ with $\eta \left(\mu ,\sigma \right)\in R^{2}$.

Since the statistical model N(d) is identifiable and regular, the Fisher information matrix can be written equivalently as follows [2,4]:

(1) $$\begin{array}{ccc} I\left(\mu ,\Sigma \right)=\mathrm{Cov}\left[\nabla \log p_{\left(\mu ,\Sigma \right)}\right] & = & E_{p_{\left(\mu ,\Sigma \right)}}\left[\nabla \log p_{\left(\mu ,\Sigma \right)}\nabla \log p_{\left(\mu ,\Sigma \right)}^{⊤}\right], \end{array}$$

(2) $$\;\;\;\;\;\;\;\;\;\;\;\;\;\;\;\;\;\;\;\;\;\;\;\;\;\;\;\;\;\;\;\;\;\;\;=-E_{p_{\left(\mu ,\Sigma \right)}}\left[\nabla^{2}\log p_{\left(\mu ,\Sigma \right)}\right].$$

For multivariate distributions parameterized by a *m*-dimensional vector (with $m=\frac{d(d+3)}{2}$)

$$\theta =\left(\theta_{1},\ldots ,\theta_{d},\theta_{d+1},\ldots ,\theta_{m}\right)\in R^{m},$$

with $\mu =(\theta_{1},\ldots ,\theta_{d})$ and $\Sigma \left(\theta \right)=\mathrm{vech}\left(\theta_{d+1},\ldots ,\theta_{m}\right)$ (inverse half-vectorization of matrices [5]), we have [6,7,8,9]:

$$I\left(\theta \right)=\left[I_{ij}\left(\theta \right)\right],\;\text{with}\;I_{ij}\left(\theta \right)={\left[\frac{\partial \mu }{\partial \theta_{i}}\right]}^{⊤}\Sigma^{-1}\frac{\partial \mu }{\partial \theta_{j}}+\frac{1}{2}\mathrm{tr}\left(\Sigma^{-1}\frac{\partial \mu }{\partial \theta_{i}}\Sigma^{-1}\frac{\partial \mu }{\partial \theta_{j}}\right).$$

By equipping the regular statistical model N(d) with the Fisher information metric

$$g_{N}^{\mathrm{Fisher}}\left(\mu ,\Sigma \right)=\mathrm{Cov}\left[\nabla \log p_{\left(\mu ,\Sigma \right)}\left(x\right)\right]$$

we obtain a Riemannian manifold $M=M_{N}$ called the Fisher–Rao Gaussian or normal manifold [6,7]. The tangent space $T_{N}M$ is identified with the product space $R^{d}\times \mathrm{Sym}\left(d\right)$. Let {∂μ,∂Σ} be a natural vector basis in $T_{N}M$, and denote by [v] and [V] the vector components in that natural basis. We have

$$\begin{array}{ccc} g_{\left(\mu ,\Sigma \right)}^{\mathrm{Fisher}}\left(\left(v_{1},V_{1}\right),\left(v_{2},V_{2}\right)\right) & = & {\langle \left(v_{1},V_{1}\right),\left(v_{2},V_{2}\right)\rangle }_{\left(\mu ,\Sigma \right)},\\ & = & {\left[v_{1}\right]}^{⊤}\Sigma^{-1}\left[v_{2}\right]+\frac{1}{2}\mathrm{tr}\left(\Sigma^{-1}\left[V_{1}\right]\Sigma^{-1}\left[V_{2}\right]\right). \end{array}$$

The induced Riemannian geodesic distance $\rho_{N}\left(\cdot ,\cdot \right)$ is called the Rao distance [10] or the Fisher–Rao distance [11,12]:

(3) $$\rho_{N}\left(N\left(\lambda_{1}\right),N\left(\lambda_{2}\right)\right)=\underset{\begin{array}{c} c(t)\\ c\left(0\right)=p_{\lambda_{1}}\\ c\left(1\right)=p_{\lambda_{2}} \end{array}}{\inf}\;\left\{\mathrm{Length}(c)\right\},$$

where the Riemannian length of any smooth curve c(t)∈M is defined by

(4) $$\mathrm{Length}\left(c\right)=\int_{0}^{1}\sqrt{{\langle \dot{c}\left(t\right),\dot{c}\left(t\right)\rangle }_{c(t)}}dt=\int_{0}^{1}ds_{N}\left(t\right)dt=\int_{0}^{1}{∥\dot{c}\left(t\right)∥}_{c(t)}dt,$$

where $\dot{\;}=\frac{d}{dt}$ denotes the derivative with respect to parameter *t*, $ds_{N}\left(t\right)$ is the Riemannian length element of $\left(M,g_{N}^{\mathrm{Fisher}}\right)$ and ${∥\cdot ∥}_{c(t)}=\sqrt{{\langle \cdot ,\cdot \rangle }_{c(t)}}$. We also write $\rho_{N}\left(p_{\lambda_{1}},p_{\lambda_{2}}\right)$ for $\rho_{N}\left(N\left(\lambda_{1}\right),N\left(\lambda_{2}\right)\right)$.

The minimizing curve $c\left(t\right)=\gamma_{N}\left(p_{\lambda_{1}},p_{\lambda_{2}};t\right)$ of Equation (3) is called the Fisher–Rao geodesic. The Fisher–Rao geodesic is also an autoparallel curve [2] with respect to the Levi–Civita connection $\nabla_{N}^{\mathrm{Fisher}}$ induced by the Fisher metric $g_{N}^{\mathrm{Fisher}}$.

**Remark 1.**  *If we consider the Riemannian manifold (M,βg) for β>0 instead of (M,g) then the length element ds is scaled by $\sqrt{\beta }$: $ds_{\beta g}=\beta \;ds_{g}$. It follows that the length of a curve c becomes*

$${\mathrm{Length}}_{\beta g}\left(c\right)=\sqrt{\beta }\;{\mathrm{Length}}_{g}\left(c\right).$$

*However, the geodesics joining any two points $p_{1}$ and $p_{2}$ of M are the same: $\gamma_{\beta g}\left(p_{1},p_{2};t\right)=\gamma_{g}\left(p_{1},p_{2};t\right)$ (with $\gamma_{g}\left(p_{1},p_{2};0\right)=p_{1}$ and $\gamma_{g}\left(p_{1},p_{2};1\right)=p_{2}$).*

Historically, Hotelling [13] first used this Fisher Riemannian geodesic distance in the late 1920s. From the viewpoint of information geometry [1], the Fisher metric is the unique Markov invariant metric up to rescaling [14,15,16]. The counterpart to the Fisher metric on the compact manifold has been reported in [17], proving its uniqueness under the action of the diffeomorphism group. The Fisher–Rao distance has been used to design statistical hypothesis testing [18,19,20,21], to measure the distance between the prior and posterior distributions in Bayesian statistics [22], in clustering [23,24], in signal processing [25,26,27,28], and in deep learning [29], just to mention a few.

The squared line element induced by the Fisher metric of the multivariate normal family [6,7] is

(5) $$\begin{array}{ccc} ds_{N}^{2}\left(\mu ,\Sigma \right) & = & {\left[\begin{array}{c} d\mu \\ d\Sigma \end{array}\right]}^{⊤}\;I\left(\mu ,\Sigma \right)\;\left[\begin{array}{c} d\mu \\ d\Sigma \end{array}\right],\\ & = & d\mu^{⊤}\Sigma^{-1}d\mu +\frac{1}{2}\mathrm{tr}\left({\left(\Sigma^{-1}d\Sigma \right)}^{2}\right). \end{array}$$

There are many ways to calculate the FIM/length element for multivariate normal distributions [7,9]. Let us give a simple approach based on the fact that the family N(d) of normal distributions forms a regular exponential family [30]:

$$N\left(d\right)=\left\{p_{\theta (\lambda )}=\exp\left(\langle \theta_{v}\left(\mu \right),x\rangle +\langle \theta_{M}\left(\Sigma \right),xx^{⊤}\rangle -F_{N}\left(\theta_{v},\theta_{M}\right)\right)\right\},$$

with $\theta \left(\lambda \right)=(\theta_{v}=\left(\Sigma^{-1}\mu ,\theta_{M}=\frac{1}{2}\Sigma^{-1}\right)$ the natural parameters and log-partition function (also called cumulant function)

$$F_{N}\left(\theta \right)=\frac{1}{2}\left(d\log\pi -\log|\theta_{M}|+\frac{1}{2}\theta_{v}^{⊤}\theta_{M}^{-1}\theta_{v}\right).$$

The vector inner product is $\langle v_{1},v_{2}\rangle =v_{1}^{⊤}v_{2}$, and the matrix inner product is $\langle M_{1},M_{2}\rangle =\mathrm{tr}\left(M_{1}M_{2}^{⊤}\right)$. The exponential family is said to be regular when the natural parameter space is open. Using Equation (2), it follows that the MVN FIM is $I_{\theta }\left(\theta \right)=-E\left[\nabla^{2}\log p_{\theta }\right]=\nabla^{2}F\left(\theta \right)$. This proves that the FIM is well-defined, i.e., ${\left(I_{\theta }\left(\theta \right)\right)}_{ij}<\infty$. As an exponential family [1], we also have $I_{\theta }\left(\theta \right)=E\left[t\left(x\right)\right]$, where $t\left(x\right)=\left(x,xx^{⊤}\right)$ is the sufficient statistic. Thus, the Fisher metric is a Hessian metric [31]. Let $F_{N}\left(\theta_{v},\theta_{M}\right)=F_{v}\left(\theta_{v}\right)+F_{M}\left(\theta_{M}\right)$ with $F_{v}\left(\theta_{v}\right)=\frac{1}{2}\left(d\log\pi +\frac{1}{2}\theta_{v}^{⊤}\theta_{M}^{-1}\theta_{v}\right)$ and $F_{M}\left(\theta_{M}\right)=-\frac{1}{2}\log\left|\theta_{M}\right|$. We obtain the following block-diagonal expression of the FIM:

$$I\left(\theta \left(\lambda \right)\right)=\nabla^{2}F_{N}\left(\theta \left(\mu ,\Sigma \right)\right)=\left[\begin{array}{cc} \Sigma^{-1} & 0\\ 0 & \frac{1}{2}\nabla_{\theta_{M}}^{2}\log\left|\frac{1}{2}\Sigma^{-1}\right| \end{array}\right].$$

Therefore $ds_{N}^{2}\left(\mu ,\Sigma \right)=ds_{v}^{2}+ds_{M}^{2}$ with $ds_{v}^{2}\left(\mu \right)=d\mu^{⊤}\Sigma^{-1}d\mu$ and $ds_{M}^{2}\left(\Sigma \right)=\frac{1}{2}\mathrm{tr}\left({\left(\Sigma^{-1}d\Sigma \right)}^{2}\right)$. Let us note in passing that $\nabla_{\theta_{M}}^{2}\log\left|\theta_{M}\right|$ is a fourth order tensor [4].

The family N(d) can also be considered to be an elliptical family [32], thus highlighting the affine-invariance property of the Fisher information metric. That is, the Fisher metric is invariant with respect to affine transformations [33]: Let (a,A) be an element of the affine group Aff(d) with $a\in R^{d}$ and A∈GL(d). The group identity element of Aff(d) is e=(0,I) and the group operation is $\left(a_{1},A_{1}\right).\left(a_{2},A_{2}\right)=\left(a_{1}+A_{1}a_{2},A_{1}A_{2}\right)$ with inverse ${\left(a,A\right)}^{-1}=\left(-A^{-1}a,A^{-1}\right)$). Then we have

**Property 1**  (Fisher–Rao affine invariance)**.** *For all $A\in \mathrm{GL}\left(d\right),a\in R^{d}$, we have*

(6) $$\rho_{N}\left(N\left(A\mu_{1}+a,A\Sigma_{1}A^{⊤}\right),N\left(A\mu_{2}+a,A\Sigma_{2}A^{⊤}\right)\right)=\rho_{N}\left(N\left(\mu_{1},\Sigma_{1}\right),N\left(\mu_{2},\Sigma_{2}\right)\right).$$

This can be proven by checking that $ds_{N}\left(\mu^{\prime },\Sigma^{\prime }\right)=ds_{N}\left(\mu ,\Sigma \right)$ where $\mu^{\prime }=A\mu +a$ and $\Sigma^{\prime }=A\Sigma_{2}A^{⊤}$. It follows that we can reduce the calculation of the Fisher–Rao distance to a canonical case where one argument is $N_{\mathrm{std}}=N\left(0,I\right)$, the standard *d*-variate distribution:

$$\begin{array}{ccc} \rho_{N}\left(N\left(\mu_{1},\Sigma_{1}\right),N\left(\mu_{2},\Sigma_{2}\right)\right) & = & \rho_{N}\left(N_{\mathrm{std}},N\left(\Sigma_{1}^{-\frac{1}{2}}\left(\mu_{2}-\mu_{1}\right),\Sigma_{1}^{-\frac{1}{2}}\Sigma_{2}\Sigma_{1}^{-\frac{1}{2}}\right)\right),\\ & = & \rho_{N}\left(N\left(\Sigma_{2}^{-\frac{1}{2}}\left(\mu_{1}-\mu_{2}\right),\Sigma_{2}^{-\frac{1}{2}}\Sigma_{1}\Sigma_{2}^{-\frac{1}{2}}\right),N_{\mathrm{std}}\right), \end{array}$$

where $\Sigma^{p}$ is the fractional matrix power which can be calculated from the Singular Value Decomposition $ODO^{⊤}$ of Σ (where *O* is an orthogonal matrix and $D=\mathrm{diag}(\lambda_{1},\ldots ,\lambda_{d})$ a diagonal matrix): $\Sigma^{p}=OD^{p}O^{⊤}$ with $D^{p}=\mathrm{diag}\left(\lambda_{1}^{p},\ldots ,\lambda_{d}^{p}\right)$.

The family of normal elliptical distributions can be obtained from the standard normal distribution by the action of the affine group [12,32] Aff(d):

$$N\left(\mu ,\Sigma \right)=\left(\mu ,\Sigma^{\frac{1}{2}}\right).N_{\mathrm{std}}=N\left(\left(\mu ,\Sigma^{\frac{1}{2}}\right).\left(0,I\right)\right).$$

### 1.2. Fisher–Rao Distance between Normal Distributions: Some Subfamilies with Closed-Form Formula

In general, the Fisher–Rao distance $\rho_{N}\left(N_{1},N_{2}\right)$ between two multivariate normal distributions $N_{1}$ and $N_{2}$ is not known in closed form [34,35,36,37], and several lower and upper bounds [38], and numerical techniques such as the geodesic shooting [39,40,41] have been investigated. See [42] for a recent review. Unfortunately, the geodesic shooting (GS) approach is time-consuming and numerically unstable for large Fisher–Rao distances [21,42]. In 3D Diffusion Tensor Imaging (DTI), 3×3 covariance matrices $\Sigma_{i,j,k}$ are stored a 3D grid locations $\mu_{i,j,k}$ thus generating 3D MVNs $N_{i,j,k}=N\left(\mu_{i,j,k},\Sigma_{i,j,k}\right)$ with means $\mu_{i,j,k}$ regularly spaced to each others. The Fisher–Rao distances can be calculated between an MVN $N_{i,j,k}$ and another MVN $N_{i^{\prime },j^{\prime },k^{\prime }}$ in a neighborhood of $N_{i,j,k}$ (using 6- or 26-neighborhood) using geodesic shooting. For larger Fisher–Rao distances between non-neighbors MVNs, we can use the shortest path distance using Dijkstra’s algorithm [43] on the graph induced by the MVNs with edges between adjacent MVNs weighted by their Fisher–Rao distances.

The two main difficulties with calculating the Fisher–Rao distance are

1. to know explicitly the expression of the Riemannian Fisher–Rao geodesic $\gamma_{N}^{\mathrm{FR}}\left(p_{\lambda_{1}},p_{\lambda_{2}};t\right)$ and
2. to integrate, in closed form, the length element $ds_{N}$ along this Riemannian geodesic.

Please note that the Fisher–Rao geodesics [1] $\gamma_{N}^{\mathrm{FR}}\left(p_{\lambda_{1}},p_{\lambda_{2}};t\right)$ are parameterized by constant speed (i.e., $\dot{\mu }\left(t\right)=\dot{\mu }\left(0\right)$ and $\dot{\Sigma }\left(t\right)=\dot{\Sigma }\left(0\right)$), or equivalently parametrized using the arc length:

$$\rho_{N}\left(\gamma_{N}^{\mathrm{FR}}\left(p_{\lambda_{1}},p_{\lambda_{2}};s\right),\gamma_{N}^{\mathrm{FR}}\left(p_{\lambda_{1}},p_{\lambda_{2}};t\right)\right)=\left|s-t\right|\;\rho_{N}\left(p_{\lambda_{1}},p_{\lambda_{2}}\right),\;\forall s,t\in \left[0,1\right].$$

However, in several special cases, the Fisher–Rao distance between normal distributions belonging to restricted subsets of N is known.

Three such prominent cases are (see [42] for other cases)

1. when the normal distributions are univariate (d=1),
2. when we consider the set $N_{\mu }=\left\{N\left(\mu ,\Sigma \right)\;:\;\Sigma \in P\left(d\right)\right\}\subset M_{N}$ of normal distributions sharing the same mean μ (with the embedded submanifold $S_{\mu }\in M$), and
3. when we consider the set $N_{\Sigma }=\left\{N\left(\mu ,\Sigma \right)\;:\;\Sigma \in P\left(d\right)\right\}\subset N$ of normal distributions sharing the same covariance matrix Σ (with the corresponding embedded submanifold $S_{\Sigma }\in M$).

**Figure 1:**
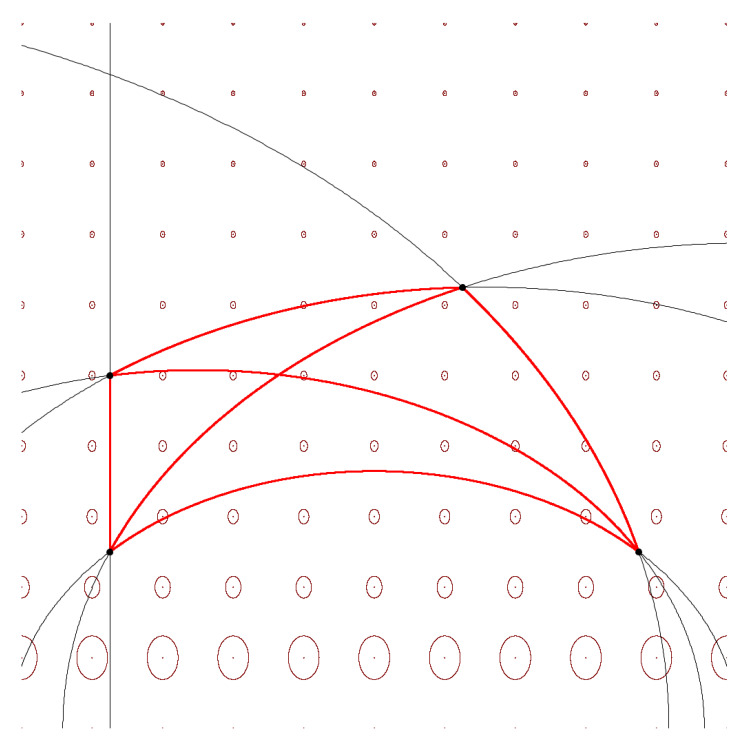
Four univariate normal distributions $N_{1}=N\left(0,1\right)$, $N_{2}=N\left(3,1\right)$, $N_{3}=N\left(2,2.5\right)$, $N_{4}=N\left(0,2\right)$, and their pairwise full geodesics in gray and geodesics linking them in red. The Fisher–Rao distances are $\rho_{N}\left(N_{1},N_{2}\right)=2.6124\ldots$, $\rho_{N}\left(N_{3},N_{4}\right)=0.9317\ldots$, $\rho_{N}\left(N_{1},N_{4}\right)=0.9803\ldots$, $\rho_{N}\left(N_{2},N_{3}\right)=1.4225\ldots$$\rho_{N}\left(N_{2},N_{4}\right)=2.1362\ldots$, and $\rho_{N}\left(N_{1},N_{3}\right)=1.7334\ldots$ The ellipses are Tissot indicatrices, which visualize the metric tensor $g_{N}^{\mathrm{Fisher}}$ at grid positions.

Let us report the formula of the Fisher–Rao distance in these three cases:

- In the univariate case N(1), the Fisher–Rao distance between $N_{1}=N\left(\mu_{1},\sigma_{1}^{2}\right)$ and $N_{2}=N\left(\mu_{2},\sigma_{2}^{2}\right)$ can be derived from the hyperbolic distance [44] expressed in the Poincaré upper space since we have
  $$ds_{N}^{2}=g_{\left(\mu ,\sigma \right)}\left(d\mu ,d\sigma \right)=\frac{d\mu^{2}+2d\sigma^{2}}{\sigma^{2}}=2\frac{{\left(\frac{d\mu }{\sqrt{2}}\right)}^{2}+d\sigma^{2}}{\sigma^{2}}=2\frac{dx^{2}+dy^{2}}{y^{2}}=ds_{\text{Poincaré}}^{2},$$
  where $x=\frac{\mu }{\sqrt{2}}$ and y=σ. It follows that
  $$\rho_{N}\left(N_{1},N_{2}\right)=\sqrt{2}\;\rho_{\text{Poincaré}}\left(\left(x_{1},y_{1}\right),\left(x_{2},y_{2}\right)\right)=\sqrt{2}\;\rho_{\text{Poincaré}}\left(\left(\frac{\mu_{1}}{\sqrt{2}},\sigma_{1}\right),\left(\frac{\mu_{2}}{\sqrt{2}},\sigma_{2}\right)\right).$$
  Thus, we have the following expression for the Fisher–Rao distance between univariate normal distributions:
  (7) $$\rho_{N}\left(N_{1},N_{2}\right)=\sqrt{2}\log\left(\frac{1+\Delta (\mu_{1},\sigma_{1};\mu_{2},\sigma_{2})}{1-\Delta (\mu_{1},\sigma_{1};\mu_{2},\sigma_{2})}\right),$$
  with
  (8) $$\Delta \left(a,b;c,d\right)=\sqrt{\frac{{\left(c-a\right)}^{2}+2{\left(d-b\right)}^{2}}{{\left(c-a\right)}^{2}+2{\left(d+b\right)}^{2}}},\;\left(a,b,c,d\right)\in R^{4}\backslash \left\{0\right\}.$$
  In particular, we have
  – $\Delta \left(a,b;a,d\right)=\left|\frac{d-b}{d+b}\right|$ when a=c (same mean),
  – $\Delta \left(a,b;c,b\right)=\sqrt{\frac{1}{1+8\frac{b^{2}}{{\left(c-a\right)}^{2}}}}$ when b=d (same variance),
  – $\Delta \left(0,1;c,d\right)=\sqrt{\frac{c^{2}+2{\left(d-1\right)}^{2}}{c^{2}+2{\left(d+1\right)}^{2}}}$ when a=0 and b=1 (standard normal).
  In 1D, the affine-invariance property (Property 1) extends to function Δ as follows:
  $$\Delta \left(\mu_{1},\sigma_{1};\mu_{2},\sigma_{2}\right)=\Delta \left(0,1;\frac{\mu_{2}-\mu_{1}}{\sigma_{1}},\frac{\sigma_{2}}{\sigma_{1}}\right)=\Delta \left(\frac{\mu_{1}-\mu_{2}}{\sigma_{2}},\frac{\sigma_{1}}{\sigma_{2}};0,1\right).$$
  Using one of the many identities between inverse hyperbolic functions (e.g., arctanh, arccosh, arcsinh), we can obtain an equivalent formula for Equation (7). For example, since $\mathrm{arctanh}\left(u\right):=\frac{1}{2}\log\left(\frac{1+u}{1-u}\right)$ for 0<u<1, we have equivalently:
  (9) $$\rho_{N}\left(N_{1},N_{2}\right)=2\sqrt{2}\;\mathrm{arctanh}\left(\Delta \left(\mu_{1},\sigma_{1};\mu_{2},\sigma_{2}\right)\right).$$
  The Fisher–Rao geodesics are semi-ellipses with centers located on the *x*-axis. See Appendix A.1 for the parametric equations of Fisher–Rao geodesics between univariate normal distributions. Figure 1 displays four univariate normal distributions with their pairwise geodesics and Fisher–Rao distances.
  Using the identity $\mathrm{arctanh}\left(\frac{u^{2}-1}{u^{2}+1}\right)=\mathrm{arccosh}\left(\frac{1+u^{2}}{2u}\right)$ with $\mathrm{arccosh}\left(x\right):=\log\left(x+\sqrt{x^{2}-1}\right)$, we also have
  $$\rho_{N}\left(N_{1},N_{2}\right)=2\sqrt{2}\;\mathrm{arccosh}\left(\frac{1}{\sqrt{\left(1-\Delta \left(\mu_{1},\sigma_{1};\mu_{2},\sigma_{2}\right)\right)\left(1+\Delta \left(\mu_{1},\sigma_{1};\mu_{2},\sigma_{2}\right)\right)}}\right),$$
  Since the inverse hyperbolic cosecant (CSC) function is defined by arccsch(u):=arccosh(1/u), we further obtain
  $$\rho_{N}\left(N_{1},N_{2}\right)=2\sqrt{2}\;\mathrm{arccsch}\left(\sqrt{\left(1-\Delta \left(\mu_{1},\sigma_{1};\mu_{2},\sigma_{2}\right)\right)\left(1+\Delta \left(\mu_{1},\sigma_{1};\mu_{2},\sigma_{2}\right)\right)}\right),$$
  We can also write
  $$\rho_{N}\left(N_{1},N_{2}\right)=\sqrt{2}\;\mathrm{arccosh}\left(1+\frac{{\left(\mu_{2}-\mu_{1}\right)}^{2}+2{\left(\sigma_{2}-\sigma_{1}\right)}^{2}}{4\sigma_{1}\sigma_{2}}\right)$$
  Thus, using the many-conversions formula between inverse hyperbolic functions, we obtain many equivalent different formulas of the Fisher–Rao distance, which are used in the literature.
- In the second case, the Fisher–Rao distance between $N_{1}=N\left(\mu ,\Sigma_{1}\right)$ and $N_{2}=N\left(\mu ,\Sigma_{2}\right)$ has been reported in [6,7,45,46,47]:
  (10) $$\begin{array}{ccc} \rho_{N_{\mu }}\left(N_{1},N_{2}\right) & = & \sqrt{\frac{1}{2}\sum_{i=1}^{d}\log^{2}\lambda_{i}\left(\Sigma_{1}^{-1}\Sigma_{2}\right)}, \end{array}$$
  (11) $$\begin{array}{ccc} & = & \rho_{N_{\mu }}\left(\Sigma_{1},\Sigma_{2}\right), \end{array}$$
  where $\lambda_{i}\left(M\right)$ denotes the *i*-th generalized largest eigenvalue of matrix *M*, where the generalized eigenvalues are solutions of the equation $|\Sigma_{1}-\lambda \Sigma_{2}|=0$. Let us notice that $\rho_{N_{\mu }}\left(\left(\mu ,\Sigma_{1}\right),\left(\mu ,\Sigma_{2}\right)\right)=\rho_{N_{\mu }}\left(\left(\mu ,\Sigma_{1}^{-1}\right),\left(\mu ,\Sigma_{2}^{-1}\right)\right)$ since $\lambda_{i}\left(\Sigma_{2}^{-1}\Sigma_{1}\right)=\frac{1}{\lambda_{i}\left(\Sigma_{1}^{-1}\Sigma_{2}\right)}$ and $\log^{2}\lambda_{i}\left(\Sigma_{2}^{-1}\Sigma_{1}\right)={\left(-\log\lambda_{i}\left(\Sigma_{1}^{-1}\Sigma_{2}\right)\right)}^{2}=\log^{2}\lambda_{i}\left(\Sigma_{1}^{-1}\Sigma_{2}\right)$. Matrix $\Sigma_{1}^{-1}\Sigma_{2}$ may not be SPD and thus the $\lambda_{i}$’s are generalized eigenvalues. We may consider instead the SPD matrix $\Sigma_{1}^{-\frac{1}{2}}\Sigma_{2}\Sigma_{1}^{-\frac{1}{2}}$ which is SPD and such that $\lambda_{i}\left(\Sigma_{1}^{-1}\Sigma_{2}\right)=\lambda_{i}\left(\Sigma_{1}^{-\frac{1}{2}}\Sigma_{2}\Sigma_{1}^{-\frac{1}{2}}\right)$. The Fisher–Rao distance of Equation (11) can be equivalently written [48] as
  $$\rho_{N_{\mu }}\left(N_{1},N_{2}\right)=\frac{1}{\sqrt{2}}\;{∥\mathrm{Log}\left(\Sigma_{1}^{-\frac{1}{2}}\Sigma_{2}\Sigma_{1}^{-\frac{1}{2}}\right)∥}_{F},$$
  where Log(M) is the matrix logarithm (unique when *M* is SPD) and ${∥M∥}_{F}=\sqrt{\sum_{i,j}M_{i,j}^{2}}=\sqrt{\mathrm{tr}(MM^{⊤})}$ is the matrix Fröbenius norm. This metric distance between SPD matrices although first studied by Siegel [45] in 1964 was rediscovered and analyzed recently in [49] (2003). Let $\rho_{\mathrm{SPD}}\left(P_{1},P_{2}\right)=\sqrt{\sum_{i=1}^{d}\log^{2}\lambda_{i}\left(P_{1}^{-1}P_{2}\right)}$ so that $\rho_{N_{\mu }}\left(N\left(\mu ,P_{1}\right),N\left(\mu ,P_{2}\right)\right)=\frac{1}{\sqrt{2}}\;\rho_{\mathrm{SPD}}\left(P_{1},P_{2}\right)$.
  The Riemannian SPD distance $\rho_{\mathrm{SPD}}$ enjoys the following well-known invariance properties:
  – Invariance by congruence transformation:
    (12) $$\forall X\in \mathrm{GL}\left(d\right),\;\rho_{\mathrm{SPD}}\left(XP_{1}X^{⊤},XP_{2}X^{⊤}\right)=\rho_{\mathrm{SPD}}\left(P_{1},P_{2}\right),$$
  – Invariance by inversion:
    $$\forall P_{1},P_{2}\in P\left(d\right),\;\rho \left(P_{1}^{-1},P_{2}^{-1}\right)=\rho_{\mathrm{SPD}}\left(P_{1},P_{2}\right).$$
    Let $P_{1}=L_{1}L_{1}^{⊤}$ be the Cholesky decomposition (unique when $P_{1}≻0$). Then apply the congruence invariance for $X=L_{1}^{-1}$:
    (13) $$\rho_{\mathrm{SPD}}\left(P_{1},P_{2}\right)=\rho_{\mathrm{SPD}}\left(L_{1}^{-1}P_{1}{\left(L_{1}^{-1}\right)}^{⊤},L_{1}^{-1}P_{2}{\left(L_{1}^{-1}\right)}^{⊤}\right)=\rho_{\mathrm{SPD}}\left(I,L_{1}^{-1}P_{2}{\left(L_{1}^{-1}\right)}^{⊤}\right).$$
    We can also consider the factorization $P_{1}=S_{1}S_{1}$ where $S_{1}=P_{1}^{\frac{1}{2}}$ is the unique symmetric square root matrix [50]. Then we have
    $$\rho_{\mathrm{SPD}}\left(P_{1},P_{2}\right)=\rho_{\mathrm{SPD}}\left(S_{1}^{-1}P_{1}{\left(S_{1}^{-1}\right)}^{⊤},S_{1}^{-1}P_{2}{\left(S_{1}^{-1}\right)}^{⊤}\right)=\rho_{\mathrm{SPD}}\left(I,S_{1}^{-1}P_{2}{\left(S_{1}^{-1}\right)}^{⊤}\right).$$
- The Fisher–Rao distance between $N_{1}=N\left(\mu_{1},\Sigma \right)$ and $N_{2}=N\left(\mu_{2},\Sigma \right)$ has been reported in closed form [42] (Proposition 3). The method is described with full details in Appendix B. We present a simpler scheme based on the inverse $\Sigma^{-\frac{1}{2}}$ of the symmetric square root factorization [50] of $\Sigma =\Sigma^{\frac{1}{2}}\Sigma^{\frac{1}{2}}$ (ith ${\left(\Sigma^{-\frac{1}{2}}\right)}^{⊤}=\Sigma^{-\frac{1}{2}}$). Let us use the affine-invariance property of the Fisher–Rao distance under the affine transformation $\Sigma^{-\frac{1}{2}}$ and then apply affine invariance under translation as follows:
  $$\begin{array}{ccc} \rho_{N}\left(N\left(\mu_{1},\Sigma \right),N\left(\mu_{2},\Sigma \right)\right) & = & \rho_{N}\left(N\left(\Sigma^{-\frac{1}{2}}\mu_{1},\Sigma^{-\frac{1}{2}}\Sigma \Sigma^{-\frac{1}{2}}\right),N\left(\Sigma^{-\frac{1}{2}}\mu_{2},\Sigma^{-\frac{1}{2}}\Sigma \Sigma^{-\frac{1}{2}}\right)\right),\\ & = & \rho_{N}\left(N\left(0,I\right),N\left(\Sigma^{-\frac{1}{2}}\left(\mu_{2}-\mu_{1}\right),I\right)\right),\\ & = & \rho_{N}(N\left(0,1\right),N(∥\Sigma^{-\frac{1}{2}}\left(\mu_{2}-\mu_{1}\right){∥}_{2},1)). \end{array}$$
  The right-hand side Fisher–Rao distance is computed from Equation (7) and justified by the method [42] (Proposition 3) described in Appendix B using a rotation matrix *R* with $RR^{⊤}=I$ so that
  $$\begin{array}{ccc} \rho_{N}\left(N\left(0,I\right),N\left(\Sigma^{-\frac{1}{2}}\left(\mu_{2}-\mu_{1}\right),I\right)\right) & = & \rho_{N}\left(N\left(0,I\right),N\left(R\Sigma^{-\frac{1}{2}}\left(\mu_{2}-\mu_{1}\right),RIR^{⊤}\right)\right),\\ & = & \rho_{N}(N\left(0,I\right),∥\Sigma^{-\frac{1}{2}}\left(\mu_{2}-\mu_{1}\right){∥}_{2},I)). \end{array}$$
  Then we apply the formula of Equation (23) of [42]. Section 1.5 shall report a simpler closed-form formula by proving that the Fisher–Rao distance between $N(\mu_{1},\Sigma )$ and $N(\mu_{2},\Sigma )$ is a scalar function of their Mahalanobis distance [51] using the algebraic method of maximal invariants [52].

### 1.3. Fisher–Rao Distance: Totally versus Non-Totally Geodesic Submanifolds

Consider $N^{\prime }=\left\{N\left(\lambda \right)\;:\;\lambda^{\prime }\in \Lambda^{\prime }\right\}\subset N$ a statistical submodel of the MVN statistical model N. Using the Fisher information matrix $I_{\lambda^{\prime }}\left(\lambda^{\prime }\right)$, we obtain the intrinsic Fisher–Rao manifold $M^{\prime }=M_{N^{\prime }}$. We may also consider $M^{\prime }$ to be an embedded submanifold of M. Let us write $S^{\prime }=S_{N^{\prime }}\subset M$ the embedded submanifold.

A totally geodesic submanifold $S^{\prime }\subset M$ is such that the geodesics $\gamma_{M^{\prime }}\left(N_{1}^{\prime },N_{2}^{\prime };t\right)$ fully stay in $M^{\prime }$ for any pair of points $N_{1}^{\prime },N_{2}^{\prime }\in N^{\prime }$. For example, the submanifold $M_{\mu }=\left\{N\left(\mu ,\Sigma \right)\;:\;\Sigma \in P\left(d\right)\right\}\subset M$ of MVNs with fixed mean μ is a totally geodesic submanifold [53] of M but the submanifold $M_{\Sigma }=\left\{N\left(\mu ,\Sigma \right)\;:\;\mu \in R^{d}\right\}\subset M$ of MVNs sharing the same covariance matrix Σ is not totally geodesic. When an embedded submanifold S⊂M is totally geodesic, we always have $\rho_{M}\left(N_{1},N_{2}\right)=\rho_{S}\left(N_{1},N_{2}\right)$. Thus, we have $\rho_{N}\left(N\left(\mu ,\Sigma_{1}\right),N\left(\mu ,\Sigma_{2}\right)\right)=\rho_{\mathrm{SPD}}\left(\Sigma_{1},\Sigma_{2}\right)$. However, when an embedded submanifold S⊂M is not totally geodesic, we have $\rho_{M}\left(N_{1},N_{2}\right)\leq \rho_{S}\left(N_{1},N_{2}\right)$ because the Riemannian geodesic length in S is necessarily longer or equal than the Riemannian geodesic length in M. The merit to consider submanifolds is to be able to calculate in closed form the Fisher–Rao distance which may then provide an upper bound on the Fisher–Rao distance for the full statistical model. For example, consider $N_{1}=N\left(\mu_{1},\Sigma \right)$ and $N_{2}=N\left(\mu_{2},\Sigma \right)$ in $M_{\Sigma }$, a non-totally geodesic submanifold. The Rao distance between $N_{1}$ and $N_{2}$ in M is upper bounded by the Riemannian distance in $M_{\Sigma }$ (with line element $ds_{\Sigma }^{2}=d\mu^{⊤}\Sigma^{-1}d\mu$) which corresponds to the Mahalanobis distance [10,51]$\Delta_{\Sigma }\left(\mu_{1},\mu_{2}\right)$:

(14) $$\rho_{M_{\mu }}\left(N_{1},N_{2}\right)\leq \Delta_{\Sigma }\left(\mu_{1},\mu_{2}\right):=\sqrt{{\left(\mu_{2}-\mu_{1}\right)}^{⊤}\Sigma^{-1}\left(\mu_{2}-\mu_{1}\right)}.$$

The Mahalanobis distance can be interpreted as the Euclidean distance $D_{E}\left(p,q\right)=\Delta_{I}\left(p,q\right)=\sqrt{{\left(p-q\right)}^{⊤}\left(p-q\right)}$ (where *I* denotes the identity matrix) after an affine transformation: Let $\Sigma =LL^{⊤}=U^{⊤}U$ be the Cholesky decomposition of Σ≫0 with *L* a lower triangular matrix or $U=L^{⊤}$ an upper triangular matrix. Then we have

$$\begin{array}{ccc} \Delta_{\Sigma }\left(\mu_{1},\mu_{2}\right) & = & \sqrt{{\left(\mu_{2}-\mu_{1}\right)}^{⊤}{\left(L^{⊤}\right)}^{-1}L^{-1}\left(\mu_{2}-\mu_{1}\right)},\\ & = & ∥\Sigma^{-\frac{1}{2}}\left(\mu_{2}-\mu_{1}\right){∥}_{2},\\ & = & \Delta_{I}\left(L^{-1}\mu_{1},L^{-1}\mu_{2}\right)=D_{E}\left(L^{-1}\mu_{1},L^{-1}\mu_{2}\right), \end{array}$$

where ${∥\cdot ∥}_{2}$ denotes the vector $\ell_{2}$-norm.

The Rao distance $\rho_{\Sigma }$ of Equation (A1) between two MVNs with fixed covariance matrix emanates from the property that the submanifold $M_{[v],\Sigma }=\left\{N\left(av,\Sigma \right)\;:\;a\in R\right\}$ is totally geodesic [54].

Let us emphasize that for a submanifold S⊂M to be totally geodesic or not depend on the underlying metric in M. The same subset $N^{\prime }\subset N$ with N equipped with two different metrics $g_{1}$ and $g_{2}$ can be totally geodesic regarding $g_{1}$ and non-totally geodesic regarding $g_{2}$. See Remark 3 for such an example.

**Figure 2:**
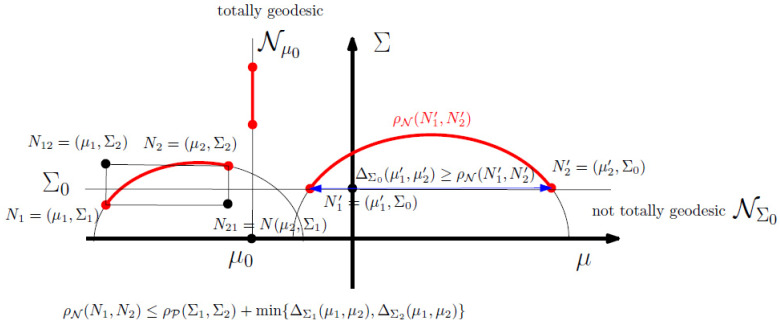
The submanifolds $N_{\Sigma }$ are not totally geodesic (i.e., $\rho_{N}\left(N_{1}^{\prime },N_{2}^{\prime }\right)$ is upper bounded by their Mahalanobis distance) but the submanifolds $N_{\mu }$ are totally geodesic. Using the triangle inequality of the Riemannian metric distance $\rho_{N}$, we can upper bound $\rho_{N}\left(N_{1},N_{2}\right)$.

In general, using the triangle inequality of the Riemannian metric distance $\rho_{N}$, we can upper bound $\rho_{N}\left(N_{1},N_{2}\right)$ with $N_{1}=\left(\mu_{1},\Sigma_{1}\right)$ and $N_{1}=\left(\mu_{2},\Sigma_{2}\right)$ as follows:

$$\begin{array}{ccc} \rho_{N}\left(N_{1},N_{2}\right) & \leq & \rho_{M_{\mu_{1}}}\left(N_{1},N_{12}\right)+\rho_{M_{\Sigma_{2}}}\left(N_{12},N_{2}\right),\\ & \leq & \rho_{M_{\Sigma_{1}}}\left(N_{1},N_{21}\right)+\rho_{M_{\mu_{2}}}\left(N_{21},N_{2}\right), \end{array}$$

where $N_{12}=\left(\mu_{1},\Sigma_{2}\right)$ and $N_{21}=N\left(\mu_{2},\Sigma_{1}\right)$. See Figure 2 for an illustration of the Fisher–Rao geodesic triangle $▵N_{1},N_{2},N_{12}$. Furthermore, since $\rho_{N_{\Sigma_{1}}}\left(N_{1},N_{21}\right)\leq \Delta_{\Sigma_{1}}\left(\mu_{1},\mu_{2}\right)$ and $\rho_{N_{\Sigma_{2}}}\left(N_{12},N_{2}\right)\leq \Delta_{\Sigma_{2}}\left(\mu_{1},\mu_{2}\right)$, we obtain the following upper bound on the Rao distance between MVNs:

(15) $$\rho_{N}\left(N_{1},N_{2}\right)\leq \rho_{P}\left(\Sigma_{1},\Sigma_{2}\right)+\min\left\{\Delta_{\Sigma_{1}}\left(\mu_{1},\mu_{2}\right),\Delta_{\Sigma_{2}}\left(\mu_{1},\mu_{2}\right)\right\}.$$

See also [55].

In general, the difficulty with calculating the Fisher–Rao distance comes from the fact that

1. we do not know the Fisher–Rao geodesics with boundary value conditions (BVP) in closed form but the geodesics with initial value conditions [48] (IVP) are known explicitly using the natural parameters $\left(\Sigma^{-1}\mu ,\Sigma^{-1}\right)$ of MVNs,
2. we must integrate the line element $ds_{N}$ along the geodesic.

As we shall see in Section 3.1, the above first problem is much harder to solve than the second problem which can be easily approximated by discretizing the curve. The lack of a closed-form formula and fast and good approximations for $\rho_{N}$ between MVNs is a current limiting factor for its use in applications. Indeed, many applications (e.g., [56,57]) consider the restricted case of the Rao distance between zero-centered MVNs which have closed form (distance of Equation (11) in the SPD cone). The SPD cone is a symmetric Hadamard manifold, and its isometries have been fully studied and classified in [58] (Section 4). The Fisher–Rao geometry of zero-centered generalized MVNs was recently studied in [59].

### 1.4. Contributions and Paper Outline

The main contribution of this paper is to propose an approximation of $\rho_{N}$ based on Calvo and Oller’s embedding [19] (C&O for short) and report its experimental performance. First, we concisely recall C&O’s family of embeddings $f_{\beta }$ of N(d) as submanifolds ${\bar{N}}_{\beta }$ of P(d+1) in Section 2. Next, we present our approximation technique in Section 3 which differs from the usual geodesic shooting approach [39], and report experimental results. Finally, we study some information–geometric properties [1] of the isometric embedding in Section 5 such as the fact that it preserves mixture geodesics (embedded C&O submanifold is autoparallel with respect to the mixture affine connection) but not exponential geodesics. Moreover, we prove that the Fisher–Rao distance between multivariate normal distributions sharing the same covariance matrix is a scalar function of their Mahalanobis distance in Section 1.5 using the framework of Eaton [52] of maximal invariants.

### 1.5. A Closed-Form Formula for the Fisher–Rao Distance between Normal Distributions Sharing the Same Covariance Matrix

Consider the Fisher–Rao distance between $N_{1}=\left(\mu_{1},\Sigma \right)$ and $N_{1}=\left(\mu_{2},\Sigma \right)$ for a fixed covariance matrix Σ and the translation action a.μ:=μ+a of the translation group $R^{d}$ (a subgroup of the affine group). Both the Fisher–Rao distance and the Mahalanobis distance are invariant under translations:

$$\rho_{N}\left(\left(\mu_{1}+a,\Sigma \right),\left(\mu_{2}+a,\Sigma \right)\right)=\rho_{N}\left(\left(\mu_{1},\Sigma \right),\left(\mu_{2},\Sigma \right)\right),\;\Delta_{\Sigma }\left(\mu_{1}+a,\mu_{2}+a\right)=\Delta_{\Sigma }\left(\mu_{1},\mu_{2}\right).$$

To prove that $\rho_{N}\left(\left(\mu_{1},\Sigma \right),\left(\mu_{2},\Sigma \right)\right)=h_{\mathrm{FR}}\left(\Delta_{\Sigma }\left(\mu_{1},\mu_{2}\right)\right)$ for a scalar function $h_{\mathrm{FR}}\left(\cdot \right)$, we shall prove that the Mahalanobis distance is a maximal invariant, and use the framework of maximal invariants of Eaton [52] (Chapter 2) who proved that any other invariant function is necessarily a function of a maximal invariant, i.e., a function of the Mahalanobis distance in our case.

The Mahalanobis distance is a maximal invariant because we can write $\Delta_{\Sigma }\left(\mu_{1},\mu_{2}\right)=\Delta_{1}\left(0,\Delta_{\Sigma }\left(\mu_{1},\mu_{2}\right)\right)$ and when $\Delta_{\Sigma }\left(\mu_{1},\mu_{2}\right)=\Delta_{\Sigma }\left(\mu_{1}^{\prime },\mu_{2}^{\prime }\right)$ in 1D there exists a∈R such that $\left(\mu_{1}+a,\mu_{2}+a\right)=\left(\mu_{1}^{\prime },\mu_{2}^{\prime }\right)$. We must prove equivalently that when $|m_{1}-m_{2}\left|=\right|m_{1}^{\prime }-m_{2}^{\prime }|$ that there exists a∈R such that $\left(m_{1}+a,m_{2}+a\right)=\left(m_{1}^{\prime },m_{2}^{\prime }\right)$. Assume without loss of generality that $m_{1}\geq m_{2}$. When $m_{1}-m_{2}=m_{1}^{\prime }-m_{2}^{\prime }$, there exists $a=m_{1}^{\prime }-m_{1}$ so that $m_{1}^{\prime }=a.m_{1}=m_{1}+a$ and $m_{2}^{\prime }=a.m_{2}=m_{2}+a$ with $m_{1}^{\prime }-m_{2}^{\prime }=m_{1}-m_{2}$. Thus, using Eaton’s theorem [52], there exists a scalar function $h_{\mathrm{FR}}$ such that $\rho_{N}\left(\left(\mu_{1},\Sigma \right),\left(\mu_{2},\Sigma \right)\right)=h_{\mathrm{FR}}\left(\Delta_{\Sigma }\left(\mu_{1},\mu_{2}\right)\right)$.

To find explicitly the scalar function $h_{\mathrm{FR}}\left(\cdot \right)$, let us consider the univariate case of normal distributions for which the Fisher–Rao distance is given in closed form in Equation (7). In that case, the univariate Mahalanobis distance is $\Delta_{\sigma^{2}}\left(\mu_{1},\mu_{2}\right)=\sqrt{\left(\mu_{2}-\mu_{1}\right){\left(\sigma^{2}\right)}^{-1}\left(\mu_{2}-\mu_{1}\right)}=\frac{|\mu_{2}-\mu_{1}|}{\sigma }$ and we can write formula of Equation (7) as $h_{\mathrm{FR}}\left(\Delta_{\sigma^{2}}\left(\mu_{1},\mu_{2}\right)\right)$ with

(16) $$\begin{array}{ccc} h_{\mathrm{FR}}\left(u\right) & = & \sqrt{2}\;\log\left(\frac{\sqrt{8+u^{2}}+u}{\sqrt{8+u^{2}}-u}\right), \end{array}$$

(17) $$\begin{array}{ccc} & = & \sqrt{2}\;\mathrm{arccosh}\left(1+\frac{1}{4}u^{2}\right), \end{array}$$

using the identities

$$\log\left(x\right)=\mathrm{arccosh}\left(\frac{1+x^{2}}{2x}\right)=\mathrm{arctanh}\left(\frac{x^{2}-1}{1+x^{2}}\right),\;x>1,$$

where $\mathrm{arctanh}\left(u\right)=\frac{1}{2}\log\frac{1+u}{1-u}$.

**Proposition 1.**  *The Fisher–Rao distance $\rho_{N}\left(\left(\mu_{1},\Sigma \right),\left(\mu_{2},\Sigma \right)\right)$ between two MVNs with same covariance matrix is*

(18) $$\begin{array}{ccc} \rho_{N}\left(\left(\mu_{1},\Sigma \right),\left(\mu_{2},\Sigma \right)\right) & = & \rho_{N}\left(\left(0,1\right),\left(\Delta_{\Sigma }\left(\mu_{1},\mu_{2}\right),1\right)\right), \end{array}$$

(19) $$\begin{array}{ccc} & \;\;\;\;\;\;\;\;\;\;\;\;\;\;\;\;\;\;\;\;\;\;\;\;\;\;\;\;\;\;\;\;\;\;\;\;\;\;\;\;\;\;\;\;\;\;\;\;\;\;\;\;\;\;\;\;\;\;\;\;\;\;\;= & \sqrt{2}\;\log\left(\frac{\sqrt{8+\Delta_{\Sigma }^{2}\left(\mu_{1},\mu_{2}\right)}+\Delta_{\Sigma }\left(\mu_{1},\mu_{2}\right)}{\sqrt{8+\Delta_{\Sigma }^{2}\left(\mu_{1},\mu_{2}\right)}-\Delta_{\Sigma }\left(\mu_{1},\mu_{2}\right)}\right), \end{array}$$

(20) $$\begin{array}{ccc} & \;\;\;\;\;\;\;\;\;\;\;\;\;\;\;\;\;\;\;\;\;\;\;\;\;\;\;\;\;\;\;\;\;\;\;\;\;\;\;\;\;= & \sqrt{2}\;\mathrm{arccosh}\left(1+\frac{1}{4}\Delta_{\Sigma }^{2}\left(\mu_{1},\mu_{2}\right)\right), \end{array}$$

*where $\Delta_{\Sigma }\left(\mu_{1},\mu_{2}\right)=\sqrt{{\left(\mu_{2}-\mu_{1}\right)}^{⊤}\Sigma^{-1}\left(\mu_{2}-\mu_{1}\right)}$ is the Mahalanobis distance.*

Indeed, notice that the *d*-variate Mahalanobis distance $\Delta_{\Sigma }\left(\mu_{1},\mu_{2}\right)$ can be interpreted as a univariate Mahalanobis distance between the standard normal distribution N(0,1) and $N(\Delta_{\Sigma }\left(\mu_{1},\mu_{2}\right),1)$:

$$\Delta_{\Sigma }\left(\mu_{1},\mu_{2}\right)=\Delta_{1}\left(0,\Delta_{\Sigma }\left(\mu_{1},\mu_{2}\right)\right).$$

Thus, we have $\rho_{N}\left(\left(\mu_{1},\Sigma \right),\left(\mu_{2},\Sigma \right)\right)=\rho_{N}\left(\left(0,1\right),\left(\Delta_{\Sigma }\left(\mu_{1},\mu_{2}\right),1\right)\right)$, where the right-hand-side term is the univariate Fisher–Rao distance of Equation (7). Let us notice that the square length element on $M_{\Sigma }$ is $ds^{2}=d\mu^{⊤}\Sigma^{-1}d\mu =\Delta_{\Sigma }^{2}\left(\mu ,\mu +d\mu \right)$. This result can be extended to elliptical distributions [12] (Theorem 1).

Let us corroborate this result by checking the formula of Equation (1) with two examples in the literature: In [38] (Figure 4), we Fisher–Rao distance between $N_{1}=\left(0,I\right)$ and $N_{2}=\left(\left[\begin{array}{c} \frac{1}{2}\\ \frac{1}{2} \end{array}\right],I\right)$ is studied. We find $\rho_{N}\left(N_{1},N_{2}\right)=0.69994085$ in accordance with their result shown in Figure 4. The second example is Example 1 of [42] (p. 11) with $N_{1}=\left(\left[\begin{array}{c} -1\\ 0 \end{array}\right],\Sigma \right)$ and $N_{2}=\left(\left[\begin{array}{c} 6\\ 3 \end{array}\right],\Sigma \right)$ for $\Sigma =\left[\begin{array}{cc} 1.1 & 0.9\\ 0.9 & 1.1 \end{array}\right]$. Formula of Equation (18) yields the Fisher–Rao distance 5.006483034546878 in accordance with [42] which reports 5.00648.

Similarly, the statistical Ali–Silvey–Csiszár *f*-divergences [60,61]

$$I_{f}\left[p_{\left(\mu_{1},\Sigma \right)}:p_{\left(\mu_{2},\Sigma \right)}\right]=\int_{R^{d}}p_{\left(\mu_{1},\Sigma \right)}\left(x\right)\;f\left(\frac{p_{\left(\mu_{2},\Sigma \right)}}{p_{\left(\mu_{1},\Sigma \right)}}\right)dx,$$

between two MVNs sharing the same covariance matrix are increasing functions of the Mahalanobis distance because the *f*-divergences between two MVNs sharing the same covariance matrix are invariant under the action of the translation group [62]. Thus, we have $I_{f}\left[p_{\left(\mu_{1},\Sigma \right)}:p_{\left(\mu_{2},\Sigma \right)}\right]=h_{f}\left(\Delta_{\Sigma }\left(\mu_{1},\mu_{2}\right)\right)$. Since $\Delta_{\Sigma }\left(\mu_{1},\mu_{2}\right)=\Delta_{1}\left(0,\Delta_{\Sigma }\left(\mu_{1},\mu_{2}\right)\right)$, we thus have

$$I_{f}\left[p_{\left(\mu_{1},\Sigma \right)}:p_{\left(\mu_{2},\Sigma \right)}\right]=h_{f}(\Delta_{1}\left(0,\Delta_{\Sigma }\left(\mu_{1},\mu_{2}\right)\right)=I_{f}\left[p_{\left(0,1\right)}:p_{\left(\Delta_{\Sigma }\left(\mu_{1},\mu_{2}\right),1\right)}\right],$$

where the right-hand side *f*-divergence is between univariate normal distributions. See Table 2 of [62] for some explicit functions $h_{f}$.

## 2. Calvo and Oller’s Family of Diffeomorphic Embeddings

Calvo and Oller [19,32] noticed that we can embed the space of normal distributions in P(d+1) by using the following mapping:

(21) $$f_{\beta }\left(N\right)=f_{\beta }\left(\mu ,\Sigma \right)=\left[\begin{array}{cc} \Sigma +\beta \mu \mu^{⊤} & \beta \mu \\ \beta \mu^{⊤} & \beta \end{array}\right]\in P\left(d+1\right),$$

where $\beta \in R_{>0}$ and N=N(μ,Σ). Notice that since the dimension of P(d+1) is $\frac{\left(d+1)(d+2\right)}{2}$, we only use $\frac{\left(d+1)(d+2\right)}{2}-\frac{d(d+3)}{2}=1$ extra dimension for embedding N(d) into P(d+1). By foliating $P=R_{>0}\times P_{c}$ where $P_{c}=\left\{P\in P\;:\;|P|=c\right\}$ denotes the subsets of P with determinant *c*, we obtain the following Riemannian Calvo and Oller metric on the SPD cone:

$$\begin{array}{ccc} ds_{\mathrm{CO}}^{2} & = & \frac{1}{2}\mathrm{tr}\left({\left(f^{-1}\left(\mu ,\Sigma \right)df\left(\mu ,\Sigma \right)\right)}^{2}\right),\\ & = & \frac{1}{2}{\left(\frac{d\beta }{\beta }\right)}^{2}+\beta d\mu^{⊤}\Sigma^{-1}d\mu +\frac{1}{2}\mathrm{tr}\left({\left(\Sigma^{-1}d\Sigma \right)}^{2}\right). \end{array}$$

Let

$${\bar{N}}_{\beta }\left(d\right)=\left\{\bar{P}=f_{\beta }\left(\mu ,\Sigma \right)\;:\;\left(\mu ,\Sigma \right)\in N\left(d\right)=R^{d}\times P\left(d\right)\right\}$$

denote the submanifold of P(d+1) of codimension 1, and $\bar{N}={\bar{N}}_{1}$ (i.e., β=1). The family of mappings $f_{\beta }$ provides diffeomorphisms between N(d) and ${\bar{N}}_{\beta }\left(d\right)$. Let $f_{\beta }^{-1}\left(\bar{P}\right)=\left(\mu_{\bar{P}},\Sigma_{\bar{P}}\right)$ denote the inverse mapping for $\bar{P}\in {\bar{N}}_{\beta }\left(d\right)$, and let $f=f_{1}$ (i.e., β=1):

$$f\left(N\right)=f\left(\mu ,\Sigma \right)=\left[\begin{array}{cc} \Sigma +\mu \mu^{⊤} & \mu \\ \mu^{⊤} & 1 \end{array}\right].$$

By equipping the cone P(d+1) by the trace metric [63,64] (also called the affine invariant Riemannian metric, AIRM) scaled by $\frac{1}{2}$:

$$g_{P}^{\mathrm{trace}}\left(P_{1},P_{2}\right):=\mathrm{tr}\left(P^{-1}P_{1}P^{-1}P_{2}\right)$$

(yielding the squared line element $ds_{P}^{2}=\frac{1}{2}\mathrm{tr}\left({\left(P\;dP\right)}^{2}\right)$), Calvo and Oller [19] proved that $\bar{N}\left(d\right)$ is isometric to N(d) (i.e., the Riemannian metric of P(d+1) restricted to N(d) coincides with the Riemannian metric of N(d) induced by *f*) but $\bar{N}\left(d\right)$ is not totally geodesic (i.e., the geodesics $\gamma_{P}\left({\bar{P}}_{1},{\bar{P}}_{2};t\right)$ for ${\bar{P}}_{1}=f\left(N_{1}\right),{\bar{P}}_{2}=f\left(N_{2}\right)\in \bar{N}\left(d\right)$ leaves the embedded normal submanifold $\bar{N}\left(d\right))$. Please note that $g_{P}^{\mathrm{trace}}$ can be interpreted as the Fisher metric for the family $N_{0}$ of 0-centered normal distributions. Thus, we have $\left(N\left(d\right),g^{\mathrm{Fisher}}\right)↪\left(P\left(d+1\right),g^{\mathrm{trace}}\right)$, and the following diagram between parameter spaces and corresponding distributions:

$$\begin{array}{ccc} N(d) & ↪ & N_{0}\left(d+1\right)\\ ↕ & & ↕\\ \Lambda (d) & ↪ & P(d+1) \end{array}$$

**Remark 2.**  *The trace metric was first studied by Siegel [45,65] using the wider scope of complex symmetric matrices with positive–definite imaginary parts generalizing the Poincaré upper half-plane (see Appendix D).*

We omit to specify the dimensions and write for short N, $\bar{N}$, and P when clear from the context. Thus, C&O proposed to use the embedding $f=f_{1}$ to give a lower bound $\rho_{\mathrm{CO}}$ of the Fisher–Rao distance $\rho_{N}$ between normals:

(22) $${\mathrm{LC}}_{\mathrm{CO}}:\;\rho_{N}\left(N_{1},N_{2}\right)\geq \rho_{\mathrm{CO}}\left(\underset{{\bar{P}}_{1}}{\underbrace{f(\mu_{1},\Sigma_{1})}},\underset{{\bar{P}}_{2}}{\underbrace{f(\mu_{2},\Sigma_{2})}}\right)=\sqrt{\frac{1}{2}\sum_{i=1}^{d+1}\log^{2}\lambda_{i}\left({\bar{P}}_{1}^{-1}{\bar{P}}_{2}\right)}.$$

We let $\rho_{\mathrm{CO}}\left(N_{1},N_{2}\right)=\rho_{\mathrm{CO}}\left(f\left(N_{1}\right),f\left(N_{2}\right)\right)$. The $\rho_{\mathrm{CO}}$ distance is invariant under affine transformations such as the Fisher–Rao distance of Property 1:

**Property 2**  (affine invariance of C&O distance [19])**.** *For all $A\in \mathrm{GL}\left(d\right),a\in R^{d}$, we have $\rho_{\mathrm{CO}}\left(\left(A\mu_{1}+a,A\Sigma_{1}A^{⊤}\right),\left(A\mu_{2}+a,A\Sigma_{2}A^{⊤}\right)\right)=\rho_{\mathrm{CO}}\left(N\left(\mu_{1},\Sigma_{1}\right),N\left(\mu_{2},\Sigma_{2}\right)\right)$.*

**Figure 3:**
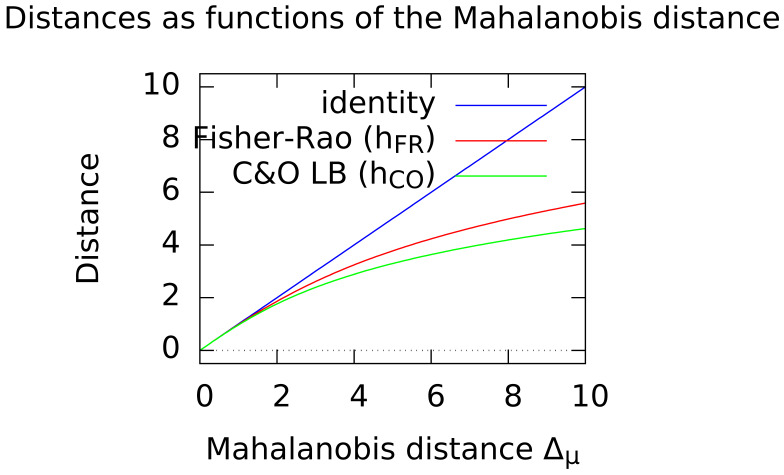
Quality of the C&O lower bound compared to the exact Fisher–Rao distance in the case of $N_{1},N_{2}\in M_{\Sigma }$ (MVNs sharing the same covariance matrix Σ). We have $\rho_{\mathrm{CO}}\leq \rho_{N}\leq \Delta_{\Sigma }$.

When $\Sigma_{1}=\Sigma_{2}=\Sigma$, we have $|{\bar{P}}_{1}\left|=\right|{\bar{P}}_{2}\left|=|\Sigma \right|$. Since the Riemannian geodesics $\gamma_{P}\left(P_{1},P_{2};t\right)$ in the SPD cone are given by $\gamma_{P}\left(P_{1},P_{2};t\right)=P_{1}^{\frac{1}{2}}{\left(P_{1}^{-\frac{1}{2}}P_{2}P_{1}^{-\frac{1}{2}}\right)}^{t}P_{1}^{\frac{1}{2}}$ [66] (also written $\gamma_{\mathrm{SPD}}\left(P_{1},P_{2};t\right)$), we have $|\gamma_{P}\left(P_{1},P_{2};t\right)\left|=|\Sigma \right|$. Although the submanifold $P_{c}=\left\{P\in P\;:\;|P|=c\right\}$ is totally geodesic with respect to the trace metric, it is not totally geodesic with respect to $\frac{1}{2}\mathrm{tr}\left({\left(\bar{P}d\bar{P}\right)}^{2}\right)$. Thus, although $\gamma_{P}\left(P_{1},P_{2}\right)\in \bar{N}$, it does not correspond to the embedded MVN geodesics with respect to the Fisher metric. The C&O distance between two MVNs $N(\mu_{1},\Sigma )$ and $N(\mu_{2},\Sigma )$ sharing the same covariance matrix [19] is

(23) $$\rho_{\mathrm{CO}}\left(N\left(\mu_{1},\Sigma \right),N\left(\mu_{2},\Sigma \right)\right)=\mathrm{arccosh}\left(1+\frac{1}{2}\Delta_{\Sigma }^{2}\left(\mu_{1},\mu_{2}\right)\right),$$

where $\mathrm{arccosh}\left(x\right):=\log\left(x+\sqrt{x^{2}-1}\right)$ for x≥1 and $\Delta_{\Sigma }\left(\mu_{1},\mu_{2}\right)$ is the Mahalanobis distance between $N(\mu_{1},\Sigma )$ and $N(\mu_{2},\Sigma )$. In that case, we thus have $\rho_{\mathrm{CO}}\left(N\left(\mu_{1},\Sigma \right),N\left(\mu_{2},\Sigma \right)\right)=h_{\mathrm{CO}}\left(\Delta_{\Sigma }\left(\mu_{1},\mu_{2}\right)\right)$ where $h_{\mathrm{CO}}\left(u\right)=\mathrm{arccosh}\left(1+\frac{1}{2}u^{2}\right)$ is a strictly monotone increasing function. Let us note in passing that in [19] (Corollary, page 230) there is a confusing or typographic error since the distance is reported as $\mathrm{arccosh}\left(1+\frac{1}{2}d_{M}\left(\mu_{1},\mu_{2}\right)\right)$ where $d_{M}$ denotes “Mahalanobis distance” [51]. Therefore, either $d_{M}=\Delta_{\Sigma }^{2}$, Mahalanobis $D^{2}$-distance, or there is a missing square in the equation of the Corollary page 230. To obtain a flavor of how good is the approximation of the C&O distance, we may consider the same covariance case where we have both closed-form solutions for $\rho_{N}$ (Equation (20)) and $\rho_{\mathrm{CO}}$ (Equation (23)). Figure 3 plots the two functions $h_{\mathrm{CO}}$ and $h_{\mathrm{FR}}$ (with $h_{\mathrm{CO}}\left(u\right)\leq h_{\mathrm{FR}}\left(u\right)\leq u$ for u∈[0,∞)).

Let us remark that similarly all *f*-divergences between $N_{1}=\left(\mu_{1},\Sigma \right)$ and $N_{2}=\left(\mu_{2},\Sigma \right)$ are scalar functions of their Mahalanobis distance $\Delta_{\Sigma }\left(\mu_{1},\mu_{2}\right)$ too, see [62].

The C&O distance $\rho_{\mathrm{CO}}$ is a metric distance that has been used in many applications ranging from computer vision [57,67,68,69] to signal/sensor processing, statistics [70,71], machine learning [29,72,73,74,75,76] and analogical reasoning [77].

**Remark 3.**  *In a second paper, Calvo and Oller [32] noticed that we can embed normal distributions in P(d+1) by the following more general mapping (Lemma 3.1 [32]):*

(24) $$g_{\alpha ,\beta ,\gamma }\left(\mu ,\Sigma \right)={\left|\Sigma \right|}^{\alpha }\left[\begin{array}{cc} \Sigma +\beta \gamma^{2}\mu \mu^{⊤} & \beta \gamma \mu \\ \beta \gamma \mu^{⊤} & \beta \end{array}\right]\in P\left(d+1\right),$$

*where α∈R, $\beta \in R_{>0}$ and γ∈R. It is show in [32] that the induced length element is*

$$\begin{array}{ccc} ds_{\alpha ,\beta ,\gamma }^{2} & = & \frac{1}{2}\left(\alpha \left(\left(d+1\right)+2\alpha \right){\mathrm{tr}}^{2}\left(\Sigma^{-1}d\Sigma \right)+\mathrm{tr}\left({\left(\Sigma^{-1}d\Sigma \right)}^{2}\right)\right.\\ & & \left.+2\beta \gamma^{2}d\mu^{⊤}\Sigma^{-1}d\mu +2\alpha \mathrm{tr}\left(\Sigma^{-1}d\Sigma \right)\frac{d\beta }{\beta }+{\left(\frac{d\beta }{\beta }\right)}^{2}\right). \end{array}$$

*When γ=β=1, we have*

$$ds_{\alpha }^{2}=\frac{1}{2}\left(\alpha \left(\left(d+1\right)+2\alpha \right){\mathrm{tr}}^{2}\left(\Sigma^{-1}d\Sigma \right)+\mathrm{tr}\left({\left(\Sigma^{-1}d\Sigma \right)}^{2}\right)+2\beta \gamma^{2}d\mu^{⊤}\Sigma^{-1}d\mu \right).$$

*Thus, to cancel the term ${\mathrm{tr}}^{2}\left(\Sigma^{-1}d\Sigma \right)$, we may either choose α=0 or $\alpha =-\frac{2}{1+d}$.*

*In some applications [78], the embedding*

(25) $$g_{-\frac{1}{d+1},1,1}\left(\mu ,\Sigma \right)={\left|\Sigma \right|}^{-\frac{1}{d+1}}\left[\begin{array}{cc} \Sigma +\mu \mu^{⊤} & \mu \\ \mu^{⊤} & 1 \end{array}\right]:=\hat{f}\left(\mu ,\Sigma \right),$$

*is used to ensure that $\left|g_{-\frac{1}{d+1},1,1}\left(\mu ,\Sigma \right)\right|=1$. That is normal distributions are embedded diffeomorphically into the submanifold of positive–definite matrices with a unit determinant (also called SSPD, acronym of Special SPD). In [32], C&O showed that there exists a second isometric embedding of the Fisher–Rao Gaussian manifold N(d) into a submanifold of the cone P(d+1): $f_{\mathrm{SSPD}}\left(\mu ,\Sigma \right)={\left|\Sigma \right|}^{-\frac{2}{d+1}}\left[\begin{array}{cc} \Sigma +\mu \mu^{⊤} & \mu \\ \mu^{⊤} & 1 \end{array}\right]$. Let $\hat{P}=f_{\mathrm{SSPD}}\left(\mu ,\Sigma \right)$. This mapping can be understood as taking the elliptic isometry $P\mapsto {\left|P\right|}^{-\frac{2}{d+1}}P$ of P∈P(d+1) [64] since $\left|\Sigma |=\right|\bar{P}\left(\mu ,\Sigma \right)|$ (see proof in Proposition 3). It follows that*

$$\rho_{\mathrm{CO}}\left(N_{1},N_{2}\right)=\rho_{P}\left({\bar{P}}_{1},{\bar{P}}_{2}\right)=\rho_{P}\left({\hat{P}}_{1},{\hat{P}}_{2}\right)\leq \rho_{N}\left(N_{1},N_{2}\right).$$

*Similarly, we could have mapped $P\mapsto P^{-1}$ to obtain another isometric embedding. See the four types of elliptic isometric of the SPD cone described in [64]. Finally, let us remark that the SSPD submanifold is totally geodesic with respect to the trace metric but not with respect to the C&O metric.*

Interestingly, Calvo and Oller [48] (p. 131) proved that $\left(\left({\bar{\mu }}_{1},\ldots ,{\bar{\mu }}_{d}\right),\mathrm{diag}\left({\bar{\sigma }}_{1}^{2},\ldots ,{\bar{\sigma }}_{d}^{2}\right)\right)$ is a maximal invariant for the action of the affine group Aff(d), where $\bar{\mu }=Q^{-1}\left(\mu_{2}-\mu_{1}\right)$ and $\Sigma_{2}\Sigma_{1}^{-1}=Q\;\mathrm{diag}\left({\bar{\sigma }}_{1}^{2},\ldots ,{\bar{\sigma }}_{d}^{2}\right)\;Q^{-1}$ (in [48], the authors considered $\Sigma_{1}\Sigma_{1}^{-2}$). Thus, we consider the following dissimilarity

(26) $$D_{\mathrm{CO}}\left(N\left(\mu_{1},\Sigma_{1}\right),N\left(\mu_{2},\Sigma_{2}\right)\right)=\sqrt{2}\;\sqrt{\sum_{i=1}^{d}\log^{2}\left(\frac{1+\Delta (0,1;{\bar{\mu }}_{i},{\bar{\sigma }}_{i})}{1-\Delta (0,1;{\bar{\mu }}_{i},{\bar{\sigma }}_{i})}\right)}.$$

Dissimilarity $D_{\mathrm{CO}}$ is symmetric (i.e., $D_{\mathrm{CO}}\left(N_{1},N_{2}\right)=D_{\mathrm{CO}}\left(N_{2},N_{1}\right)$) and $D_{\mathrm{CO}}\left(N_{1},N_{2}\right)=0$ if and only if $N_{1}=N_{2}$. Please note that when d=1, $D_{\mathrm{CO}}$ is different from the Fisher–Rao distance of Equation (7).

## 3. Approximating the Fisher–Rao Distance

### 3.1. Approximating Length of Curves

Recall that the Fisher–Rao’s distance [79] is the Riemannian geodesic distance

$$\rho_{N}\left(N\left(\lambda_{1}\right),N\left(\lambda_{2}\right)\right)=\underset{\begin{array}{c} c(t)\\ c\left(0\right)=p_{\lambda_{1}}\\ c\left(1\right)=p_{\lambda_{2}} \end{array}}{\inf}\;\left\{\mathrm{Length}(c)\right\},$$

where

$$\mathrm{Length}\left(c\right)=\int_{0}^{1}\underset{ds_{N}\left(t\right)}{\underbrace{{\sqrt{\langle \dot{c}\left(t\right),\dot{c}\left(t\right)\rangle }}_{c(t)}}}dt.$$

**Figure 4:**
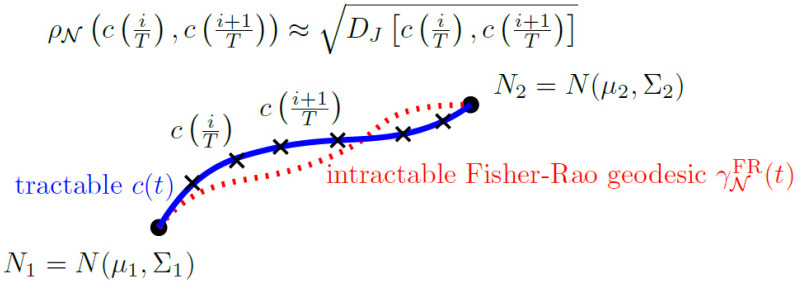
Approximating the Fisher–Rao geodesic distance $\rho_{N}\left(N_{1},N_{2}\right)$: The Fisher–Rao geodesic $\gamma_{N}^{\mathrm{FR}}$ is not known in closed form. We consider a tractable curve c(t), discretize c(t) at T+1 points $c(\frac{i}{T})$ with $c\left(0\right)=N_{1}$ and $c\left(1\right)=N_{2}$, and approximate $\rho_{N}\left(c\left(\frac{i}{T}\right),c\left(\frac{i+1}{T}\right)\right)$ by $\sqrt{D_{J}\left[c\left(\frac{i}{T}\right),c\left(\frac{i+1}{T}\right)\right]}$, considering that different tractable curves c(t) yield different approximations.

We can approximate the Rao distance $\rho_{N}\left(N_{1},N_{2}\right)$ by discretizing regularly any smooth curve c(t) joining $N_{1}=c\left(0\right)$ to $N_{2}=c\left(1\right)$ (Figure 4):

$$\rho_{N}\left(N_{1},N_{2}\right)\leq \frac{1}{T}\sum_{i=1}^{T-1}\rho_{N}\left(c\left(\frac{i}{T}\right),c\left(\frac{i+1}{T}\right)\right),$$

with equality holding iff $c\left(t\right)=\gamma_{N}\left(N_{1},N_{2};t\right)$ is the Riemannian geodesic defined by the Levi–Civita metric connection induced by the Fisher information metric.

When the number of discretization steps *T* is sufficiently large, the normal distributions $c\left(\frac{i}{T}\right)$ and $c\left(\frac{i+1}{T}\right)$ are close to each other, and we can approximate $\rho_{N}\left(c\left(\frac{i}{T}\right),c\left(\frac{i+1}{T}\right)\right)$ by $\sqrt{D_{J}\left[c\left(\frac{i}{T}\right),c\left(\frac{i+1}{T}\right)\right]}$, where $D_{J}\left[N_{1},N_{2}\right]=D_{\mathrm{KL}}\left[N_{1},N_{2}\right]+D_{\mathrm{KL}}\left[N_{2},N_{1}\right]$ is Jeffreys divergence, and $D_{\mathrm{KL}}$ is the Kullback–Leibler divergence:

$$D_{\mathrm{KL}}\left[p_{\left(\mu_{1},\Sigma_{1}\right)}:p_{\left(\mu_{2},\Sigma_{2}\right)}\right]=\frac{1}{2}\left(\mathrm{tr}\left(\Sigma_{2}^{-1}\Sigma_{1}\right)+\Delta \mu^{⊤}\Sigma_{2}^{-1}\Delta \mu -d+\log\frac{|\Sigma_{2}|}{|\Sigma_{1}|}\right).$$

Thus, the costly determinant computations cancel each other in Jeffreys divergence (i.e., $\log\frac{|\Sigma_{2}|}{|\Sigma_{1}|}+\log\frac{|\Sigma_{1}|}{|\Sigma_{2}|}=0$) and we have:

$$D_{J}\left[p_{\left(\mu_{1},\Sigma_{1}\right)}:p_{\left(\mu_{2},\Sigma_{2}\right)}\right]=\mathrm{tr}\left(\frac{\Sigma_{2}^{-1}\Sigma_{1}+\Sigma_{1}^{-1}\Sigma_{2}}{2}-I\right)+\Delta \mu^{⊤}\frac{\Sigma_{1}^{-1}+\Sigma_{2}^{-1}}{2}\Delta \mu .$$

Figure 4 summarizes our method to approximate the Fisher–Rao geodesic distance.

In general, it holds that

$$I_{f}\left[p:q\right]\approx \frac{f^{\prime \prime }\left(1\right)}{2}ds_{\mathrm{Fisher}}^{2},$$

between infinitesimally close distributions *p* and *q* ($ds\approx \sqrt{\frac{2\;I_{f}\left[p:q\right]}{f^{\prime \prime }\left(1\right)}}$), where $I_{f}\left[\cdot :\cdot \right]$ denotes a *f*-divergence [1]. The Jeffreys divergence is a *f*-divergence obtained for $f_{J}\left(u\right)=-\log u+u\log u$ with $f_{J}^{\prime \prime }\left(1\right)=2$. It is thus interesting to find low computational cost *f*-divergences between multivariate normal distributions to approximate the infinitesimal length element ds. Please note that *f*-divergences between MVNs are also invariant under the action of the affine group [62]. Thus, for infinitesimally close distributions *p* and *q*, this informally explains that $ds_{\mathrm{Fisher}}$ is invariant under the action of the affine group (see Proposition 1).

Although the definite integral of the length element along the Fisher–Rao geodesic $\gamma_{N}^{\mathrm{FR}}$ is not known in closed form (i.e., Fisher–Rao distance), the integral of the squared length element along the mixture geodesic $\gamma_{N}^{m}\left(N_{1},N_{2}\right)$ and exponential geodesic $\gamma_{N}^{e}\left(N_{1},N_{2}\right)$ coincide with Jeffreys divergence $D_{J}\left[N_{1},N_{2}\right]$ between $N_{1}$ and $N_{2}$ [1]:

**Property 3**  ([1])**.** *We have*

$$D_{J}\left[p_{\lambda_{1}},p_{\lambda_{2}}\right]=\int_{0}^{1}ds_{N}^{2}\left(\gamma_{N}^{m}\left(p_{\lambda_{1}},p_{\lambda_{2}};t\right)\right)dt=\int_{0}^{1}ds_{N}^{2}\left(\gamma_{N}^{e}\left(p_{\lambda_{1}},p_{\lambda_{2}};t\right)\right)dt.$$

**Proof.**  Let us report a proof of this remarkable fact in the general setting of Bregman manifolds. Indeed, since

$$D_{J}\left[p_{\lambda_{1}},p_{\lambda_{2}}\right]=D_{\mathrm{KL}}\left[p_{\lambda_{1}}:p_{\lambda_{2}}\right]+D_{\mathrm{KL}}\left[p_{\lambda_{2}}:p_{\lambda_{1}}\right],$$

and $D_{\mathrm{KL}}\left[p_{\lambda_{1}}:p_{\lambda_{2}}\right]=B_{F}\left(\theta \left(\lambda_{2}\right):\theta \left(\lambda_{1}\right)\right)$, where $B_{F}$ denotes the Bregman divergence induced by the cumulant function of the multivariate normals and θ(λ) is the natural parameter corresponding to λ, we have

$$\begin{array}{ccc} D_{J}\left[p_{\lambda_{1}},p_{\lambda_{2}}\right] & = & B_{F}\left(\theta_{1}:\theta_{2}\right)+B_{F}\left(\theta_{2}:\theta_{1}\right),\\ & = & S_{F}\left(\theta_{1};\theta_{2}\right)={\left(\theta_{2}-\theta_{1}\right)}^{⊤}\left(\eta_{2}-\eta_{1}\right)=S_{F^{*}}\left(\eta_{1};\eta_{2}\right), \end{array}$$

where η=∇F(θ) and $\theta =\nabla F^{*}\left(\eta \right)$ denote the dual parameterizations obtained by the Legendre–Fenchel convex conjugate $F^{*}\left(\eta \right)$ of F(θ). Moreover, we have $F^{*}\left(\eta \right)=-h\left(p_{\mu ,\Sigma }\right)$ [1], i.e., the convex conjugate function is Shannon negentropy.

Then we conclude using the fact that $S_{F}\left(\theta_{1};\theta_{2}\right)=\int_{0}^{1}ds^{2}\left(\gamma \left(t\right)\right)dt=\int_{0}^{1}ds^{2}\left(\gamma^{*}\left(t\right)\right)dt$, i.e., the symmetrized Bregman divergence amounts to integral energies on dual geodesics on a Bregman manifold. The proof of this general property is reported in Appendix E. □

It follows the following upper bound on the Fisher–Rao distance:

**Property 4**  (Fisher–Rao upper bound)**.** *The Fisher–Rao distance between normal distributions is upper bounded by the square root of the Jeffreys divergence: $\rho_{N}\left(N_{1},N_{2}\right)\leq \sqrt{D_{J}\left(N_{1},N_{2}\right)}$.*

**Proof.**  Consider the Cauchy–Schwarz inequality for positive functions f(t) and g(t): $\int_{0}^{1}f\left(t\right)g\left(t\right)dt\leq \sqrt{\left(\int_{0}^{1}f{\left(t\right)}^{2}dt\right)\left(\int_{0}^{1}g{\left(t\right)}^{2}dt\right)}$), and let $f\left(t\right)=ds_{N}(\gamma_{N}^{c}\left(p_{\lambda_{1}},p_{\lambda_{2}};t\right)$ and g(t)=1. Then we obtain:

$${\left(\int_{0}^{1}ds_{N}(\gamma_{N}^{c}\left(p_{\lambda_{1}},p_{\lambda_{2}};t\right)dt\right)}^{2}\leq \left(\int_{0}^{1}ds_{N}^{2}(\gamma_{N}^{c}\left(p_{\lambda_{1}},p_{\lambda_{2}};t\right)dt\right)\left(\underset{=1}{\underbrace{\int_{0}^{1}1^{2}dt}}\right).$$

Furthermore, since by definition of $\gamma_{N}^{\mathrm{FR}}$, we have

$$\int_{0}^{1}ds_{N}(\gamma_{N}^{c}\left(p_{\lambda_{1}},p_{\lambda_{2}};t\right)dt\geq \int_{0}^{1}ds_{N}(\gamma_{N}^{\mathrm{FR}}\left(p_{\lambda_{1}},p_{\lambda_{2}};t\right)dt=:\rho_{N}\left(N_{1},N_{2}\right).$$

It follows for $c=\gamma_{N}^{e}$ (i.e., *e*-geodesic) using Property 3 that we have:

$$\rho_{N}{\left(N_{1},N_{2}\right)}^{2}\leq \int_{0}^{1}ds_{N}^{2}(\gamma_{N}^{e}\left(p_{\lambda_{1}},p_{\lambda_{2}};t\right)dt=D_{J}\left(N_{1},N_{2}\right).$$

Thus, we conclude that $\rho_{N}\left(N_{1},N_{2}\right)\leq \sqrt{D_{J}\left(N_{1},N_{2}\right)}$.

Please note that in Riemannian geometry, a curve γ minimizes the energy $E\left(\gamma \right)=\int_{0}^{1}{∥\dot{\gamma }\left(t\right)∥}^{2}dt$ if it minimizes the length $L\left(\gamma \right)=\int_{0}^{1}∥\dot{\gamma }\left(t\right)∥dt$ and $∥\dot{\gamma }\left(t\right)∥$ is constant. Using Cauchy-Schwartz inequality, we can show that L(γ)≤E(γ). □

**Figure 5:**
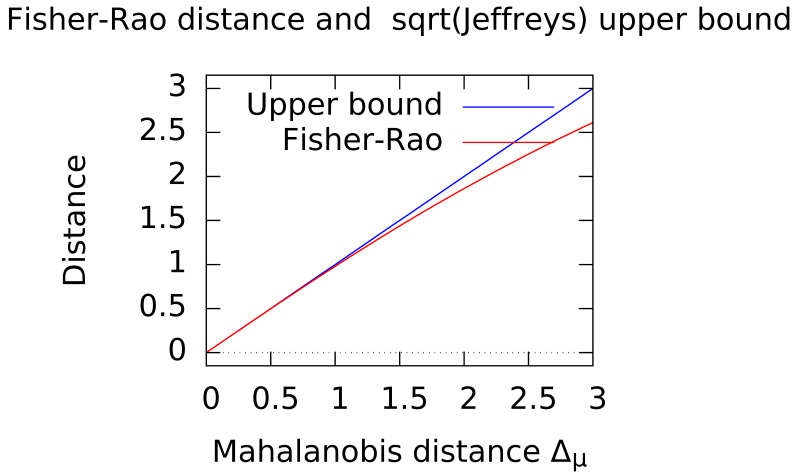
Quality of the $\sqrt{D_{J}}$ upper bound on the Fisher–Rao distance $\rho_{N}$ when normal distributions have the same covariance matrix.

This upper bound is tight at infinitesimal scale (i.e., when $N_{2}=N_{1}+dN$) since $\rho_{N}\left(N_{1},N_{2}\right)\approx ds_{N}\left(N_{1}\right)\approx \sqrt{\frac{2\;I_{f}\left[N_{1}:N_{2}\right]}{f^{\prime \prime }\left(1\right)}}$ and the *f*-divergence in right-hand side of the identity can be chosen as Jeffreys divergence. To appreciate the quality of the square root of Jeffreys divergence upper bound of Property 4, consider the case where $N_{1},N_{2}\in M_{\Sigma }$. In that case, we have $\rho_{N}\left(N\left(\mu_{1},\Sigma \right),N\left(\mu_{2},\Sigma \right)\right)=\sqrt{2}\;\mathrm{arccosh}\left(1+\frac{1}{4}\Delta_{\Sigma }^{2}\left(\mu_{1},\mu_{2}\right)\right)$ and $\sqrt{D_{J}\left[N\left(\mu_{1},\Sigma \right),N\left(\mu_{2},\Sigma \right)\right]}=\Delta_{\Sigma }\left(\mu_{1},\mu_{2}\right)$ (since $D_{\mathrm{KL}}\left[N\left(\mu_{1},\Sigma \right),N\left(\mu_{2},\Sigma \right)\right]=\frac{1}{2}\Delta_{\Sigma }^{2}\left(\mu_{1},\mu_{2}\right)$). The upper bound can thus be checked since we have $\sqrt{2}\;\mathrm{arccosh}\left(1+\frac{1}{4}x^{2}\right)\leq x$ for x≥0. The plots of Figure 5 shows visually the quality of the $\sqrt{D_{J}}$ upper bound.

For any smooth curve c(t), we can thus approximate $\rho_{N}$ for large *T* by

(27) $${\tilde{\rho }}_{N}^{c}\left(N_{1},N_{2}\right):=\frac{1}{T}\sum_{i=1}^{T-1}\sqrt{D_{J}\left[c\left(\frac{i}{T}\right),c\left(\frac{i+1}{T}\right)\right].}$$

For example, we may consider the following curves on $M_{N}$ which admit closed-form parameterizations in t∈[0,1]:

- linear interpolation (LERP, Linear intERPolation) $c_{\lambda }\left(t\right)=t\left(\mu_{1},\Sigma_{1}\right)+\left(1-t\right)\left(\mu_{2},\Sigma_{2}\right)$ between $\left(\mu_{1},\Sigma_{1}\right)$ and $\left(\mu_{2},\Sigma_{2}\right)$,
- the mixture geodesic [80] $c_{m}\left(t\right)=\gamma_{N}^{m}\left(N_{1},N_{2};t\right)=\left(\mu_{t}^{m},\Sigma_{t}^{m}\right)$ with $\mu_{t}^{m}={\bar{\mu }}_{t}$ and $\Sigma_{t}^{m}={\bar{\Sigma }}_{t}+t\mu_{1}\mu_{1}^{⊤}+\left(1-t\right)\mu_{2}\mu_{2}^{⊤}-{\bar{\mu }}_{t}{\bar{\mu }}_{t}^{⊤}$ where ${\bar{\mu }}_{t}=t\mu_{1}+\left(1-t\right)\mu_{2}$ and ${\bar{\Sigma }}_{t}=t\Sigma_{1}+\left(1-t\right)\Sigma_{2}$,
- the exponential geodesic [80] $c_{e}\left(t\right)=\gamma_{N}^{e}\left(N_{1},N_{2};t\right)=\left(\mu_{t}^{e},\Sigma_{t}^{e}\right)$ with $\mu_{t}^{e}={\bar{\Sigma }}_{t}^{H}\left(t\Sigma_{1}^{-1}\mu_{1}+\left(1-t\right)\Sigma_{2}^{-1}\mu_{2}\right)$ and $\Sigma_{t}^{e}={\bar{\Sigma }}_{t}^{H}$ where ${\bar{\Sigma }}_{t}^{H}={\left(t\Sigma_{1}^{-1}+\left(1-t\right)\Sigma_{2}^{-1}\right)}^{-1}$ is the matrix harmonic mean,
- the curve $c_{em}\left(t\right)=\frac{1}{2}\left(\gamma_{N}^{e}\left(N_{1},N_{2};t\right)+\gamma_{N}^{m}\left(N_{1},N_{2};t\right)\right)$ which is obtained by averaging the mixture geodesic with the exponential geodesic.

**Figure 6:**
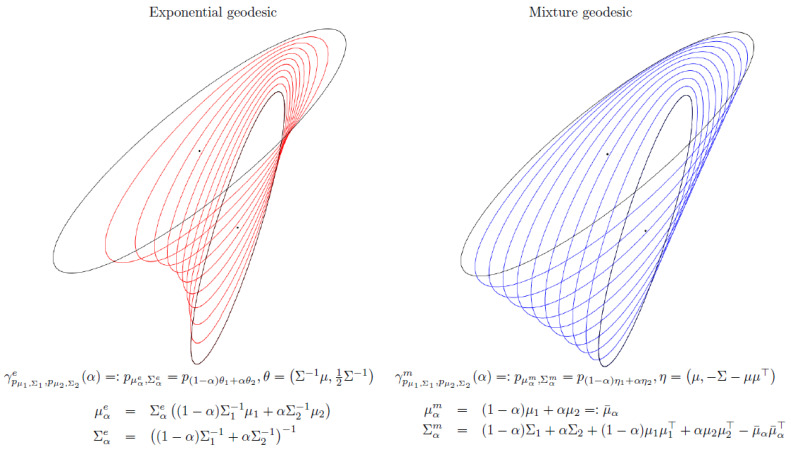
Visualizing the exponential and mixture geodesics between two bivariate normal distributions.

Figure 6 visualizes the exponential and mixture geodesics between two bivariate normal distributions.

Let us denote by ${\tilde{\rho }}_{N}^{\lambda }={\tilde{\rho }}_{N}^{c_{\lambda }}$, ${\tilde{\rho }}_{N}^{m}={\tilde{\rho }}_{N}^{c_{m}}$, ${\tilde{\rho }}_{N}^{e}={\tilde{\rho }}_{N}^{c_{e}}$ and ${\tilde{\rho }}_{N}^{em}={\tilde{\rho }}_{N}^{c_{em}}$ the approximations obtained by these curves following from Equation (27). When *T* is sufficiently large, the approximated distances ${\tilde{\rho }}^{x}$ are close to the length of curve *x*, and we may thus consider a set of several curves ${\left\{c_{i}\right\}}_{i\in I}$ and report the smallest Fisher–Rao distance approximations obtained among these curves: $\rho_{N}\left(N_{1},N_{2}\right)\approx \min_{i\in I}{\tilde{\rho }}_{N}^{c_{i}}\left(N_{1},N_{2}\right)$.

Please note that we consider the regular spacing for approximating a curve length and do not optimize the position of the sample points on the curve. Indeed, as T→∞, the curve length approximation tends to the Riemannian curve length. In other words, we can measure approximately finely the length of any curve available with closed-form reparameterization by increasing *T*. Thus, the key question of our method is how to best approximate the Fisher–Rao geodesic by a curve that can be parametrized by a closed-form formula and is close enough to the Fisher–Rao geodesic.

Next, we introduce our approximation curve $c_{\mathrm{CO}}\left(t\right)$ derived from Calvo and Oller isometric mapping *f* which experimentally behaves better when normals are not *too far* from each other.

### 3.2. A Curve Derived from Calvo and Oller’s Embedding

**Figure 7:**
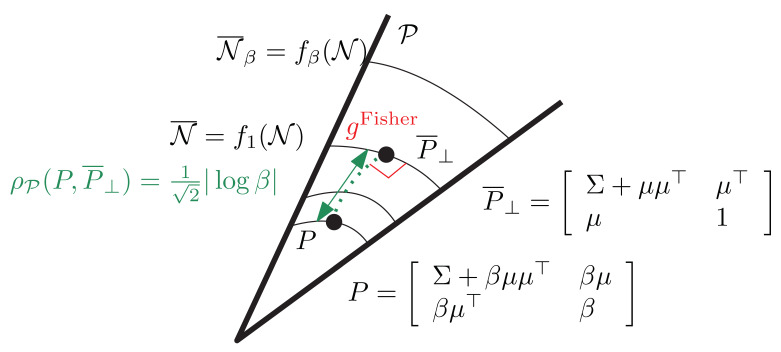
Projecting an SPD matrix P∈P onto $\bar{N}=f\left(N\right)$: $\gamma_{P}\left(P,{\bar{P}}_{⊥}\right)$ is orthogonal to $\bar{N}$ with respect to the trace metric.

**Figure 8:**
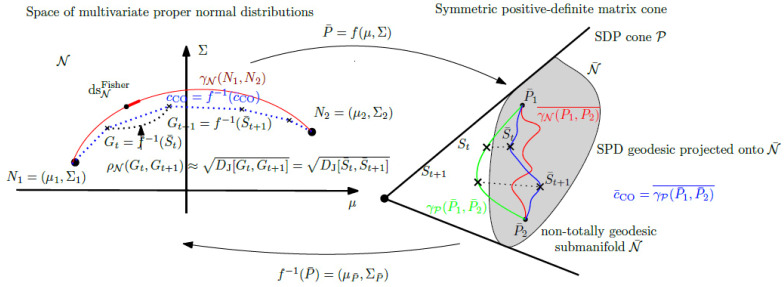
Illustration of the approximation of the Fisher–Rao distance between two multivariate normals $N_{1}$ and $N_{2}$ (red geodesic length $\gamma_{N}\left(N_{1},N_{2}\right)$ by discretizing curve ${\bar{c}}_{\mathrm{CO}}\in \bar{N}$ or equivalently curve $c_{\mathrm{CO}}\in N$.

This approximation consists of leveraging the closed-form expression of the SPD geodesics [63,66]:

$$\gamma_{P}\left(P,Q;t\right)=P^{\frac{1}{2}}\;{\left(P^{-\frac{1}{2}}Q^{\frac{1}{2}}P^{-\frac{1}{2}}\right)}^{t}\;P^{\frac{1}{2}},\;t\in \left[0,1\right]$$

to approximate the Fisher–Rao normal geodesic $\gamma_{N}^{\mathrm{Fisher}}\left(N_{1},N_{2};t\right)$ as follows: Let ${\bar{P}}_{1}=f\left(N_{1}\right),{\bar{P}}_{2}=f\left(N_{2}\right)\in \bar{N}$, and consider the smooth curve

(28) $${\bar{c}}_{\mathrm{CO}}\left({\bar{P}}_{1},{\bar{P}}_{2};t\right)={\mathrm{proj}}_{\bar{N}}\left(\gamma_{P}\left({\bar{P}}_{1},{\bar{P}}_{2};t\right)\right),$$

where ${\mathrm{proj}}_{\bar{N}}\left(P\right)$ denotes the orthogonal projection of P∈P(d+1) onto $\bar{N}$ (Figure 7). Thus, curve $c_{\mathrm{CO}}\left(t\right)$ (t∈[0,1]) is then defined by taking the inverse mapping $f^{-1}\left({\bar{c}}_{\mathrm{CO}}\right)$ (Figure 8):

(29) $$c_{\mathrm{CO}}\left(t\right)=f^{-1}\left({\mathrm{proj}}_{\bar{N}}\left(\gamma_{P}\left({\bar{P}}_{1},{\bar{P}}_{2};t\right)\right)\right).$$

Please note that the matrix power $P^{t}$ can be computed as $P^{t}=U\;\mathrm{diag}\left(\lambda_{1}^{t},\ldots ,\lambda_{d}^{t}\right)\;V^{⊤}$ where $P=U\;\mathrm{diag}\left(\lambda_{1}^{t},\ldots ,\lambda_{d}^{t}\right)\;V^{⊤}$ is the eigenvalue decomposition of *P*.

Let us now explain how to project $P=\left[P_{i,j}\right]\in P\left(d+1\right)$ onto $\bar{N}$ based on the analysis of the Appendix of [19] (p. 239):

**Proposition 2**  (Projection of an SPD matrix onto the embedded normal submanifold $\bar{N}$)**.** *Let $\beta =P_{d+1,d+1}$ and write $P=\left[\begin{array}{cc} \Sigma +\beta \mu \mu^{⊤} & \beta \mu \\ \beta \mu^{⊤} & \beta \end{array}\right]$. Then the orthogonal projection at P∈P onto $\bar{N}$ is:*

(30) $${\bar{P}}_{⊥}:={\mathrm{proj}}_{\bar{N}}\left(P\right)=\left[\begin{array}{cc} \Sigma +\mu \mu^{⊤} & \mu^{⊤}\\ \mu & 1 \end{array}\right],$$

*and the SPD distance between P and ${\bar{P}}_{⊥}$ is*

(31) $$\rho_{P}\left(P,{\bar{P}}_{⊥}\right)=\frac{1}{\sqrt{2}}\left|\log\beta \right|.$$

Notice that the projection of *P* is easily computed since $\beta =P_{d+1,d+1}$.

$${\mathrm{proj}}_{\bar{N}}\left(\left[\begin{array}{cc} \Sigma +\beta \mu \mu^{⊤} & \beta \mu \\ \beta \mu^{⊤} & \beta \end{array}\right]\right)=\left[\begin{array}{cc} \Sigma +\mu \mu^{⊤} & \mu^{⊤}\\ \mu & 1 \end{array}\right]$$

**Figure 9:**
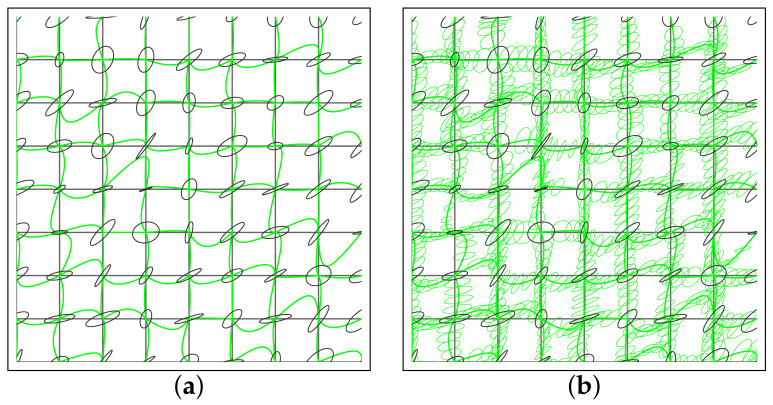
Diffusion tensor imaging (DTI) on a 2D grid: (**a**) Ellipsoids shown at the 8×8 grid locations with C&O curves in green, and (**b**) some interpolated ellipsoids are further shown along the C&O curves.

**Remark 4.**  *In Diffusion Tensor Imaging [39] (DTI), the Fisher–Rao distance can be used to evaluate the distance between three-dimensional normal distributions with means located at a 3D grid position. We may consider 3×3×3−1=26 neighbor graphs induced by the grid, and for each normal N of the grid, calculate the approximations of the Fisher–Rao distance of N with its neighbors $N^{\prime }$ as depicted in Figure 9. Then the distance between two tensors $N_{1}$ and $N_{2}$ of the 3D grid is calculated as the shortest path on the weighted graph using Dijkstra’s algorithm [39].*

**Figure 10:**
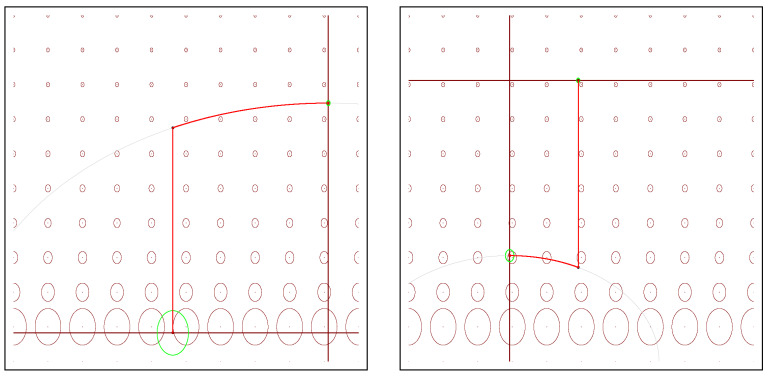
Examples of projection of N(μ,Σ) onto the submanifolds $M_{\mu_{0}}$ and $M_{\Sigma_{0}}$. Tissot indicatrices are rendered in green at the projected normal distributions $\left(\mu_{0},\Sigma +\frac{1}{2}\left(\mu_{0}-\mu \right){\left(\mu_{0}-\mu \right)}^{⊤}\right)$ and $\left(\mu ,\Sigma_{0}\right)$, respectively.

Please note that the Fisher–Rao projection of $N_{1}=\left(\mu_{1},\Sigma_{1}\right)$ onto a submanifold $M_{\mu_{2}}$ with fixed mean $\mu_{2}$ was recently reported in closed form in [72] (Equation (21)):

$$N^{*}=N\left(\mu_{2},\Sigma_{1}+\frac{1}{2}\left(\mu_{2}-\mu_{1}\right){\left(\mu_{2}-\mu_{1}\right)}^{⊤}\right),$$

with

$$\rho_{N}\left(N_{1},N^{*}\right)=\frac{1}{\sqrt{2}}\;\mathrm{arccosh}\left(d+{\left(\mu_{2}-\mu_{1}\right)}^{⊤}\Sigma_{1}^{-1}\left(\mu_{2}-\mu_{1}\right)\right),$$

and the Fisher–Rao projection of $N_{1}=\left(\mu_{1},\Sigma_{1}\right)$ onto submanifold $M_{\Sigma_{2}}$ is the “vertical projection” $N^{*}=\left(\mu_{1},\Sigma_{2}\right)$ (Figure 10) with

$$\rho_{N}\left(N_{1},N^{*}\right)=\rho_{N_{\mu }}\left(\Sigma_{1},\Sigma_{2}\right).$$

We can upper bound the Fisher–Rao distance $\rho_{N}\left(\left(\mu_{1},\Sigma_{1}\right),\left(\mu_{2},\Sigma_{2}\right)\right)$ by projecting $\Sigma_{1}$ onto $M_{\mu_{2}}$ and projecting $\Sigma_{2}$ onto $M_{\mu_{1}}$. Let $\Sigma_{12}\in M_{\mu_{2}}$ and $\Sigma_{21}\in M_{\mu_{1}}$ denote those Fisher–Rao orthogonal projections. Using the triangular inequality property of the Fisher–Rao distance, we obtain the following upper bounds:

(32) $$\begin{array}{ccc} \rho_{N}\left(\left(\mu_{1},\Sigma_{1}\right),\left(\mu_{2},\Sigma_{2}\right)\right) & \leq & \rho_{N}\left(\left(\mu_{1},\Sigma_{1}\right),\left(\mu_{2},\Sigma_{12}\right)\right)+\rho_{N}\left(\mu_{2},\Sigma_{12},\left(\mu_{2},\Sigma_{2}\right)\right), \end{array}$$

(33) $$\begin{array}{ccc} & \;\;\;\;\;\;\;\;\;\;\;\;\;\;\;\;\;\;\;\;\;\;\;\;\;\;\;\;\;\;\;\;\;\;\;\;\;\;\;\;\;\leq & \rho_{N}\left(\left(\mu_{2},\Sigma_{2}\right),\left(\mu_{1},\Sigma_{21}\right)\right)+\rho_{N}\left(\left(\mu_{1},\Sigma_{21}\right),\left(\mu_{1},\Sigma_{1}\right)\right). \end{array}$$

**Figure 11:**
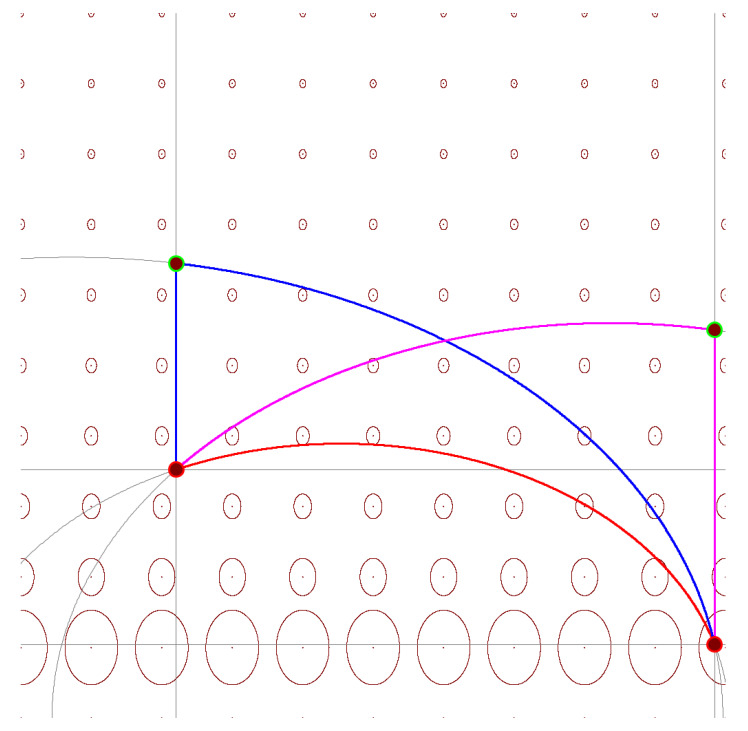
Upper bounding the Fisher–Rao’s distance $\rho_{N}\left(\left(\mu_{1},\Sigma_{1}\right),\left(\mu_{2},\Sigma_{2}\right)\right)$ (red points) using projections (green points) onto submanifolds with fixed means.

See Figure 11 for an illustration.

Let ${\bar{c}}_{\mathrm{CO}}\left(t\right)={\bar{S}}_{t}$ and $c_{\mathrm{CO}}\left(t\right)=f^{-1}\left(c_{\mathrm{CO}}\left(t\right)\right)=:G_{t}$. The following proposition shows that we have $D_{J}\left[{\bar{S}}_{t},{\bar{S}}_{t+1}\right]=D_{J}\left[G_{t},G_{t+1}\right]$.

**Proposition 3.**  *The Kullback–Leibler divergence between $p_{\mu_{1},\Sigma_{1}}$ and $p_{\mu_{2},\Sigma_{2}}$ amounts to the KLD between $q_{{\bar{P}}_{1}}=p_{0,f(\mu_{1},\Sigma_{1})}$ and $q_{{\bar{P}}_{2}}=p_{0,f(\mu_{2},\Sigma_{2})}$ where ${\bar{P}}_{i}=f\left(\mu_{i},\Sigma_{i}\right)$:*

$$D_{\mathrm{KL}}\left[p_{\mu_{1},\Sigma_{1}}:p_{\mu_{2},\Sigma_{2}}\right]=D_{\mathrm{KL}}\left[q_{{\bar{P}}_{1}}:q_{{\bar{P}}_{2}}\right].$$

The KLD between two centered (d+1)-variate normals $q_{P_{1}}=p_{0,P_{1}}$ and $q_{P_{2}}=p_{0,P_{2}}$ is

$$D_{\mathrm{KL}}\left[q_{P_{1}}:q_{P_{2}}\right]=\frac{1}{2}\left(\mathrm{tr}\left(P_{2}^{-1}P_{1}\right)-d-1+\log\frac{|P_{2}|}{|P_{1}|}\right).$$

This divergence can be interpreted as the matrix version of the Itakura–Saito divergence [81]. The SPD cone equipped with $\frac{1}{2}$ of the trace metric can be interpreted as Fisher–Rao centered normal manifolds: $\left(N_{\mu },g_{N_{\mu }}^{\mathrm{Fisher}}\right)=\left(P,\frac{1}{2}g^{\mathrm{trace}}\right)$.

Since the determinant of a block matrix is

$$\left|\left[\begin{array}{cc} A & B\\ C & D \end{array}\right]\right|=\left|A-BD^{-1}C\right|,$$

we obtain with D=1: $|f\left(\mu ,\Sigma \right)|=|\Sigma +\mu \mu^{⊤}-\mu \mu^{⊤}\left|=|\Sigma \right|$.

Let ${\bar{P}}_{1}=f\left(\mu_{1},\Sigma_{1}\right)$ and ${\bar{P}}_{2}=f\left(\mu_{2},\Sigma_{2}\right)$. Checking $D_{\mathrm{KL}}\left[p_{\mu_{1},\Sigma_{1}}:p_{\mu_{2},\Sigma_{2}}\right]=D_{\mathrm{KL}}\left[q_{{\bar{P}}_{1}}:q_{{\bar{P}}_{2}}\right]$ where $q_{\bar{P}}=p_{0,\bar{P}}$ amounts to verify that

$$\mathrm{tr}\left({\bar{P}}_{2}^{-1}{\bar{P}}_{1}\right)=1+\mathrm{tr}\left(\Sigma_{2}^{-1}\Sigma_{1}+\Delta_{\mu }^{⊤}\Sigma_{2}^{-1}\Delta_{\mu }\right).$$

Indeed, using the inverse matrix

$$f{\left(\mu ,\Sigma \right)}^{-1}=\left[\begin{array}{cc} \Sigma^{-1} & -\Sigma^{-1}\mu \\ -\mu^{⊤}\Sigma^{-1} & 1+\mu^{⊤}\Sigma^{-1}\mu \end{array}\right],$$

we have

$$\begin{array}{ccc} \mathrm{tr}({\bar{P}}_{2}^{-1}{\bar{P}}_{1}) & = & \mathrm{tr}\left(\left[\begin{array}{cc} \Sigma_{2}^{-1} & -\Sigma_{2}^{-1}\mu_{2}\\ -\mu_{2}^{⊤}\Sigma_{2}^{-1} & 1+\mu_{2}^{⊤}\Sigma_{2}^{-1}\mu_{2} \end{array}\right]\;\left[\begin{array}{cc} \Sigma_{1}+\mu_{1}\mu_{1}^{⊤} & \mu_{1}\\ \mu_{1}^{⊤} & 1 \end{array}\right]\right),\\ & = & 1+\mathrm{tr}(\Sigma_{2}^{-1}\Sigma_{1}+\Delta_{\mu }^{⊤}\Sigma_{2}^{-1}\Delta_{\mu }). \end{array}$$

Thus, even if the dimension of the sample spaces of $p_{\mu ,\Sigma }$ and $q_{\bar{P}=f\left(\mu ,\Sigma \right)}$ differs by one, we obtain the same KLD by Calvo and Oller’s isometric mapping *f*.

This property holds for the KLD/Jeffreys divergence $D_{J}$ but not for all *f*-divergences [1]$I_{f}$ in general (e.g., it fails for the Hellinger divergence).

**Figure 12:**
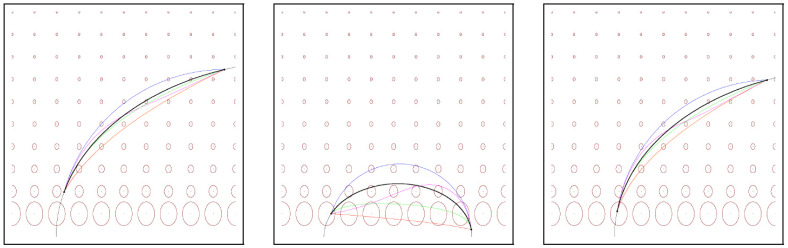
Geodesics and curves used to approximate the Fisher–Rao distance with the Fisher metric shown using Tissot’s indicatrices: exponential geodesic (red), mixture geodesic (blue), mid-exponential-mixture curve (purple), projected CO curve (green), and target Fisher–Rao geodesic (black). (Visualization in the parameter space of normal distributions).

Figure 12 shows the various geodesics and curves used to approximate the Fisher–Rao distance with the Fisher metric shown using Tissot indicatrices.

Please note that the introduction of parameter β is related to the foliation of the SPD cone P by $\left\{f_{\beta }\left(N\right)\;:\;\beta >0\right\}$: $P\left(d+1\right)=R_{>0}\times f_{\beta }\left(N\right)$. See Figure 7. Thus, we may define how good the projected C&O curve is to the Fisher–Rao geodesic by measuring the average distance between points on $\gamma_{P}\left({\bar{P}}_{1},{\bar{P}}_{2};t\right)$ and their projections ${\bar{\gamma_{P}\left({\bar{P}}_{1},{\bar{P}}_{2};t\right)}}^{⊥}$ onto $\bar{N}$:

$$\delta^{\mathrm{CO}}\left(N_{1},N_{2}\right)=\delta^{\mathrm{CO}}\left({\bar{P}}_{1},{\bar{P}}_{2}\right)=\int_{0}^{1}\rho_{P}\left(\gamma_{P}\left({\bar{P}}_{1},{\bar{P}}_{2};t\right),{\bar{\gamma_{P}\left({\bar{P}}_{1},{\bar{P}}_{2};t\right)}}^{⊥}\right)\;dt.$$

In practice, we evaluate this integral at the sampling points $S_{t}$:

(34) $$\delta^{\mathrm{CO}}\left(P_{1},P_{2}\right)\approx \delta_{T}^{\mathrm{CO}}\left(P_{1},P_{2}\right):=\frac{1}{T}\sum_{i=1}^{T}\rho_{P}\left(S_{t},{\bar{S}}_{t}\right),$$

where $S_{t}=\gamma_{P}\left({\bar{P}}_{1},{\bar{P}}_{2};t\right)$ and ${\bar{S}}_{t}=\gamma_{P}{\left({\bar{P}}_{1},{\bar{P}}_{2};t\right)}^{⊥}$. We checked experimentally (see Section 3.3) that for close by normals $N_{1}$ and $N_{1}$, we have $\delta^{\mathrm{CO}}\left({\bar{N}}_{1},{\bar{N}}_{2}\right)$ small, and that when $N_{1}$ becomes further separated from $N_{2}$, the average projection error $\delta^{\mathrm{CO}}\left({\bar{N}}_{1},{\bar{N}}_{2}\right)$ increases. Thus, $\delta_{T}^{\mathrm{CO}}\left(P_{1},P_{2}\right)$ is a good measure of the precision of our Fisher–Rao distance approximation.

**Lemma 1.**  *We have $\rho_{\bar{N}}\left({\bar{S}}_{t},{\bar{S}}_{t+1}\right)\leq \rho_{P}\left({\bar{S}}_{t},S_{t}\right)+\rho_{P}\left(S_{t},S_{t+1}\right)+\rho_{P}\left(S_{t+1},{\bar{S}}_{t+1}\right)$.*

**Proof.**  The proof consists of applying twice the triangle inequality of metric distance $\rho_{P}$:

$$\begin{array}{ccc} \rho_{\bar{N}}\left({\bar{S}}_{t},{\bar{S}}_{t+1}\right) & \leq & \rho_{P}\left({\bar{S}}_{t},S_{t+1}\right)+\rho_{P}\left(S_{t+1},{\bar{S}}_{t+1}\right),\\ & \leq & \rho_{P}\left({\bar{S}}_{t},S_{t}\right)+\rho_{P}\left(S_{t},S_{t+1}\right)+\rho_{P}\left(S_{t+1},{\bar{S}}_{t+1}\right). \end{array}$$

**Figure 13:**
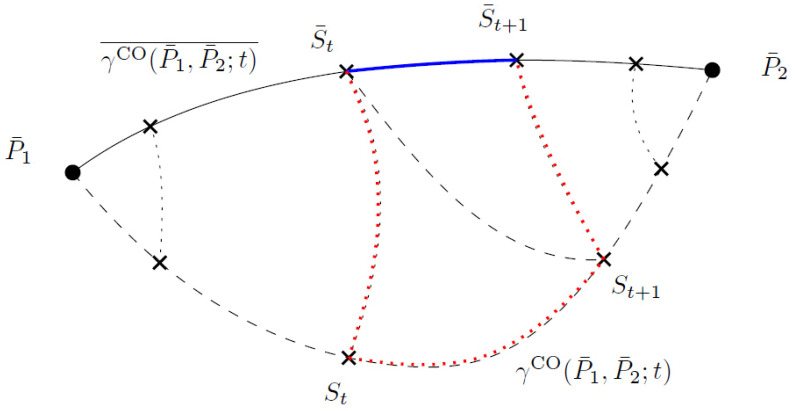
Bounding $\rho_{N}\left({\bar{S}}_{t},{\bar{S}}_{t+1}\right)$ using the triangular inequality of $\rho_{P}$ in the SPD cone P(d+1).

See Figure 13 where the left-hand-side geodesic length is shown in blue and the right-hand-side upper bound is visualized in red. □

**Property 5.**  *We have $\rho_{N}\left(N_{1},N_{2}\right)\leq \rho_{N}^{\mathrm{CO}}\left(N_{1},N_{2}\right)\leq \rho_{N}\left(N_{1},N_{2}\right)+2\;\delta_{T}^{\mathrm{CO}}\left({\bar{P}}_{1},{\bar{P}}_{2}\right)$.*

**Proof.**  At infinitesimal scale when $S_{t+1}\approx S_{t}$, using Lemma 1 and $\rho_{P}\left(S_{t+1},{\bar{S}}_{t+1}\right)\approx \rho_{P}\left({\bar{S}}_{t},S_{t}\right)$ we have

$$ds_{N}\left({\bar{S}}_{t}\right)\leq ds_{P}\left(S_{t}\right)+2\rho_{P}\left(S_{t},{\bar{S}}_{t}\right).$$

Taking the integral along the curve $c^{\mathrm{CO}}\left(t\right)=\bar{\gamma^{\mathrm{CO}}\left({\bar{P}}_{1},{\bar{P}}_{2};t\right)}$, we obtain

$$\rho_{N}^{\mathrm{CO}}\left(N_{1},N_{2}\right)\leq \rho_{P}\left({\bar{P}}_{1},{\bar{P}}_{2}\right)+2\delta_{T}^{\mathrm{CO}}\left({\bar{P}}_{1},{\bar{P}}_{2}\right)$$

Since $\rho_{P}\left({\bar{P}}_{1},{\bar{P}}_{2}\right)\leq \rho_{N}\left(N_{1},N_{2}\right)$, we have

$$\rho_{N}\left(N_{1},N_{2}\right)\leq \rho_{N}^{\mathrm{CO}}\left(N_{1},N_{2}\right)\leq \rho_{N}\left(N_{1},N_{2}\right)+2\delta_{T}^{\mathrm{CO}}\left({\bar{P}}_{1},{\bar{P}}_{2}\right).$$

□

Notice that $\sum_{i=0}^{T-1}\rho_{P}\left(S_{t},S_{t+1}\right)=\rho_{P}\left({\bar{P}}_{1},{\bar{P}}_{2}\right)$.

**Example 1.**  *Let us consider Example 1 of [42] (p. 11):*

$$N_{1}=\left(\left[\begin{array}{c} -1\\ 0 \end{array}\right],\Sigma \right),\;N_{2}=\left(\left[\begin{array}{c} 6\\ 3 \end{array}\right],\Sigma \right),\Sigma =\left[\begin{array}{cc} 1.1 & 0.9\\ 0.9 & 1.1 \end{array}\right].$$

*The Fisher–Rao distance is evaluated numerically in [42] as 5.00648. We have the lower bound $\rho_{N}^{\mathrm{CO}}\left(N_{1},N_{2}\right)=4.20447$, and the Mahalanobis distance 8.06226 upper bounds the Fisher–Rao distance (not totally geodesic submanifold $N_{\Sigma }$). Our projected C&O curve discretized with T=1000 yields an approximation ${\tilde{\rho }}_{N}^{\mathrm{CO}}\left(N_{1},N_{2}\right)=5.31667$. The average projection distance $\rho_{P}\left(S_{t},{\bar{S}}_{t}\right)$ is $\delta_{T}^{\mathrm{CO}}\left(N_{1},N_{2}\right)=0.61791$, and the maximum projected distance is 1.00685. We check that*

$$5.00648\approx \rho_{N}\left(N_{1},N_{2}\right)\leq {\tilde{\rho }}_{N}^{\mathrm{CO}}\left(N_{1},N_{2}\right)\approx 5.31667\leq \rho_{N}\left(N_{1},N_{2}\right)+2\delta_{T}^{\mathrm{CO}}\left({\bar{P}}_{1},{\bar{P}}_{2}\right)\approx 5.44028.$$

*The Killing distance [82] obtained for $\kappa_{\mathrm{Killing}}=2$ is $\rho_{\mathrm{Killing}}\left(N_{1},N_{2}\right)\approx 6.82028$ (see Appendix C). Notice that geodesic shooting is time-consuming compared to our approximation technique.*

### 3.3. Some Experiments

The KLD $D_{\mathrm{KL}}$ and Jeffreys divergence $D_{J}$, the Fisher–Rao distance $\rho_{N}$ and the Calvo and Oller distance $\rho_{\mathrm{CO}}$ are all invariant under the congruence action of the affine group $\mathrm{Aff}\left(d\right)=R^{d}⋊\mathrm{GL}\left(d\right)$ with the group operation

$$\left(a_{1},A_{1}\right)\left(a_{2},A_{2}\right)=\left(a_{1}+A_{1}a_{2},A_{1}A_{2}\right).$$

Let (A,a)∈Aff(d), and define the action on the normal space N as follows:

$$\left(A,a\right).N\left(\mu ,\Sigma \right)=N\left(A^{⊤}\mu +a,A\Sigma A^{⊤}\right).$$

Then we have:

$$\begin{array}{ccc} \rho_{N}\left(\left(A,a\right).N_{1},\left(A,a\right).N_{2}\right) & = & \rho_{N}\left(N_{1},N_{2}\right),\\ \rho_{\mathrm{CO}}\left(\left(A,a\right).N_{1},\left(A,a\right).N_{2}\right) & = & \rho_{\mathrm{CO}}\left(N_{1},N_{2}\right),\\ D_{\mathrm{KL}}\left[\left(A,a\right).N_{1}:\left(A,a\right).N_{2}\right] & = & D_{\mathrm{KL}}\left[N_{1}:N_{2}\right]. \end{array}$$

This invariance extends to our approximations ${\tilde{\rho }}_{N}^{c}$ (see Equation (27)).

Since we have

$${\tilde{\rho }}_{N}^{c}\left(N_{1},N_{2}\right)\approx \rho_{N}\left(N_{1},N_{2}\right)\geq \rho_{\mathrm{CO}}\left(N_{1},N_{2}\right),$$

the ratio $\kappa_{c}=\frac{{\tilde{\rho }}_{N}^{c}}{\rho_{\mathrm{CO}}}\geq \kappa =\frac{{\tilde{\rho }}_{N}^{c}}{\rho_{N}}$ gives an upper bound on the approximation factor of ${\tilde{\rho }}_{N}^{c}$ compared to the true Fisher–Rao distance $\rho_{N}$:

$$\kappa_{c}\rho_{N}\left(N_{1},N_{2}\right)\geq \kappa \rho_{N}\left(N_{1},N_{2}\right)\geq {\tilde{\rho }}_{N}^{c}\left(N_{1},N_{2}\right)\approx \rho_{N}\left(N_{1},N_{2}\right)\geq \rho_{\mathrm{CO}}\left(N_{1},N_{2}\right).$$

Let us now report some numerical experiments of our approximated Fisher–Rao distances ${\tilde{\rho }}_{N}^{x}$ with x∈{l,m,e,em,CO}. Although that dissimilarity ${\tilde{\rho }}_{N}$ is positive–definite, it does not satisfy the triangular inequality of metric distances (e.g., Riemannian distances $\rho_{N}$ and $\rho_{\mathrm{CO}}$).

First, we draw multivariate normals by sampling means μ∼Unif(0,1) and sample covariance matrices Σ as follows: We draw a lower triangular matrix *L* with entries $L_{ij}$ iid sampled from Unif(0,1), and take $\Sigma =LL^{⊤}$. We use T=1000 samples on curves and repeat the experiment 1000 times to gather average statistics on $\kappa_{c}$’s of curves. Results are summarized in Table 1.

For that scenario that the C&O curve (either ${\bar{c}}_{\mathrm{CO}}\in \bar{N}$ or $c_{\mathrm{CO}}\in N$) performs best compared to the linear interpolation curves with respect to source parameter (*l*), mixture geodesic (*m*), exponential geodesic (*e*), or exponential-mixture mid-curve (em). Let us point out that we sample $\gamma_{P}\left({\bar{P}}_{1},{\bar{P}}_{2};\frac{i}{T}\right)$ for i∈{0,…,T}.

Strapasson, Porto, and Costa [38] (SPC)reported the following upper bound on the Fisher–Rao distance between multivariate normals

$$\rho_{\mathrm{CO}}\left(N_{1},N_{2}\right)\leq \rho_{N}\left(N_{1},N_{2}\right)\leq U_{\mathrm{SPC}}\left(N_{1},N_{2}\right),$$

with:

(35) $$U_{\mathrm{SPC}}\left(N_{1},N_{2}\right)=\sqrt{2\sum_{i=1}^{d}\log^{2}\left(\frac{\sqrt{{\left(1+D_{ii}\right)}^{2}+\mu_{i}^{2}}+\sqrt{{\left(1-D_{ii}\right)}^{2}+\mu_{i}^{2}}}{\sqrt{{\left(1+D_{ii}\right)}^{2}+\mu_{i}^{2}}-\sqrt{{\left(1-D_{ii}\right)}^{2}+\mu_{i}^{2}}}\right)},$$

where $\Sigma =\Sigma_{1}^{-\frac{1}{2}}\Sigma_{2}\Sigma_{1}^{-\frac{1}{2}}$, $\Sigma =\Omega D\Omega^{⊤}$ is the eigen decomposition, and $\mu =\Omega^{⊤}\Sigma_{1}^{-\frac{1}{2}}\left(\mu_{2}-\mu_{1}\right)$. This upper bound performs better when the normals are well-separated and worse than the $\sqrt{D_{J}}$-upper bound when the normals are close to each other.

Let us compare $\rho_{\mathrm{CO}}\left(N_{1},N_{2}\right)$ with $\rho_{N}\left(N_{1},N_{2}\right)\approx {\tilde{\rho }}^{c_{\mathrm{CO}}}\left(N_{1},N_{2}\right)$ and the upper bound $U(N_{1},N_{2})$ by averaging over 1000 trials with $N_{1}$ and $N_{2}$ chosen randomly as before and T=1000. We have $\rho_{\mathrm{CO}}\left(N_{1},N_{2}\right)\leq \rho_{N}\left(N_{1},N_{2}\right)\approx {\tilde{\rho }}^{c_{\mathrm{CO}}}\left(N_{1},N_{2}\right)\leq U\left(N_{1},N_{2}\right)$. Table 2 shows that our Fisher–Rao approximation is close to the lower bound (and hence to the underlying true Fisher–Rao distance) and that the upper bound is about twice the lower bound for that particular scenario.

Second, since the distances are invariant under the action of the affine group, we can set wlog. $N_{1}=\left(0,I\right)$ (standard normal distribution) and let $N_{2}=\mathrm{diag}\left(u_{1},\ldots ,u_{d}\right)$ where $u_{i}∼\mathrm{Unif}\left(0,a\right)$. As normals $N_{1}$ and $N_{2}$ separate from each other, we notice experimentally that the performance of the $c_{\mathrm{CO}}$ curve degrades in the second experiment with a=5 (see Table 3): Indeed, the mixture geodesic works experimentally better than the C&O curve when d≥11.

**Figure 14:**
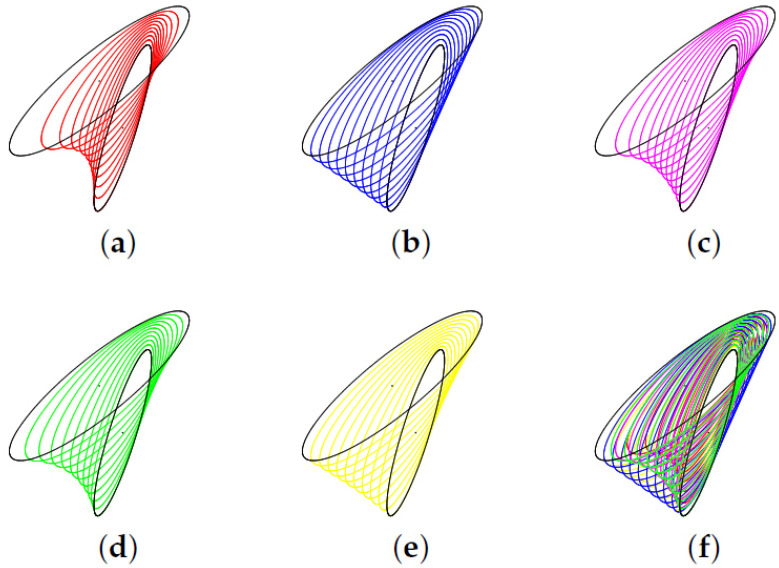
Visualizing at discrete positions (10 increment steps between 0 and 1) some curves used to approximate the Fisher–Rao distance between two bivariate normal distributions: (**a**) exponential geodesic $c^{e}=\gamma_{N}^{e}$ (red), (**b**) mixture geodesic $c^{m}=\gamma_{N}^{m}$ (blue), (**c**) mid-mixture-exponential curve $c^{\mathrm{em}}$ (purple), (**d**) projected Calvo and Oller curve $c^{\mathrm{CO}}$ (green), (**e**) $c^{\lambda }$: ordinary linear interpolation in λ (yellow), and (**f**) All superposed curves at once.

Figure 14 display the various curves considered for approximating the Fisher–Rao distance between bivariate normal distributions: For a curve c(t), we visualize its corresponding bivariate normal distributions $\left(\mu_{c(t)},\Sigma_{c(t)}\right)$ at several increment steps t∈[0,1] by plotting the ellipsoid

$$E_{c(t)}=\mu_{c(t)}+\left\{L^{⊤}x,x=\left(\cos\theta ,\sin\theta \right),\theta \in \left[0,2\pi \right)\right\},$$

where $\Sigma_{c(t)}=L_{c(t)}L_{c(t)}^{⊤}$.

**Example 2.**  *Let us report some numerical results for bivariate normals with T=1000:*

**Figure 15:**
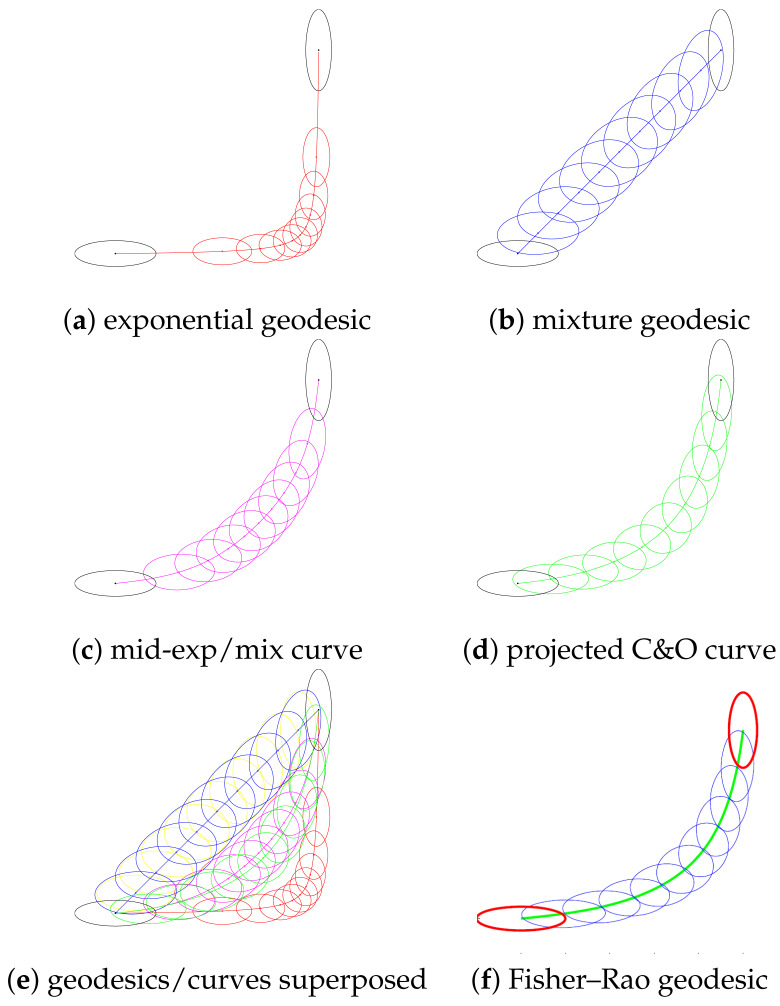
Comparison of our approximation curves with the Fisher–Rao geodesic (**f**) obtained by geodesic shooting (Figure 5 of [39]). Exponential (**a**) and mixture (**b**) geodesics with the mid-exponential-mixture curve (**c**), and the projected C&O curve (**d**). Superposed curves (**e**) and comparison with geodesic shooting (Figure 5 of [39]). Beware that color coding is not related between (**a**) and (**f**), and scale for depicting ellipsoids are different.

**Figure 16:**
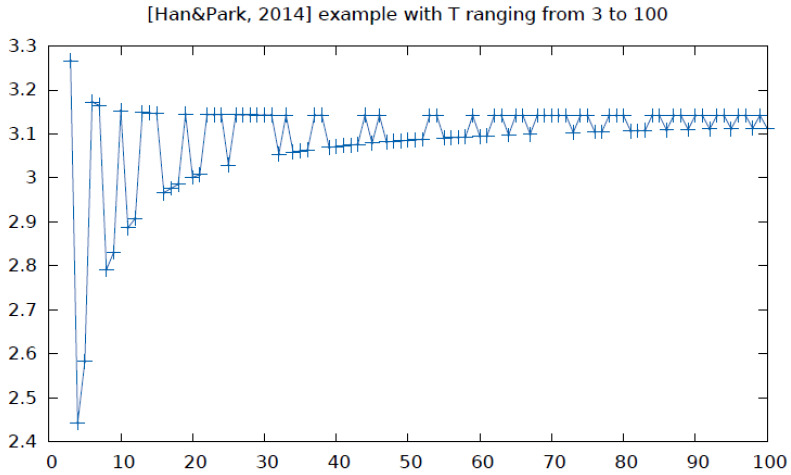
Approximation of the Fisher–Rao distance obtained using the projected C&O curve when *T* ranges from 3 to 100 [39].

- *We use the following example of Han and Park [39] (Equation (26)):*
$$N_{1}=\left(\left[\begin{array}{c} 0\\ 0 \end{array}\right],\left[\begin{array}{cc} 1 & 0\\ 0 & 0.1 \end{array}\right]\right),\;N_{2}=\left(\left[\begin{array}{c} 1\\ 1 \end{array}\right],\left[\begin{array}{cc} 0.1 & 0\\ 0 & 1 \end{array}\right]\right).$$
  *Their geodesic shooting algorithm [39] evaluates the Fisher–Rao distance to $\rho_{N}\left(N_{1},N_{2}\right)\approx 3.1329$ (precision $10^{-5}$).*
*We obtain:*
  – *Calvo and Oller lower bound: $\rho_{\mathrm{CO}}\left(N_{1},N_{2}\right)\approx 3.0470$,*
  – *Upper bound using Equation (15): 7.92179,*
  – *SPC upper bound (Equation (35)): $U_{\mathrm{SPC}}\left(N_{1},N_{2}\right)\approx 5.4302$,*
  – *$\sqrt{D_{J}}$ upper bound: $U_{\sqrt{J}}\left(N_{1},N_{2}\right)\approx 4.3704$,*
  – *${\tilde{\rho }}_{N}^{\lambda }\left(N_{1},N_{2}\right)\approx 3.4496$,*
  – *${\tilde{\rho }}_{N}^{m}\left(N_{1},N_{2}\right)\approx 3.5775$,*
  – *${\tilde{\rho }}_{N}^{e}\left(N_{1},N_{2}\right)\approx 3.7314$,*
  – *${\tilde{\rho }}_{N}^{\mathrm{em}}\left(N_{1},N_{2}\right)\approx 3.1672$,*
  – *${\tilde{\rho }}_{N}^{\mathrm{CO}}\left(N_{1},N_{2}\right)\approx 3.1391$.*
*In that setting, the $\sqrt{D_{J}}$ upper bound is better than the upper bound of Equation (35), and the projected Calvo and Oller geodesic yields the best approximation of the Fisher–Rao distance (Figure 15) with an absolute error of 0.0062 (about 0.2% relative error). When T=10, we have ${\tilde{\rho }}_{N}^{\mathrm{CO}}\left(N_{1},N_{2}\right)\approx 3.1530$, when T=100, we obtain ${\tilde{\rho }}_{N}^{\mathrm{CO}}\left(N_{1},N_{2}\right)\approx 3.1136$, and when T=500 we obtain ${\tilde{\rho }}_{N}^{\mathrm{CO}}\left(N_{1},N_{2}\right)\approx 3.1362$ (which is better than the approximation obtained for T=1000). Figure 16 shows the fluctuations of the approximation of the Fisher–Rao distance by the projected C&O curve when T ranges from 3 to 100.*
- *Bivariate normal $N_{1}=\left(0,I\right)$ and bivariate normal $N_{2}=\left(\mu_{2},\Sigma_{2}\right)$ with $\mu_{2}={\left[1\;0\right]}^{⊤}$ and $\Sigma_{2}=\left[\begin{array}{cc} 1 & -1\\ -1 & 2 \end{array}\right]$. We obtain*
  – *Calvo and Oller lower bound:* **1.4498
**
  – *Upper bound of Equation (35): 2.6072*
  – *$\sqrt{D_{J}}$ upper bound:* **1.5811
**
  – *${\tilde{\rho }}^{\lambda }$: 1.5068*
  – *${\tilde{\rho }}^{m}$: 1.5320*
  – *${\tilde{\rho }}^{e}$: 1.5456*
  – *${\tilde{\rho }}^{\mathrm{em}}$: 1.4681*
  – *${\tilde{\rho }}^{\mathrm{co}}$:* **1.4673
**
- *Bivariate normal $N_{1}=\left(0,I\right)$ and bivariate normal $N_{2}=\left(\mu_{2},\Sigma_{2}\right)$ with $\mu_{2}={\left[5\;0\right]}^{⊤}$ and $\Sigma_{2}=\left[\begin{array}{cc} 1 & -1\\ -1 & 2 \end{array}\right]$. We get:*
  – *Calvo and Oller lower bound:* **3.6852
**
  – *Upper bound of Equation (35):* **6.0392
**
  – *$\sqrt{D_{J}}$ upper bound: 6.2048*
  – *${\tilde{\rho }}^{\lambda }$: 5.7319*
  – *${\tilde{\rho }}^{m}$: 4.4039*
  – *${\tilde{\rho }}^{e}$: 5.9205*
  – *${\tilde{\rho }}^{\mathrm{em}}$:* **4.2901
**
  – *${\tilde{\rho }}^{\mathrm{co}}$: 4.3786*

See Supplementary Materials for further experiments.

## 4. Approximating the Smallest Enclosing Fisher–Rao Ball of MVNs

We may use these closed-form distance $\rho_{\mathrm{CO}}\left(N,N^{\prime }\right)$ between *N* and $N^{\prime }$ to compute an approximation (of the center) of the smallest enclosing Fisher–Rao ball $B^{*}=\mathrm{ball}\left(C^{*},r^{*}\right)$ of a set $G=\{N_{1}=\left(\mu_{1},\Sigma_{1}\right),\ldots ,N_{n}=\left(\mu_{n},\Sigma_{n}\right)\}$ of *nd*-variate normal distributions:

$$C^{*}=\arg\min_{C\in N}\max_{i\in \{1,\ldots ,n\}}\rho_{N}\left(C,N_{i}\right)$$

where $\mathrm{ball}\left(C,r\right)=\{N\in N\;:\;\rho_{N}\left(C,N\right)\leq r\}$.

The method proceeds as follows:

- First, we convert MVN set G into the equivalent set of (d+1)-dimensional SPD matrices $\bar{G}=\left\{{\bar{P}}_{i}=f\left(N_{i}\right)\right\}$ using the C&O embedding. We relax the problem of approximating the circumcenter $C^{*}$ of the smallest enclosing Fisher–Rao ball by
  $$P^{*}=\arg\min_{P\in P(d+1)}\max_{i\in \{1,\ldots ,n\}}\rho_{\mathrm{CO}}\left(P,{\bar{P}}_{i}\right).$$
- Second, we approximate the center of the smallest enclosing Riemannian ball of $\bar{G}$ using the iterative smallest enclosing Riemannian ball algorithm in [66] with say T=1000 iterations. Let $\tilde{P}\in P\left(d+1\right)$ denote this approximation center: $P_{T}={\mathrm{RieSEB}}_{\mathrm{SPD}}\left(\bar{G},T\right)$.
- Finally, we project back $P_{T}$ onto $\bar{N}$: ${\bar{P}}_{T}={\mathrm{proj}}_{\bar{N}}\left(P_{T}\right)$. We return ${\bar{P}}_{T}$ as the approximation of $C^{*}$.

Algorithm [66] ${\mathrm{RieSEB}}_{\mathrm{SPD}}\left(\left\{P_{1},\ldots ,P_{n}\right\},T\right)$ is described for a set of SPD matrices $\left\{P_{1},\ldots ,P_{n}\right\}$ as follows:

- Let $C_{1}\leftarrow P_{1}$
- For t=1 to *T*
  – Compute the index of the SPD matrix which is farthest from the current circumcenter $C_{t}$:
    $$f_{t}=\arg\max_{i\in \{1,\ldots ,n\}}\rho_{\mathrm{SPD}}\left(C_{t},P_{i}\right)$$
  – Update the circumcenter by walking along the geodesic linking $C_{t}$ to $P_{f_{t}}$:
    $$C_{t+1}=\gamma_{\mathrm{SPD}}\left(C_{t},P_{f_{t}};\frac{1}{t+1}\right)=C_{t}^{\frac{1}{2}}{\left(C_{t}^{-\frac{1}{2}}P_{f_{t}}C_{t}^{-\frac{1}{2}}\right)}^{\frac{1}{t+1}}C_{t}^{\frac{1}{2}}$$
- Return $C_{T}$

The convergence of the algorithm ${\mathrm{RieSEB}}_{\mathrm{SPD}}$ follows from the fact that the SPD trace manifold is a Hadamard manifold (with negative sectional curvatures). See [66] for proof of convergence.

**Figure 17:**
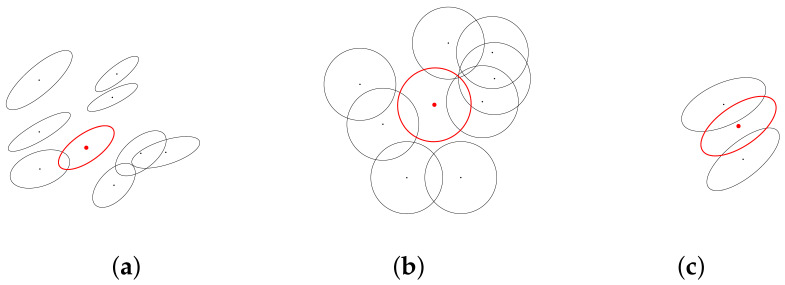
Approximation of the smallest enclosing Riemannian ball of a set of *n* bivariate normals $N_{i}=N\left(\mu_{i},\Sigma_{i}\right)$ with respect to C&O distance $\rho_{\mathrm{CO}}$ (the approximate circumcenter ${\bar{C}}_{T}$ is depicted as a red ellipse): (**a**) n=8 with different covariance matrices, (**b**) n=8 with identical covariance matrices amount to the smallest enclosing ball of a set of *n* points $\left\{\mu_{i}\right\}$, (**c**) n=2 displays the midpoint of the C&O geodesic visualized as an equivalent bivariate normal distribution in the sample space.

The SPD distance $\rho_{P}\left(C_{T},{\bar{C}}_{T}\right)$ indicates the quality of the approximation. Figure 17 shows the result of implementing this heuristic.

Let us notice that when all MVNs share the same covariance matrix Σ, we have from Equation (18) or Equation (23) that $\rho_{N}\left(\mu_{1},\Sigma \right),N\left(\mu_{2},\Sigma \right)$ and $\rho_{\mathrm{CO}}\left(N\left(\mu_{1},\Sigma \right),N\left(\mu_{2},\Sigma \right)\right)$ are strictly increasing function of their Mahalanobis distance. Using the Cholesky decomposition $\Sigma^{-1}=LL^{⊤}$, we deduce that the smallest Fisher–Rao enclosing ball coincides with the smallest Calvo and Oller enclosing ball, and the circumcenter of that ball can be found as an ordinary Euclidean circumcenter [83] (Figure 17b). Please note that in 1D, we can find the exact smallest enclosing Fisher–Rao ball as an equivalent smallest enclosing ball in hyperbolic geometry.

Furthermore, we may extend the computation of the approximated circumcenter to *k*-center clustering [84] of *n* multivariate normal distributions. Since the circumcenter of the clusters is approximated and not exact, we extend straightforwardly the variational approach of *k*-means described in [85] to *k*-center clustering. An application of *k*-center clustering of MVNs is to simplify a Gaussian mixture model [42] (GMM).

Similarly, we can consider other Riemannian distances with closed-form formulas between MVNs such as the Killing distance in the symmetric space [82] (see Appendix C) or the Siegel-based distance proposed in Appendix D.

## 5. Some Information–Geometric Properties of the C&O Embedding

In information geometry [1], the manifold N admits a dual structure denoted by the quadruple

$$(N,g_{N}^{\mathrm{Fisher}},\nabla_{N}^{e},\nabla_{N}^{m}),$$

when equipped with the exponential connection $\nabla_{N}^{e}$ and the mixture connection $\nabla_{N}^{m}$. The connections $\nabla_{N}^{e}$ and $\nabla_{N}^{m}$ are said to be dual since $\frac{\nabla_{N}^{e}+\nabla_{N}^{m}}{2}={\bar{\nabla }}_{N}$, the Levi–Civita connection induced by $g_{N}^{\mathrm{Fisher}}$. Furthermore, by viewing N as an exponential family $\left\{p_{\theta }\right\}$ with natural parameter $\theta =(\theta_{v},\theta_{M})$ (using the sufficient statistics [80]$\left(x,-xx^{⊤}\right)$), and taking the convex log-normalizer function $F_{N}\left(\theta \right)$ of the normals, we can build a dually flat space [1] where the canonical divergence amounts to a Bregman divergence which coincides with the reverse Kullback–Leibler divergence [30,86] (KLD). The Legendre duality

$$F^{*}\left(\eta \right)=\langle \nabla F(\theta ),\eta \rangle -F\left(\nabla F\left(\theta \right)\right)$$

(with $\langle \left(v_{1},M_{1}\right),\left(v_{2},M_{2}\right)\rangle =\mathrm{tr}\left(v_{1}v_{2}^{⊤}+M_{1}M_{2}^{⊤}\right)=v_{1}\cdot v_{2}+\mathrm{tr}\left(M_{1}M_{2}^{⊤}\right)$) yields: $\theta =\left(\theta_{v},\theta_{M}\right)=\left(\Sigma^{-1}\mu ,\frac{1}{2}\Sigma^{-1}\right)$,

$$F_{N}\left(\theta \right)=\frac{1}{2}\left(d\log\pi -\log|\theta_{M}|+\frac{1}{2}\theta_{v}^{⊤}\theta_{M}^{-1}\theta_{v}\right),$$

$\eta =\left(\eta_{v},\eta_{M}\right)=\nabla F_{N}\left(\theta \right)=\left(\frac{1}{2}\theta_{M}^{-1}\theta_{v},\theta_{M}^{-1}\right)$,

$$F_{N}^{*}\left(\eta \right)=-\frac{1}{2}\left(\log\left(1+\eta_{v}^{⊤}\eta_{M}^{-1}\eta_{v}\right)+\log\left|-\eta_{M}\right|+d\left(\log2\pi e\right)\right),$$

and we have

$$B_{F_{N}}\left(\theta_{1},\theta_{2}\right)=D_{\mathrm{KL}}^{*}\left(p_{\lambda_{1}}:p_{\lambda_{2}}\right)=D_{\mathrm{KL}}\left(p_{\lambda_{2}}:p_{\lambda_{1}}\right)=B_{F_{N}^{*}}\left(\eta_{2}:\eta_{1}\right),$$

where $D_{\mathrm{KL}}^{*}\left[p:q\right]=D_{\mathrm{KL}}\left[q:p\right]$ is the reverse KLD.

In a dually flat space, we can express the canonical divergence as a Fenchel–Young divergence using the mixed coordinate systems $B_{F_{N}}\left(\theta_{1}:\theta_{2}\right)=Y_{F_{N}}\left(\theta_{1}:\eta_{2}\right)$ where $\eta_{i}=\nabla F_{N}\left(\theta_{i}\right)$ and

$$Y_{F_{N}}\left(\theta_{1}:\eta_{2}\right):=F_{N}\left(\theta_{1}\right)+F_{N}^{*}\left(\eta_{2}\right)-\langle \theta_{1},\eta_{2}\rangle .$$

The moment η-parameterization of a normal is $\left(\eta =\mu ,H=-\Sigma -\mu \mu^{⊤}\right)$ with its reciprocal function $\left(\lambda =\eta ,\Lambda =-H-\eta \eta^{⊤}\right)$.

Let $F_{P}\left(P\right)=F_{N}\left(0,P\right)$, $\bar{\theta }=\frac{1}{2}{\bar{P}}^{-1}$, $\bar{\eta }=\nabla F_{P}\left(\bar{\theta }\right)$. Then we have the following proposition which proves that the Fenchel–Young divergences in N and $\bar{N}$ (as a submanifold of P) coincide:

**Proposition 4.**  *We have*

$$\begin{array}{ccc} D_{\mathrm{KL}}\left[p_{\mu_{1},\Sigma_{1}}:p_{\mu_{2},\Sigma_{2}}\right] & = & B_{F_{N}}\left(\theta_{2}:\theta_{1}\right)=Y_{F_{N}}\left(\theta_{2}:\eta_{1}\right)=Y_{F_{P}}\left({\bar{\theta }}_{2}:{\bar{\eta }}_{1}\right)\\ & = & B_{F_{P}}\left({\bar{\theta }}_{2}:{\bar{\theta }}_{1}\right)=D_{\mathrm{KL}}\left[p_{0,{\bar{P}}_{1}=f\left(\mu_{1},\Sigma_{2}\right)}:p_{0,{\bar{P}}_{2}=f\left(\mu_{2},\Sigma_{2}\right)}\right]. \end{array}$$

Consider now the $\nabla^{e}$-geodesics and $\nabla^{m}$-geodesics on N (linear interpolation with respect to natural and dual moment parameterizations, respectively): $\gamma_{N}^{e}\left(N_{1},N_{2};t\right)=\left(\mu_{t}^{e},\Sigma_{t}^{e}\right)$ and $\gamma_{N}^{m}\left(N_{1},N_{2};t\right)=\left(\mu_{t}^{m},\Sigma_{t}^{m}\right)$.

**Proposition 5**  (Mixture geodesics preserved)**.** *The mixture geodesics are preserved by the embedding f: $f\left(\gamma_{N}^{m}\left(N_{1},N_{2};t\right)\right)=\gamma_{P}^{m}\left(f\left(N_{1}\right),f\left(N_{2}\right);t\right)$. The exponential geodesics are preserved for the subspace of N with fixed mean μ: $N_{\mu }$.*

**Proof.**  For the *m*-geodesics, let us check that

$$f\left(\mu_{t}^{m},\Sigma_{t}^{m}\right)=\left[\begin{array}{cc} \Sigma_{t}^{m}+\mu_{t}^{m}{\mu_{t}^{m}}^{⊤} & \mu_{t}^{m}\\ {\left(\mu_{t}^{m}\right)}^{⊤} & 1 \end{array}\right]=t\underset{{\bar{P}}_{1}}{\underbrace{f(\mu_{1},\Sigma_{1})}}+\left(1-t\right)\underset{{\bar{P}}_{2}}{\underbrace{f(\mu_{2},\Sigma_{2})}},$$

since $\Sigma_{t}^{m}+\mu_{t}{\mu_{t}^{m}}^{⊤}={\bar{\Sigma }}_{t}+t\mu_{1}\mu_{1}^{⊤}+\left(1-t\right)\mu_{2}\mu_{2}^{⊤}$$=t\left(\Sigma_{1}+\mu_{1}\mu_{1}^{⊤}\right)+\left(1-t\right)\left(\Sigma_{2}+\mu_{2}\mu_{2}^{⊤}\right)$. Thus, we have $f\left(\gamma_{N}^{m}\left(N_{1},N_{2};t\right)\right)=\gamma_{P}^{m}\left({\bar{P}}_{1},{\bar{P}}_{2};t\right)$. □

Therefore, all algorithms on N which only require *m*-geodesics or *m*-projections [1] by minimizing the right-hand side of the KLD can be implemented by algorithms on P. See, for example, the minimum enclosing ball approximation algorithm called BBC in [87]. Notice that ${\bar{N}}_{\mu }$ (fixed mean normal submanifolds) preserve both mixture and exponential geodesics: The submanifolds ${\bar{N}}_{\mu }$ are said to be doubly autoparallel [88].

**Remark 5.**  *In [2] (p. 355), exercises 13.8 and 13.9 ask to prove the equivalence of the following statements for S a submanifold of M:*

- *S is an exponential family ⇔S is $\nabla^{1}$-autoparallel in M (exercise 13.8),*
- *S is a mixture family ⇔S is $\nabla^{-1}$-autoparallel in M (exercise 13.9).*

*Let $\bar{P}=\left[\begin{array}{cc} \Sigma +\mu \mu^{⊤} & \mu \\ \mu^{⊤} & 1 \end{array}\right]$ (with $|\bar{P}\left|=|\Sigma \right|$), ${\bar{P}}^{-1}=\left[\begin{array}{cc} \Sigma^{-1} & -\Sigma^{-1}\mu \\ -\mu^{⊤}\Sigma^{-1} & 1+\mu^{⊤}\Sigma^{-1}\mu \end{array}\right]$, and y=(x,1). Then we have*

$$\begin{array}{ccc} q_{\bar{P}}\left(y\right) & = & \frac{1}{{\left(2\pi \right)}^{\frac{d+1}{2}}\sqrt{|\bar{P}|}}\exp\left(-\frac{1}{2}y^{⊤}{\bar{P}}^{-1}y\right),\\ & = & \frac{1}{{\left(2\pi \right)}^{\frac{d+1}{2}}\sqrt{\left|\Sigma \right|}}\exp\left(-\frac{1}{2}y^{⊤}{\bar{P}}^{-1}y\right),\\ & = & \frac{1}{{\left(2\pi \right)}^{\frac{d+1}{2}}\sqrt{\left|\Sigma \right|}}\exp\left(\left[x^{⊤}\;1\right]\left[\begin{array}{cc} \Sigma^{-1} & -\Sigma^{-1}\mu \\ -\mu^{⊤}\Sigma^{-1} & 1+\mu^{⊤}\Sigma^{-1}\mu \end{array}\right]\left[\begin{array}{c} x\\ 1 \end{array}\right]\right). \end{array}$$

*Thus, $\bar{N}=\left\{q_{\bar{P}}\left(x,1\right)\right\}$ is an exponential family. Therefore, we deduce that P is $\nabla^{e}$-autoparallel in P. However, $\bar{N}$ is not a mixture family and thus P is not $\nabla^{m}$-autoparallel in P.*

## 6. Conclusions and Discussion

In general, the Fisher–Rao distance between multivariate normals (MVNs) is not known in closed form. In practice, the Fisher–Rao distance is usually approximated by costly geodesic shooting techniques [39,40,41] which requires time-consuming computations of the Riemannian exponential map and are nevertheless limited to normals within a short range of each other. In this work, we consider a simple alternative approach for approximating the Fisher–Rao distance by approximating the Riemannian lengths of curves, which admits closed-form parameterizations. In particular, we considered the mixed exponential-mixture curved and the projected symmetric positive–definite matrix geodesic obtained from Calvo and Oller isometric submanifold embedding into the SPD cone [19]. We summarize our method to approximate $\rho_{N}\left(N_{1},N_{2}\right)$ between $N_{1}=N\left(\mu_{1},\Sigma_{1}\right)$ and $N_{2}=N\left(\mu_{2},\Sigma_{2}\right)$ as follows:

$${\tilde{\rho }}_{T}^{\mathrm{CO}}\left(N_{1},N_{2}\right):=\frac{1}{T}\sum_{i=1}^{T-1}\sqrt{D_{J}\left[{\bar{S}}_{t},{\bar{S}}_{t+1}\right]},$$

where

$${\bar{S}}_{t}={\mathrm{proj}}_{\bar{N}}\left(S_{t}\right),\;{\mathrm{proj}}_{\bar{N}}\left(\left[\begin{array}{cc} \Sigma +\beta \mu \mu^{⊤} & \beta \mu \\ \beta \mu^{⊤} & \beta \end{array}\right]\right)=\left[\begin{array}{cc} \Sigma +\mu \mu^{⊤} & \mu^{⊤}\\ \mu & 1 \end{array}\right]$$

and

$$S_{t}={\bar{P}}_{1}^{\frac{1}{2}}\;{\left({\bar{P}}_{1}^{-\frac{1}{2}}{\bar{P}}_{2}^{\frac{1}{2}}{\bar{P}}_{1}^{-\frac{1}{2}}\right)}^{\frac{t}{T}}\;{\bar{P}}_{1}^{\frac{1}{2}}$$

with

$${\bar{P}}_{1}=f\left(N_{1}\right)=\left[\begin{array}{cc} \Sigma_{1}+\mu_{1}\mu_{1}^{⊤} & \mu_{1}\\ \mu_{1}^{⊤} & 1 \end{array}\right],\;{\bar{P}}_{2}=f\left(N_{2}\right)=\left[\begin{array}{cc} \Sigma_{2}+\mu_{2}\mu_{2}^{⊤} & \mu_{2}\\ \mu_{2}^{⊤} & 1 \end{array}\right].$$

We proved the following sandwich bounds of our approximation

$$\rho_{N}\left(N_{1},N_{2}\right)\leq {\tilde{\rho }}_{T}^{\mathrm{CO}}\left(N_{1},N_{2}\right)\leq \rho_{N}\left(N_{1},N_{2}\right)+2\delta_{T}^{\mathrm{CO}}\left({\bar{P}}_{1},{\bar{P}}_{2}\right),$$

where

$$\delta_{T}^{\mathrm{CO}}\left(P_{1},P_{2}\right):=\frac{1}{T}\sum_{i=1}^{T}\rho_{P}\left(S_{t},{\bar{S}}_{t}\right).$$

Notice that we may calculate equivalently $D_{J}\left[{\bar{S}}_{t},{\bar{S}}_{t+1}\right]$ as $D_{J}\left[G_{t},G_{t+1}\right]$ where $G_{i}=f^{-1}\left({\bar{S}}_{i}\right)=N\left(m_{i},C_{i}\right)$ for i∈{0,…,T} (see Proposition 3).

We also reported a fast way to upper bound the Fisher–Rao distance by the square root of Jeffreys’ divergence: $\rho_{N}\left(N_{1},N_{2}\right)\leq \sqrt{D_{J}\left[N_{1},N_{2}\right]}$ which is tight at infinitesimal scale. In practice, this upper bound beats the upper bound of [38] when normal distributions are not too far from each other. Finally, we show that not only is Calvo and Oller SPD submanifold embedding [19] isometric, but it also preserves the Kullback–Leibler divergence, the Fenchel–Young divergence, and the mixture geodesics. Our approximation technique extends to elliptical distribution, which generalizes multivariate normal distributions [32,55]. Moreover, we obtained a closed form for the Fisher–Rao distance between normals sharing the same covariance matrix using the technique of maximal invariance under the action of the affine group in Section 1.5. We may also consider other distances different from the Fisher–Rao distance, which admits a closed-form formula: For example, the Calvo and Oller metric distance [19] (a lower bound on the Fisher–Rao distance) or the metric distance proposed in [82] (see Appendix C) whose geodesics enjoys the asymptotic property of the Fisher–Rao geodesics [89]). The C&O distance is very well-suited for short Fisher–Rao distances while the symmetric space distance is well-tailored for large Fisher–Rao distances. The calculations of these closed-form distances rely on generalized eigenvalues. We also propose an embedding of normals into the Siegel upper space in Appendix D. To conclude, let us propose yet another alternative distance, The Hilbert projective distance on the SPD cone [90], which only needs to calculate the minimal and maximal eigenvalues (say, using the power iteration method [91]):

(36) $$\rho_{\mathrm{Hilbert}}\left(P_{1},P_{2}\right)=\log\left(\frac{\lambda_{\max}\left(P_{1}^{-1}P_{2}\right)}{\lambda_{\min}\left(P_{1}^{-1}P_{2}\right)}\right).$$

The dissimilarity is said projective on the SPD cone because $\rho_{\mathrm{Hilbert}}\left(P_{1},P_{2}\right)=0$ if and only if $P_{1}=\lambda P_{2}$ for some λ>0. However, let us notice that it yields a proper metric distance on $\bar{N}$:

$$\rho_{\mathrm{Hilbert}}\left(N_{1},N_{2}\right):=\rho_{\mathrm{Hilbert}}\left({\bar{P}}_{1},{\bar{P}}_{2}\right),$$

since ${\bar{P}}_{1}=\lambda {\bar{P}}_{2}$ if and only if λ=1 because the array element ${\left(P_{1}\right)}_{d+1,d+1}={\left(P_{2}\right)}_{d+1,d+1}=1$, i.e., ${\bar{P}}_{1}={\bar{P}}_{2}$ implying $P_{1}=P_{2}$ by the isometric diffeomorphism *f*.

Notice that since $\lambda_{\max}\left(P\right)=\lambda_{\min}\left(P^{-1}\right)$, $\lambda_{\min}\left(P\right)=\lambda_{\max}\left(P^{-1}\right)$,

$\lambda_{\max}\left(P_{1}P_{2}\right)\leq \lambda_{\max}\left(P_{1}\right)\lambda_{\max}\left(P_{2}\right)$, and $\lambda_{\min}\left(P_{1}P_{2}\right)\geq \lambda_{\min}\left(P_{1}\right)\lambda_{\min}\left(P_{2}\right)$, we have the following upper bound on Hilbert distance: $\rho_{\mathrm{Hilbert}}\left(P_{1},P_{2}\right)\leq \log\frac{\lambda_{\max}\left(P_{1}\right)}{\lambda_{\min}\left(P_{1}\right)}+\log\frac{\lambda_{\max}\left(P_{2}\right)}{\lambda_{\min}\left(P_{2}\right)}$.

## Figures and Tables

**Table 1:** First set of experiments demonstrates the advantage of the $c_{\mathrm{CO}}\left(t\right)$ curve.

*d*	$\kappa_{\mathrm{CO}}$	$\kappa_{l}$	$\kappa_{e}$	$\kappa_{m}$	$\kappa_{\mathrm{em}}$
1	**1.0025**	1.0414	1.1521	1.0236	1.0154
2	**1.0167**	1.0841	1.1923	1.0631	1.0416
3	**1.0182**	1.8997	2.6072	1.9965	1.07988
4	**1.0207**	2.0793	1.8080	2.1687	1.1873
5	**1.0324**	4.1207	12.3804	5.6170	4.2349

**Table 2:** Comparing our Fisher–Rao approximation with the Calvo and Oller lower bound and the upper bound of [38].

*d*	$\rho_{\mathrm{CO}}\left(N_{1},N_{2}\right)$	${\tilde{\rho }}^{c_{\mathrm{CO}}}\left(N_{1},N_{2}\right)$	$U(N_{1},N_{2})$
1	1.7563	1.8020	3.1654
2	3.2213	3.3194	6.012
3	4.6022	4.7642	8.7204
4	5.9517	6.1927	11.3990
5	7.156	7.3866	13.8774

**Table 3:** Second set of experiments shows limitations of the $c_{\mathrm{CO}}\left(t\right)$ curve.

*d*	$\kappa_{\mathrm{CO}}$	$\kappa_{l}$	$\kappa_{e}$	$\kappa_{m}$
1	**1.0569**	1.1405	1.139	1.0734
5	**1.1599**	1.4696	1.5201	1.1819
10	**1.2180**	1.6963	1.7887	1.2184
11	1.2260	1.7333	1.8285	**1.2235**
12	1.2301	1.7568	1.8539	**1.2282**
15	1.2484	1.8403	1.9557	**1.2367**
20	1.2707	1.9519	2.0851	**1.2466**

**Figure A1:**
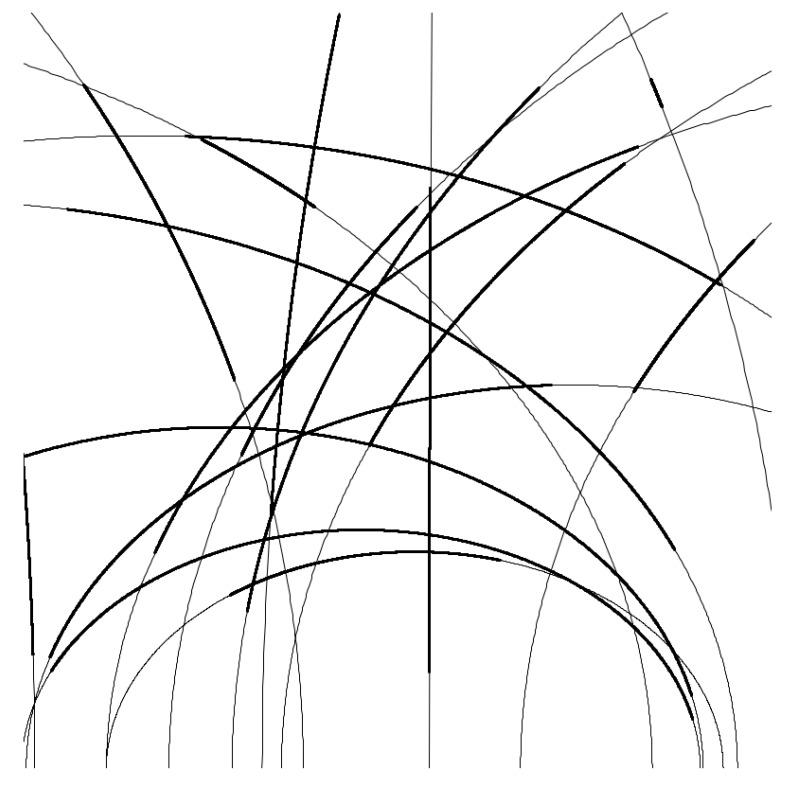
Visualizing some Fisher–Rao geodesics of univariate normal distributions on the stretched Poincaré upper plane (semi-circles with origin on the *x*-axis and stretched by $\sqrt{2}$ on the *x*-axis). Full geodesics are plotted with a thin gray style and geodesic arcs are plotted with a thick black style.

**Figure A2:**
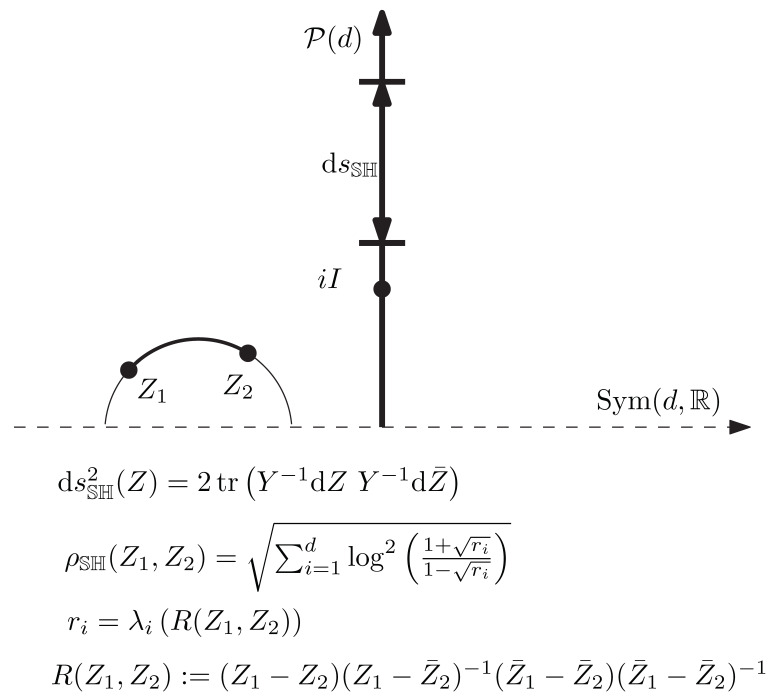
Siegel upper space generalizes the Poincaré hyperbolic upper plane.

## Supplementary Materials

The following supporting information can be downloaded at: https://franknielsen.github.io/FisherRaoMVN.

## Abbreviations

Entities	
N(μ,Σ)	*d*-variate normal distribution (mean μ, covariance matrix Σ)
$p_{\left(\mu ,\Sigma \right)}\left(x\right)$	Probability density function of N(μ,Σ)
$q_{\Sigma }\left(y\right)=p_{\left(0,\Sigma \right)}\left(y\right)$	Probability density function of N(0,Σ)
$P=(P_{ij})$	Positive–definite matrix with matrix entries $P_{ij}$
Mappings	
$\bar{P}=f_{1}\left(N\right)$	Calvo and Oller mapping [19] (1990)
$\hat{P}=f_{-\frac{1}{d+1},1}\left(N\right)=\hat{f}\left(N\right)$	Calvo and Oller mapping [32] (2002) or [82]
Groups	
GL(d)	Group of linear transformations (invertible d×d matrices)
SL(d)	Special linear group (d×d matrices with unit determinant)
Aff(d)	Affine group of dimension *d*
Sets	
N	Set of multivariate normal distributions N(μ,Σ) (MVNs)
Sym(d)	Set of symmetric d×d real matrices
P	Symmetric positive–definite matrix cone (SPD matrix cone)
$P_{c}$	Set of SPD matrices with fixed determinant *c* ($P=R_{>0}\times P_{c}$)
SSPD, $P_{1}$	Set of SPD matrices with unit determinant
Λ	Parameter space of N(μ,Σ): $R^{d}\times P\left(d\right)$
$N_{0}$, P	Set of zero-centered normal distributions N(0,Σ)
$N_{\Sigma }$	Set of normal distributions N(μ,Σ) with fixed Σ
$N_{\mu }$	Set of normal distributions N(μ,Σ) with fixed μ
$\bar{N}$	Set of SPD matrices f(N)
Riemannian length elements	
MVN Fisher	$ds_{\mathrm{Fisher},N}^{2}=d\mu^{⊤}\Sigma^{-1}d\mu +\frac{1}{2}\mathrm{tr}\left({\left(\Sigma^{-1}d\Sigma \right)}^{2}\right)$
0-MVN Fisher	$ds_{\mathrm{Fisher},N_{0}}^{2}=\frac{1}{2}\mathrm{tr}\left({\left(\Sigma^{-1}d\Sigma \right)}^{2}\right)$
SPD trace	$ds_{\beta ,\mathrm{trace}}^{2}=\beta \mathrm{tr}\left({\left(P\;dP\right)}^{2}\right)$ (when $\beta =\frac{1}{2}$, $ds_{\mathrm{trace}}=ds_{\mathrm{Fisher},N_{0}}$)

SPD Calvo and Oller metric	$ds_{\mathrm{CO}}^{2}=\frac{1}{2}{\left(\frac{d\beta }{\beta }\right)}^{2}+\beta d\mu^{⊤}\Sigma^{-1}d\mu +\frac{1}{2}\mathrm{tr}\left({\left(\Sigma^{-1}d\Sigma \right)}^{2}\right)$
	(with $ds_{\mathrm{CO}}=ds_{P}\left(f\left(\mu ,\Sigma \right)\right)$)
	when β=1, $ds_{\mathrm{CO}}=ds_{\mathrm{Fisher},N}$ in $\bar{N}$
SPD symmetric space	$ds_{\mathrm{SS}}^{2}=\frac{1}{2}d\mu^{⊤}\Sigma^{-1}d\mu +\mathrm{tr}\left({\left(\Sigma^{-1}d\Sigma \right)}^{2}\right)-\frac{1}{2}{\mathrm{tr}}^{2}\left(\Sigma^{-1}d\Sigma \right)$
Siegel upper space	$ds_{\mathrm{SH}}^{2}\left(Z\right)=2\mathrm{tr}\left(Y^{-1}dZ\;Y^{-1}d\bar{Z}\right)$ ($ds_{\mathrm{SH}}\left(iY\right)=2ds_{\mathrm{Fisher},N_{0}}$)
Manifolds and submanifolds	
M ($=M_{N}$)	Manifold of multivariate normal distributions
$T_{p}M$	Tangent space at p∈M
$S_{\mu }\subset M$	Submanifold of MVNs with μ prescribed
$S_{\Sigma }\subset M$	Submanifold of MVNs with Σ prescribed
$M_{\Sigma }$	manifold of $N_{\Sigma }$ (non-embedded in M)
$M_{\mu }$	manifold of $N_{\mu }$ (non-embedded in M)
$S_{[v],\Sigma }$	Submanifold of MVN set {N(λv,Σ):λ>0}
	where *v* is an eigenvector of Σ
P	manifold of symmetric positive–definite matrices
Distances	
$\rho_{N}\left(N_{1},N_{2}\right)$	Fisher–Rao distance between normal distributions $N_{1}$ and $N_{2}$
$\rho_{\mathrm{SPD}}\left(P_{1},P_{2}\right)$	Riemannian SPD distance between $P_{1}$ and $P_{2}$
$\rho_{\mathrm{CO}}\left(N_{1},N_{2}\right)$	Calvo and Oller distance from embedding *N* to $\bar{P}=f\left(N\right)$
$\rho_{\mathrm{SS}}\left(N_{1},N_{2}\right)$	Symmetric space distance from embedding *N* to $\hat{P}=\hat{f}\left(N\right)$
$\rho_{\mathrm{Hilbert}}\left(N_{1},N_{2}\right)$	Hilbert distance $\rho_{\mathrm{Hilbert}}\left({\bar{P}}_{1},{\bar{P}}_{2}\right)$
$D_{\mathrm{KL}}\left(N_{1},N_{2}\right)$	Kullback–Leibler divergence between MVNs $N_{1}$ and $N_{2}$
$D_{J}\left(N_{1},N_{2}\right)$	Jeffreys divergence between MVNs $N_{1}$ and $N_{2}$
$D_{\mathrm{CO}}\left(N_{1},N_{2}\right)$	Calvo and Oller dissimilarity measure of Equation (26)
Geodesics and curves	
$\gamma_{N}^{\mathrm{FR}}\left(N_{1},N_{2};t\right)$	Fisher–Rao geodesic between MVNs $N_{1}$ and $N_{2}$
$\gamma_{P}^{\mathrm{FR}}\left(P_{1},P_{2};t\right)$	Fisher–Rao geodesic between SPD $P_{1}$ and $P_{2}$
$\gamma_{N}^{e}\left(N_{1},N_{2};t\right)$	exponential geodesic between MVNs $N_{1}$ and $N_{2}$
$\gamma_{N}^{m}\left(N_{1},N_{2};t\right)$	mixture geodesic between MVNs $N_{1}$ and $N_{2}$
$\gamma_{N}^{\mathrm{CO}}\left(N_{1},N_{2};t\right)$	projection curve (not geodesic) of $\gamma_{P}\left({\bar{P}}_{1},{\bar{P}}_{2};t\right)$ onto $\bar{N}$
Metrics and connections	
$g_{N}^{\mathrm{Fisher}}$	Fisher information metric of MVNs
$g_{P}$	trace metric
$g_{P}$	Fisher information metric of centered MVNs
$g_{\mathrm{Killing}}$	Killing metric studied in [82]
$\nabla_{N}^{\mathrm{Fisher}}$	Levi–Civita metric connection
$\nabla_{N}^{e}$	exponential connection
$\nabla_{N}^{m}$	mixture connection

## Appendix A. Geodesics on the Fisher–Rao Normal Manifold

### Appendix A.1. Parametric Equations of the Fisher–Rao Geodesics between Univariate Normal Distributions

The Fisher–Rao geodesics $\gamma_{N}^{\mathrm{FR}}\left(N_{1},N_{2}\right)$ on the Fisher–Rao univariate normal manifolds are either vertical line segments when $\mu_{1}=\mu_{2}$, or semi-circle with origin on the *x*-axis and *x*-axis stretched by $\sqrt{2}$ [92] (Figure A1):

$$\gamma_{N}^{\mathrm{FR}}\left(\mu_{1},\sigma_{1};\mu_{2},\sigma_{2}\right)=\left\{\begin{array}{cc} (\mu ,\left(1-t\right)\sigma_{1}+t\sigma_{2}), & \mu_{1}=\mu_{2}=\mu \\ (\sqrt{2}\left(c+r\cos t,r\sin t\right),t\in \left[\min\left\{\theta_{1},\theta_{2}\right\},\max\left\{\theta_{1},\theta_{2}\right\}\right], & \mu_{1}\neq \mu_{2}, \end{array}\right.,$$

where

$$c=\frac{\frac{1}{2}\left(\mu_{2}^{2}-\mu_{1}^{2}\right)+\sigma_{2}^{2}-\sigma_{1}^{2}}{\sqrt{2}\left(\mu_{1}-\mu_{2}\right)},\;r=\sqrt{{\left(\frac{\mu_{i}}{\sqrt{2}}-c\right)}^{2}+\sigma_{i}^{2}},i\in \left\{1,2\right\},$$

and

$$\theta_{i}=\arctan\left(\frac{\sigma_{i}}{\frac{\mu_{i}}{\sqrt{2}}-c}\right),i\in \left\{1,2\right\},$$

provided that $\theta_{i}\geq 0$ for i∈{1,2} (otherwise, we let $\theta_{i}\leftarrow \theta_{i}+\pi$).

Notice that it is remarkable that the Fisher–Rao distance between normal distributions is available in closed form: Indeed, the Euclidean length (with respect to the Euclidean metric) of semi-ellipse curves (perimeters) is not known in closed form but can be expressed using the so-called complete elliptic integral of the second kind [93].

### Appendix A.2. Geodesics with Initial Values on the Multivariate Fisher–Rao Normal Manifold

The geodesic equation is given by

$$\left\{\begin{array}{ccc} \ddot{\mu }-\dot{\Sigma }\Sigma^{-1}\dot{\mu } & = & 0,\\ \ddot{\Sigma }+\dot{\mu }{\dot{\mu }}^{⊤}-\dot{\Sigma }\Sigma^{-1}\dot{\Sigma } & = & 0. \end{array}\right.$$

We concisely report the parametric geodesics using another variant of the natural parameters of the normal distributions (slightly differing from the θ-coordinate system since natural parameters can be chosen up to a fixed affine transformation by changing accordingly the sufficient statistics by the inverse affine transformation) viewed as an exponential family:

$$\left(\xi =\Sigma^{-1}\mu ,\Xi =\Sigma^{-1}\right).$$

In general, the geodesics with boundary values $\gamma_{N}^{\mathrm{Fisher}}\left(N_{1},N_{2};t\right)$ are not known in closed form. However, Calvo and Oller [48] (Theorem 3.1 and Corollary 1) reported the explicit equations of the geodesics when the initial values are given, i.e., $\gamma_{N}^{\mathrm{Fisher}}\left(N_{0},v_{0};t\right)$ where $v_{0}={\dot{\gamma }}_{N}^{\mathrm{Fisher}}\left(N_{0},v_{0};0\right)=\left(\dot{\xi }\left(0\right),\dot{\Xi }\left(0\right)\right)$ is in $T_{N_{0}}M$ and $\gamma_{N}^{\mathrm{Fisher}}\left(N_{0},v_{0};0\right)=N_{0}$.

Let

$$\begin{array}{ccc} B & = & -\Xi {\left(0\right)}^{-\frac{1}{2}}\;\dot{\Xi }\left(0\right)\;\Xi {\left(0\right)}^{-\frac{1}{2}},\\ a & = & \Xi {\left(0\right)}^{-\frac{1}{2}}\dot{\xi }\left(0\right)+B\Xi_{0}^{-\frac{1}{2}}\xi \left(0\right),\\ G & = & {\left(B^{2}+2aa^{⊤}\right)}^{\frac{1}{2}}, \end{array}$$

and $G^{†}$ be the Moore–Penrose generalized inverse matrix of *G*: $G^{†}={\left(G^{⊤}G\right)}^{-1}G^{⊤}$ or $G^{†}=G^{⊤}{\left(GG^{⊤}\right)}^{-1}$. The Moore–Penrose pseudo-inverse matrix can be replaced by any other pseudo-inverse matrix $G^{-}$ [48].

Then we have $\left(\xi \left(t\right),\Xi \left(t\right)\right)=\gamma_{N}^{\mathrm{Fisher}}\left(N_{0},v_{0};t\right)$ with

$$\begin{array}{c} R\left(t\right)=\mathrm{Cosh}\left(\frac{1}{2}Gt\right)-BG^{†}\mathrm{Sinh}\left(\frac{1}{2}Gt\right),\\ \Xi \left(t\right)=\Xi {\left(0\right)}^{\frac{1}{2}}\;R\left(t\right)R{\left(t\right)}^{⊤}\;\Xi {\left(0\right)}^{\frac{1}{2}},\\ \xi \left(t\right)=2\Xi {\left(0\right)}^{\frac{1}{2}}\;R\left(t\right)\mathrm{Sinh}\left(\frac{1}{2}Gt\right)G^{†}a+\Xi \left(t\right)\Xi^{-1}\left(0\right)\xi \left(0\right), \end{array}$$

where the Cosh and Sinh functions of a matrix *M* are defined by the following absolutely convergent series [48] (Equation (9), p. 122):

$$\begin{array}{ccc} \mathrm{Sinh}(M) & = & M+\sum_{i=1}^{\infty }\frac{M^{2i+1}}{(2i+1)!},\\ \mathrm{Cosh}(M) & = & I+\sum_{i=1}^{\infty }\frac{M^{2i}}{(2i)!}, \end{array}$$

and satisfies the identity ${\mathrm{Sinh}}^{2}\left(M\right)+{\mathrm{Cosh}}^{2}\left(M\right)=I$. The matrix Cosh and Sinh functions can be calculated from the eigendecomposition of $M=O\;\mathrm{diag}\left(\lambda_{1},\ldots ,\lambda_{d}\right)\;O^{⊤}$ as follows:

$$\begin{array}{ccc} \mathrm{Sinh}(M) & = & O\;\mathrm{diag}\left(\sinh\left(\lambda_{1}\right),\ldots ,\sinh\left(\lambda_{d}\right)\right)\;O^{⊤},\;\sinh\left(u\right)=\frac{e^{u}-e^{-u}}{2}=\sum_{i=0}^{\infty }\frac{u^{2i+1}}{(2i+1)!},\\ \mathrm{Cosh}(M) & = & O\;\mathrm{diag}\left(\cosh\left(\lambda_{1}\right),\ldots ,\cosh\left(\lambda_{d}\right)\right)\;O^{⊤},\;\cosh\left(u\right)=\frac{e^{u}+e^{-u}}{2}=\sum_{i=0}^{\infty }\frac{u^{2i}}{(2i)!}. \end{array}$$

When we restrict the manifold to a totally geodesic submanifold $M_{\mu }=\left\{P≻0\right\}$, the geodesic equation becomes $\ddot{P}-\dot{P}P^{-1}\dot{P}=0$, and the geodesic with initial values P(0)=P and $\dot{P}\left(0\right)=S\in \mathrm{Sym}$ is:

$$P\left(t\right)=P^{\frac{1}{2}}\;\exp\left(tP^{-\frac{1}{2}}SP^{-\frac{1}{2}}\right)\;P^{\frac{1}{2}}.$$

The geodesic with boundary values $P\left(0\right)=P_{1}$ and $P\left(1\right)=P_{2}$ is

$$P\left(t\right)=P_{1}^{\frac{1}{2}}\;\exp\left(t\mathrm{Log}(P_{1}^{-\frac{1}{2}}P_{2}P_{1}^{-\frac{1}{2}})\right)\;P_{1}^{\frac{1}{2}}.$$

Furthermore, we can convert a geodesic with boundary values $\gamma_{P}\left(P_{1},P_{2};t\right)$ to an equivalent geodesic with initial values $\gamma_{P}\left(P,S;t\right)$ by letting

$$S=P_{1}^{\frac{1}{2}}\mathrm{Log}\left(P_{1}^{-\frac{1}{2}}P_{2}P_{1}^{-\frac{1}{2}}\right)P_{1}^{\frac{1}{2}}.$$

## Appendix B. Fisher–Rao Distance between Normal Distributions Sharing the Same Covariance Matrix

The Rao distance between $N_{1}=N\left(\mu_{1},\Sigma \right)$ and $N_{2}=N\left(\mu_{2},\Sigma \right)$ has been reported in closed form [42] (Proposition 3). We shall explain the geometric method in full as follows: Let $\left(e_{1},\ldots ,e_{d}\right)$ be the standard frame of $R^{d}$ (ordered basis): The $e_{i}$’s are the unit vectors of the axis $x_{i}$’s. Let *P* be an orthogonal matrix such that $P\;\left(\mu_{2}-\mu_{1}\right)={∥\mu_{2}-\mu_{1}∥}_{2}\;e_{1}$ (i.e., matrix *P* aligns vector $\mu_{2}-\mu_{1}$ to the first axis $x_{1}$). Let $\Delta_{12}={∥\mu_{2}-\mu_{1}∥}_{2}$ be the Euclidean distance between $\mu_{1}$ and $\mu_{2}$. Furthermore, factorize matrix $P\Sigma P^{⊤}$ using the LDL decomposition (a variant of the Cholesky decomposition) as $P\Sigma P^{⊤}=LDL^{⊤}$ where *L* is a lower triangular matrix with all diagonal entries equal to one (lower unitriangular matrix of unit determinant) and *D* a diagonal matrix. Let $\sigma_{12}=\sqrt{D_{11}}$. Then we have [42]:

(A1) $$\rho_{\Sigma }\left(\mu_{1},\mu_{2}\right)=\rho_{N}\left(N\left(\mu_{1},\Sigma \right),N\left(\mu_{2},\Sigma \right)\right)=\rho_{N}\left(N\left(0,\sigma \right),N\left(\Delta_{12}e_{1},\sigma_{12}\right)\right).$$

Please note that the right-hand side term is the Fisher–Rao distance between univariate normal distributions of Equation (7).

To find matrix *P*, we proceed as follows: Let $u=\frac{\mu_{2}-\mu_{1}}{∥\mu_{2}-\mu_{1}{∥}_{2}}$ be the normalized vector to align on axis $x_{1}$. Let $v=u-e_{1}$. Consider the Householder reflection matrix [94]$M=I-\frac{2vv^{⊤}}{{∥v∥}_{2}^{2}}$, where $vv^{⊤}$ is an outer product matrix. Since Householder reflection matrices have determinant −1, we let *P* be a copy of *M* with the last row multiplied by −1 so that we obtain det(P)=1. By construction, we have $Pu=∥\mu_{2}-\mu_{1}{∥}_{2}\;e_{1}$. We then use the affine-invariance property of the Fisher–Rao distance as follows:

$$\begin{array}{ccc} \rho_{N}\left(N\left(\mu_{1},\Sigma \right),N\left(\mu_{2},\Sigma \right)\right) & = & \rho_{N}\left(N\left(0,\Sigma \right),N\left(\mu_{2}-\mu_{1},\Sigma \right)\right),\\ & = & \rho_{N}\left(N\left(0,P\Sigma P^{⊤}\right),N\left(P\left(\mu_{2}-\mu_{1}\right),P\Sigma P^{⊤}\right)\right),\\ & = & \rho_{N}\left(N\left(0,P\Sigma P^{⊤}\right),N\left(\Delta_{12}\;e_{1},P\Sigma P^{⊤}\right)\right),\\ & = & \rho_{N}\left(N\left(0,LDL^{⊤}\right),N\left(\Delta_{12}\;e_{1},LDL^{⊤}\right)\right),\\ & = & \rho_{N}\left(N\left(0,D\right),N\left(\Delta_{12}\;e_{1},D\right)\right). \end{array}$$

The last row follows from the fact that $L^{-1}e_{1}=e_{1}$ since $L^{-1}$ is an upper unitriangular matrix, and $L^{⊤}{\left(L^{-1}\right)}^{⊤}={\left(L^{-1}L\right)}^{⊤}=I$. The right-hand side Fisher–Rao distance is computed from Equation (7).

## Appendix C. Embedding the Set of Multivariate Normal Distributions in a Riemannian Symmetric Space

The multivariate Gaussian manifold N(d) can also be embedded into the SPD cone P(d+1) as a Riemannian symmetric space [82,89] by $f_{\mathrm{SSPD}}$: $\hat{P}=\left\{f_{\mathrm{SSPD}}\left(N\right)\subset P\left(d+1\right)\;:\;N\in N\left(d\right)\right\}$. We have $\hat{P}≅\mathrm{SL}\left(d+1\right)/\mathrm{SO}\left(d+1\right)$ [82,95,96] (and textbook [97], Part II Chapter 10), and the symmetric space SL(d+1)/SO(d+1) can be embedded with the Killing Riemannian metric instead of the Fisher information metric:

$$g^{\mathrm{Killing}}\left(N_{1},N_{2}\right)=\kappa_{\mathrm{Killing}}\;\left(\mu_{1}^{⊤}\Sigma^{-1}\mu_{2}+\frac{1}{2}\mathrm{tr}\left(\Sigma^{-1}\Sigma_{1}\Sigma^{-1}\Sigma_{2}\right)-\frac{1}{2(d+1)}\mathrm{tr}\left(\Sigma^{-1}\Sigma_{1}\right)\mathrm{tr}\left(\Sigma^{-1}\Sigma_{2}\right)\right),$$

where $\kappa_{\mathrm{Killing}}>0$ is a predetermined constant (e.g., 1). The length element of the Killing metric is

$$ds_{\mathrm{SS}}^{2}=\kappa_{\mathrm{Killing}}\;\left(\frac{1}{2}d\mu^{⊤}\Sigma^{-1}d\mu +\mathrm{tr}\left({\left(\Sigma^{-1}d\Sigma \right)}^{2}\right)-\frac{1}{2}{\mathrm{tr}}^{2}\left(\Sigma^{-1}d\Sigma \right)\right).$$

When we consider $N_{\Sigma }$, we may choose $\kappa_{\mathrm{Killing}}=2$ so that the Killing metric coincides with the Fisher information metric. The induced Killing distance [82] is available in closed form:

(A2) $$\rho_{\mathrm{Killing}}\left(N_{1},N_{2}\right)=\sqrt{\kappa_{\mathrm{Killing}}\;\sum_{i=1}^{d+1}\log^{2}\lambda_{i}\left({\hat{L}}_{1}^{-1}{\hat{P}}_{2}{\left({\hat{L}}_{1}^{-1}\right)}^{⊤}\right)},$$

where ${\hat{L}}_{1}$ is the unique lower triangular matrix obtained from the Cholesky decomposition of ${\hat{P}}_{1}=f_{\mathrm{SSPD}}\left(N_{1}\right)={\hat{L}}_{1}{\hat{L}}_{1}^{⊤}$. Please note that ${\hat{L}}_{1}^{-1}{\hat{P}}_{2}{\left({\hat{L}}_{1}^{-1}\right)}^{⊤}\in P\left(d+1\right)$ and $|{\hat{L}}_{1}|$, i.e., ${\hat{L}}_{1}\in \mathrm{SL}\left(d+1\right)$. When $N_{1}=\left(\mu_{1},\Sigma \right)$ and $N_{2}=\left(\mu_{2},\Sigma \right)$ ($N_{1},N_{2}\in N_{\Sigma }$), we have [82]

$$\rho_{\mathrm{Killing}}\left(N_{1},N_{2}\right)=\sqrt{2\kappa_{\mathrm{Killing}}}\mathrm{arccosh}\left(1+\frac{1}{2}\Delta_{\Sigma }^{2}\left(\mu_{1},\mu_{2}\right)\right),$$

where $\Delta_{\Sigma }^{2}$ is the squared Mahalanobis distance. Thus, $\rho_{\mathrm{Killing}}\left(N_{1},N_{2}\right)=h_{\mathrm{Killing}}\left(\Delta_{\Sigma }\left(\mu_{1},\mu_{2}\right)\right)$ where $h_{\mathrm{Killing}}\left(u\right)=\sqrt{2\kappa_{\mathrm{Killing}}}\mathrm{arccosh}\left(1+\frac{1}{2}u^{2}\right)$.

When $N_{1}=\left(\mu ,\Sigma_{1}\right)$ and $N_{2}=\left(\mu ,\Sigma_{2}\right)$ ($N_{1},N_{2}\in N_{\mu }$), we have [82]:

$$\rho_{\mathrm{Killing}}\left(N_{1},N_{2}\right)=\sqrt{\kappa_{\mathrm{Killing}}\left(\sum_{i=1}^{d}\log^{2}\lambda_{i}\left(L_{1}^{-1}P_{2}{\left(L_{1}^{-1}\right)}^{⊤}\right)-\frac{1}{{\left(d+1\right)}^{2}}\left(\sum_{i=1}^{d}\log\lambda_{i}\left(L_{1}^{-1}P_{2}{\left(L_{1}^{-1}\right)}^{⊤}\right)\right)\right)}.$$

See Example 1. Let us emphasize that the Killing distance is not the Fisher–Rao distance but is available in closed form as an alternative metric distance between MVNs.

A Fisher geodesic defect measure of a curve *c* is defined in [89] by

$$\delta \left(c\right)=\lim_{s\to \infty }\frac{1}{s}\int_{0}^{s}{∥\nabla_{\dot{c}}^{g^{\mathrm{Fisher}}}\dot{c}∥}_{c(t)}^{\mathrm{Fisher}}dt,$$

where $\nabla^{g^{\mathrm{Fisher}}}$ denotes the Levi–Civita connection induced by the Fisher metric. When δ(c)=0 the curve is said to be an asymptotic geodesic of the Fisher geodesic. It is proven that Killing geodesics at (μ,Σ) are asymptotic Fisher geodesics when the initial condition $c^{\prime }\left(0\right)$ is orthogonal to $N_{\mu }$.

## Appendix D. Embedding the Set of Multivariate Normal Distributions in the Siegel Upper Space

The Siegel upper space is the space of symmetric complex matrices $Z=X+iY=Z^{⊤}$ with imaginary positive–definite matrices Y≻0 [45,65] (so-called Riemann matrices [98]):

(A3) $$\mathrm{SH}\left(d\right):=\left\{Z=X+iY\;:\;X\in \mathrm{Sym}(d),Y\in P(d)\right\},$$

where Sym(d) is the space of symmetric real d×d matrices. SH(1) corresponds to the Poincaré upper plane. See Figure A2 for an illustration.

The Siegel infinitesimal square line element is

(A4) $$ds_{\mathrm{SH}}^{2}\left(Z\right)=2\mathrm{tr}\left(Y^{-1}dZ\;Y^{-1}d\bar{Z}\right).$$

When X=0 and Z=iY, we have dZ=idY, $d\bar{Z}=-idY$, and it follows that

$$ds_{\mathrm{SH}}^{2}\left(iY\right)=2\mathrm{tr}\left({\left(Y^{-1}dY\right)}^{2}\right).$$

That is, four times the square length of the Fisher matrix of centered normal distributions $ds_{N_{0}}^{2}=\frac{1}{2}\mathrm{tr}\left({\left(P^{-1}dP\right)}^{2}\right)$.

The Siegel distance [45] between $Z_{1}$ and $Z_{2}\in \mathrm{SH}\left(d\right)$ is

(A5) $$\rho_{\mathrm{SH}}\left(Z_{1},Z_{2}\right)=\sqrt{\sum_{i=1}^{d}\log^{2}\left(\frac{1+\sqrt{r_{i}}}{1-\sqrt{r_{i}}}\right)},$$

where

(A6) $$r_{i}=\lambda_{i}\left(R(Z_{1},Z_{2})\right),$$

with $R(Z_{1},Z_{2})$ denoting the matrix generalization of the cross-ratio

(A7) $$R\left(Z_{1},Z_{2}\right):=\left(Z_{1}-Z_{2}\right){\left(Z_{1}-{\bar{Z}}_{2}\right)}^{-1}\left({\bar{Z}}_{1}-{\bar{Z}}_{2}\right){\left({\bar{Z}}_{1}-Z_{2}\right)}^{-1},$$

and $\lambda_{i}\left(M\right)$ denoting the *i*-th largest (real) eigenvalue of (complex) matrix *M*. (In practice, we numerically must round off the tiny imaginary parts to obtain proper real eigenvalues [65].) The Siegel upper half space is a homogeneous space where the Lie Group SU(d,d)/S(U(d)×U(d)) acts transitively on it.

We can embed a multivariate normal distribution N=(μ,Σ) into SH(d) as follows:

$$N\left(\mu ,\Sigma \right)\to Z\left(N\right):=\left(\mu \mu^{⊤}+i\Sigma \right),$$

and consider the Siegel distance on the embedded normal distributions as another potential metric distance between multivariate normal distributions:

(A8) $$\rho_{\mathrm{SH}}\left(N_{1},N_{2}\right)=\rho_{\mathrm{SH}}\left(Z\left(N_{1}\right),Z\left(N_{2}\right)\right).$$

Notice that the real matrix part of the Z(N)’s are all of rank one by construction.

## Appendix E. The Symmetrized Bregman Divergence Expressed as Integral Energies on Dual Geodesics

Let $S_{F}\left(\theta_{1};\theta_{2}\right)=B_{F}\left(\theta_{1}:\theta_{2}\right)+B_{F}\left(\theta_{2}:\theta_{1}\right)$ be a symmetrized Bregman divergence. Let $ds^{2}=d\theta^{⊤}\nabla^{2}F\left(\theta \right)d\theta$ denote the squared length element on the Bregman manifold and denote by γ(t) and $\gamma^{*}\left(t\right)$ the dual geodesics connecting $\theta_{1}$ to $\theta_{2}$. We can express $S_{F}\left(\theta_{1};\theta_{2}\right)$ as integral energies on dual geodesics:

**Property A1.** 
*We have $S_{F}\left(\theta_{1};\theta_{2}\right)=\int_{0}^{1}ds^{2}\left(\gamma \left(t\right)\right)dt=\int_{0}^{1}ds^{2}\left(\gamma^{*}\left(t\right)\right)dt$.*

**Proof.** The proof that the symmetrized Bregman divergence amount to these energy integrals is based on the first-order and second-order directional derivatives. The first-order directional derivative $\nabla_{u}F\left(\theta \right)$ with respect to vector *u* is defined by

$$\nabla_{u}F\left(\theta \right)=\lim_{t\to 0}\frac{F(\theta +tv)-F(\theta )}{t}=v^{⊤}\nabla F\left(\theta \right).$$

The second-order directional derivatives $\nabla_{u,v}^{2}F\left(\theta \right)$ is

$$\begin{array}{ccc} \nabla_{u,v}^{2}F\left(\theta \right) & = & \nabla_{u}\nabla_{v}F\left(\theta \right),\\ & = & \lim_{t\to 0}\frac{v^{⊤}\nabla F\left(\theta +tu\right)-v^{⊤}\nabla F\left(\theta \right)}{t},\\ & = & u^{⊤}\nabla^{2}F\left(\theta \right)v. \end{array}$$

Now consider the squared length element $ds^{2}\left(\gamma \left(t\right)\right)$ on the primal geodesic γ(t) expressed using the primal coordinate system θ: $ds^{2}\left(\gamma \left(t\right)\right)=d\theta {\left(t\right)}^{⊤}\nabla^{2}F\left(\theta \left(t\right)\right)d\theta \left(t\right)$ with $\theta \left(\gamma \left(t\right)\right)=\theta_{1}+t\left(\theta_{2}-\theta_{1}\right)$ and $d\theta \left(t\right)=\theta_{2}-\theta_{1}$. Let us express the $ds^{2}\left(\gamma \left(t\right)\right)$ using the second-order directional derivative:

$$ds^{2}\left(\gamma \left(t\right)\right)=\nabla_{\theta_{2}-\theta_{1}}^{2}F\left(\theta \left(t\right)\right).$$

Thus, we have $\int_{0}^{1}ds^{2}\left(\gamma \left(t\right)\right)dt={\left[\nabla_{\theta_{2}-\theta_{1}}F\left(\theta \left(t\right)\right)\right]}_{0}^{1}$, where the first-order directional derivative is $\nabla_{\theta_{2}-\theta_{1}}F\left(\theta \left(t\right)\right)={\left(\theta_{2}-\theta_{1}\right)}^{⊤}\nabla F\left(\theta \left(t\right)\right)$. Therefore we obtain $\int_{0}^{1}ds^{2}\left(\gamma \left(t\right)\right)dt={\left(\theta_{2}-\theta_{1}\right)}^{⊤}\left(\nabla F\left(\theta_{2}\right)-\nabla F\left(\theta_{1}\right)\right)=S_{F}\left(\theta_{1};\theta_{2}\right)$. Similarly, we express the squared length element $ds^{2}\left(\gamma^{*}\left(t\right)\right)$ using the dual coordinate system η as the second-order directional derivative of $F^{*}\left(\eta \left(t\right)\right)$ with $\eta \left(\gamma^{*}\left(t\right)\right)=\eta_{1}+t\left(\eta_{2}-\eta_{1}\right)$:

$$ds^{2}\left(\gamma^{*}\left(t\right)\right)=\nabla_{\eta_{2}-\eta_{1}}^{2}F^{*}\left(\eta \left(t\right)\right).$$

Therefore, we have $\int_{0}^{1}ds^{2}\left(\gamma^{*}\left(t\right)\right)dt={\left[\nabla_{\eta_{2}-\eta_{1}}F^{*}\left(\eta \left(t\right)\right)\right]}_{0}^{1}=S_{F^{*}}\left(\eta_{1};\eta 2\right)$. Since $S_{F^{*}}\left(\eta_{1};\eta_{2}\right)=S_{F}\left(\theta_{1};\theta_{2}\right)$, we conclude that

$$S_{F}\left(\theta_{1};\theta_{2}\right)=\int_{0}^{1}ds^{2}\left(\gamma \left(t\right)\right)dt=\int_{0}^{1}ds^{2}\left(\gamma^{*}\left(t\right)\right)dt$$

Please note that in 1D, both pregeodesics γ(t) and $\gamma^{*}\left(t\right)$ coincide. We have $ds^{2}\left(t\right)={\left(\theta_{2}-\theta_{1}\right)}^{2}f^{\prime \prime }\left(\theta \left(t\right)\right)=\left(\eta_{2}-\eta_{1}\right){f^{*}}^{\prime \prime }\left(\eta \left(t\right)\right)$ so that we check that $S_{F}\left(\theta_{1};\theta_{2}\right)=\int_{0}^{1}ds^{2}\left(\gamma \left(t\right)\right)dt=\left(\theta_{2}-\theta_{1}\right){\left[f^{\prime }\left(\theta \left(t\right)\right)\right]}_{0}^{1}=\left(\eta_{2}-\eta_{1}\right){\left[{f^{*}}^{\prime }\left(\eta \left(t\right)\right)\right]}_{0}^{1}=\left(\eta_{2}-\eta_{1}\right)\left(\theta_{2}-\theta_{2}\right)$. □
